# A natural experiment on the effect of herpes zoster vaccination on dementia

**DOI:** 10.1038/s41586-025-08800-x

**Published:** 2025-04-02

**Authors:** Markus Eyting, Min Xie, Felix Michalik, Simon Heß, Seunghun Chung, Pascal Geldsetzer

**Affiliations:** 1 Division of Primary Care and Population Health, Department of Medicine, Stanford University, Stanford, CA USA; 2 Leibniz Institute for Financial Research SAFE, Frankfurt am Main, Germany; 3 Faculty of Law and Economics, Johannes Gutenberg University Mainz, Mainz, Germany; 4 Heidelberg Institute of Global Health (HIGH), Heidelberg University Hospital, Heidelberg, Germany; 5 Department of Economics, Vienna University of Economics and Business, Vienna, Austria; 6 Department of Epidemiology and Population Health, Stanford University, Stanford, CA USA; 7 The Phil and Penny Knight Initiative for Brain Resilience at the Wu Tsai Neurosciences Institute, Stanford University, Stanford, CA USA; 8 Chan Zuckerberg Biohub – San Francisco, San Francisco, CA USA

**Keywords:** Dementia, Dementia, Viral infection

## Abstract

Neurotropic herpesviruses may be implicated in the development of dementia^1–5^. Moreover, vaccines may have important off-target immunological effects^6–9^. Here we aim to determine the effect of live-attenuated herpes zoster vaccination on the occurrence of dementia diagnoses. To provide causal as opposed to correlational evidence, we take advantage of the fact that, in Wales, eligibility for the zoster vaccine was determined on the basis of an individual’s exact date of birth. Those born before 2 September 1933 were ineligible and remained ineligible for life, whereas those born on or after 2 September 1933 were eligible for at least 1 year to receive the vaccine. Using large-scale electronic health record data, we first show that the percentage of adults who received the vaccine increased from 0.01% among patients who were merely 1 week too old to be eligible, to 47.2% among those who were just 1 week younger. Apart from this large difference in the probability of ever receiving the zoster vaccine, individuals born just 1 week before 2 September 1933 are unlikely to differ systematically from those born 1 week later. Using these comparison groups in a regression discontinuity design, we show that receiving the zoster vaccine reduced the probability of a new dementia diagnosis over a follow-up period of 7 years by 3.5 percentage points (95% confidence interval (CI) = 0.6–7.1, *P* = 0.019), corresponding to a 20.0% (95% CI = 6.5–33.4) relative reduction. This protective effect was stronger among women than men. We successfully confirm our findings in a different population (England and Wales’s combined population), with a different type of data (death certificates) and using an outcome (deaths with dementia as primary cause) that is closely related to dementia, but less reliant on a timely diagnosis of dementia by the healthcare system^10^. Through the use of a unique natural experiment, this study provides evidence of a dementia-preventing or dementia-delaying effect from zoster vaccination that is less vulnerable to confounding and bias than the existing associational evidence.

## Abstract (web summary)

Using a natural experiment that avoids common bias concerns, this study finds that the live-attenuated shingles vaccine reduced the probability of a new dementia diagnosis within a follow-up period of 7 years by approximately one-fifth.

## Main

Recently, evidence has grown that neurotropic herpesviruses may have a role in the pathogenesis of dementia^1–5^. One approach to targeting herpesviruses is vaccination. However, vaccines are also increasingly being recognized as eliciting a broader immune response that can have important off-target effects, particularly in the case of live-attenuated vaccines^6–9^. Such effects have frequently been observed to differ strongly by sex^7^.

To date, studies in cohort and electronic health record data on the effect of vaccination receipt on dementia have simply compared the occurrence of dementia among those who received a given vaccination and those who did not^11^. These studies have to assume that all characteristics that are different between those who are vaccinated and those who are not (and that are also related to dementia) have been sufficiently well measured and modelled in the analysis, such that no factors confound the relationship between vaccination receipt and dementia^12^. This assumption of no confounding bias is often implausible because it has to be assumed that the study has detailed data on factors that are difficult to measure, such as personal motivation or health literacy^13^. It is also an assumption that cannot be empirically verified.

We used a fundamentally different approach that takes advantage of the fact that, in Wales, starting on 1 September 2013, those born on or after 2 September 1933 were eligible for herpes zoster vaccination for at least 1 year, while those born earlier never became eligible^14^. Using detailed large-scale electronic health record data, we were able to compare adults who were ineligible for the vaccine because they were born immediately before the eligibility cut-off date with those born immediately after who were eligible. Importantly, individuals who are only a few weeks apart in age are not expected to differ systematically from each other. That is, all potential confounding variables are in expectation balanced between our comparison groups. By taking advantage of this unique natural experiment, we were able to avoid confounding more credibly than all existing studies on the topic^15–24^, which have simply compared vaccine recipients to non-recipients while trying to control for the myriad of differences between these groups.

Adults born immediately after the 2 September 1933 date-of-birth eligibility cut-off had a 47.2 percentage point higher probability (from 0.01% to 47.2%) of ever receiving the herpes zoster vaccine than those born immediately before this cut-off date. As expected, other than this abrupt change in herpes zoster vaccination uptake, patients were balanced across the 2 September 1933 date-of-birth eligibility threshold in their uptake of other preventive health services, past common disease diagnoses and educational attainment. We then used this ‘quasi-randomization’ in a regression discontinuity analysis to first replicate the known finding from clinical trials that the herpes zoster vaccine reduces new diagnoses of shingles. Second, we extended this approach to an outcome—dementia—that was never assessed in clinical trials of the herpes zoster vaccine, and find that the vaccine reduces the probability of a new dementia diagnosis over a seven-year follow-up period by approximately one-fifth. Third, we show that the herpes zoster vaccine did not affect the occurrence of any other common causes of mortality or morbidity other than shingles and dementia. Similarly, we show that receipt of the herpes zoster vaccine did not lead to increased uptake of other vaccinations or preventive health measures. Fourth, we provide evidence that no other intervention (such as health insurance eligibility) in Wales used the identical date of birth (2 September 1933) as eligibility cut-off as was used to define eligibility for the herpes zoster vaccine. Fifth, we show that all findings remain similar when using a different analysis approach. Sixth, we show that changes in healthcare pathways as a result of a shingles episode are unlikely to explain our findings. Seventh, we provide exploratory evidence from our electronic health record data on the mechanism through which herpes zoster vaccination could affect dementia. Our study focuses on the live-attenuated herpes zoster vaccine (Zostavax; hereafter, zoster vaccine), because the newer recombinant subunit zoster vaccine (Shingrix) became available in the UK only after our follow-up period ended^25^.

## Difference in zoster vaccination receipt

We used the Secure Anonymised Information Linkage (SAIL) Databank^26^, which contains detailed electronic health record data on primary care visits from approximately 80% of primary care providers in Wales, linked to secondary care records and the country’s death register data. The study population for our primary analyses consisted of all adults born between 1 September 1925 and 1 September 1942 who were registered with a primary care provider (which is the case for over 98% of adults residing in Wales^27^), resided in Wales and did not have a diagnosis of dementia at the time of the start of the zoster vaccine program in Wales (on 1 September 2013). Basic sociodemographic and clinical characteristics of the sample of 282,541 adults in our primary analysis cohort are shown in Supplementary Table 1.

In Wales, individuals born between 2 September 1933 and 1 September 1934 (16,595 adults in our data) became eligible for the zoster vaccine for at least 1 year on 1 September 2013. Eligibility was then progressively extended to younger, but not older, age cohorts annually on the basis of their exact date of birth (Methods).

**Fig. 1:**
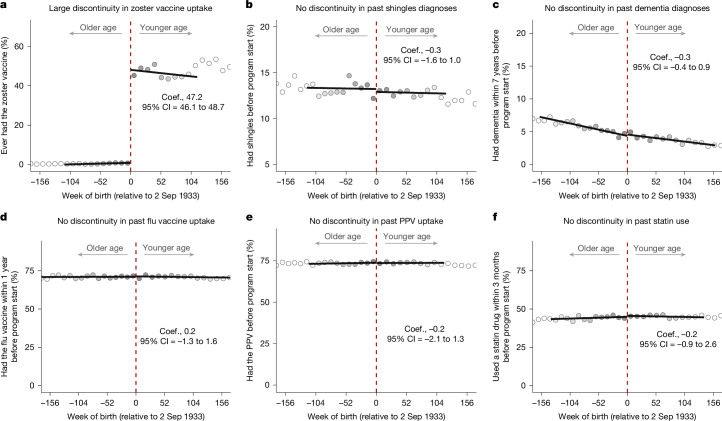
A large jump in zoster vaccine receipt at the date-of-birth eligibility threshold. **a**–**f**, The date-of-birth eligibility cut-off led to a large discontinuity in zoster vaccine receipt (**a**), but there is baseline exchangeability across the cut-off for uptake of other preventive interventions (flu vaccine (**d**), pneumococcal polysaccharide vaccine (PPV) (**e**) and statin medications (**f**)) as well as past shingles (**b**) and dementia (**c**) diagnoses. The data source for this analysis was the SAIL database for Wales. All analyses were run on the same sample as those for the effect of the zoster vaccine on dementia occurrence. The exception is **c**, for which we did not exclude individuals with a diagnosis of dementia before the start of the zoster vaccine program. The grey dots show the mean value for each 10-week increment in week of birth. The grey shading of the dots is proportionate to the weight that observations from this 10-week increment received in the analysis.

We find that being born just 1 week after 2 September 1933, and therefore being eligible for the zoster vaccine for at least 1 year, caused an abrupt increase in the probability of ever receiving the zoster vaccine from 0.01% to 47.2% (*P* < 0.001; Fig. 1). This provides a unique opportunity to avoid confounding concerns because it is unlikely that individuals born immediately around the date-of-birth eligibility threshold systematically differ from each other by anything but a one-week difference in age and a large difference in the probability of receiving the zoster vaccine. We substantiate this empirically by showing that, at the time of the start date of the zoster vaccination program, neither the prevalence of common disease diagnoses (including having been diagnosed with dementia before the vaccination program rollout), dementia risk as predicted from a series of clinical and sociodemographic variables, nor the prevalence of preventive behaviours (other than zoster vaccine uptake) display a discontinuity at the date-of-birth eligibility threshold for the zoster vaccine (Fig. 1 and Supplementary Figs. 1–4). Thus, after flexibly controlling for age, our two comparison groups (one with a low and one with a high probability of receiving the zoster vaccine) born immediately on either side of the 2 September 1933 date-of-birth eligibility threshold are probably exchangeable with each other on all observed and unobserved potential confounding variables.

Our analysis approach primarily compares those who were ineligible for zoster vaccination because they had their 80th birthday immediately before the program’s start date with those who were eligible because they had their 80th birthday immediately after the start date. As is standard practice in regression discontinuity analyses^28,29^, the effect of actually receiving the vaccine (as opposed to merely being eligible) was determined using a two-stage least-squares regression, which divides the magnitude of the abrupt change in the outcome at the date-of-birth eligibility threshold by the magnitude of the abrupt change in vaccine uptake at this threshold (Methods). Thus, the fact that not all those who were eligible received zoster vaccination does not bias our analysis.

## Zoster vaccination prevents shingles

**Fig. 2:**
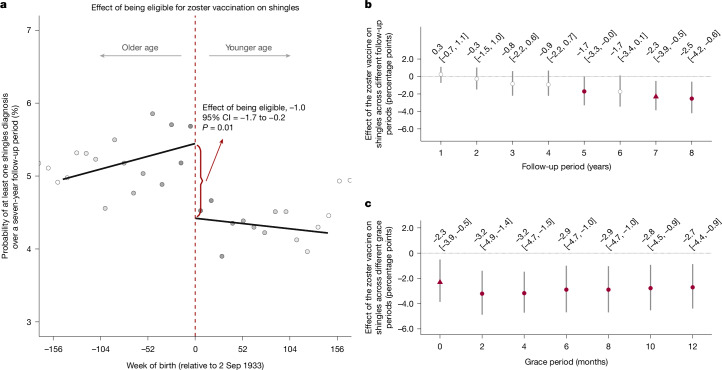
The effect of the zoster vaccine on shingles diagnoses. **a**–**c**, Effect estimates of being eligible for (**a**), and having received (across different follow-up periods (**b**) and across different grace periods (**c**)), the zoster vaccine on the probability of having at least one shingles diagnosis during the follow-up period. For **a**, the MSE-optimal bandwidth is 145.7 weeks (95,227 adults). The grey dots show the mean value for each 10-week increment in week of birth. The grey shading of the dots is proportionate to the weight that observations from this 10-week increment received in the analysis. For **b** and **c**, the MSE-optimal bandwidth for our primary specification is 116.9 weeks (76,316 adults). The triangles (rather than points) depict our primary specification. The red (as opposed to white) fillings denote statistical significance (*P* < 0.05). Grace periods refer to time periods since the index date after which the follow-up time is considered to begin. The grey vertical bars show the 95% CIs around the point estimate of the regression coefficient (two-sided *t* tests).

We first demonstrate that our approach successfully reproduces the known causal effect from clinical trials that the vaccine reduces the occurrence of shingles^30^. Specifically, using a regression discontinuity design^28,29^, we compared the occurrence of shingles between adults born close to either side of the date-of-birth eligibility threshold for the zoster vaccine. Consistent with the approach used by clinical trials of the zoster vaccine^30^, our outcome was whether or not an individual had at least one shingles diagnosis during the follow-up period. During our follow-up period of 7 years, a total of 14,465 among 296,324 adults in our sample had at least one diagnosis of shingles. Over the same follow-up time, we find that being eligible for the vaccine reduced the probability of having at least one shingles diagnosis by 1.0 (95% CI = 0.2–1.7; *P* = 0.010) percentage point (Fig. 2a), corresponding to a relative reduction of 18.8% (95% CI = 8.8–28.9). When calculating the effect of actually receiving the zoster vaccine, we find a reduction in the probability of having at least one shingles diagnosis of 2.3 (95% CI = 0.5–3.9; *P* = 0.011) percentage points over the seven-year follow-up period (Fig. 2b); an effect (37.2% (95% CI = 19.7–54.7) in relative terms) that is similar in size to that observed in clinical trials of the live-attenuated zoster vaccine (Zostavax)^30^.

We show that our estimated effect is not sensitive to the chosen functional form of the regression used to model the relationship of shingles occurrence with week of birth (Supplementary Fig. 5), the width of the week-of-birth window (bandwidth) around the date-of-birth eligibility cut-off that defines our analysis sample (Supplementary Fig. 6a) or to different grace periods (Fig. 2c). With ‘grace periods’, we refer to time periods since the index date after which the follow-up time is considered to begin (Methods). There was also a strong indication that the zoster vaccine reduced the probability of having at least one diagnosis of postherpetic neuralgia (a common complication of shingles), although this effect did not reach statistical significance in all specifications (Supplementary Fig. 7).

## New diagnoses of dementia

Given the neuropathological overlap between dementia types and the difficulty in distinguishing dementia types clinically^31^, as well as our reduced statistical power when studying less-common outcomes, we defined dementia as dementia of any type or cause as our outcome. We considered an individual to have developed dementia if there was a new diagnosis of dementia in our electronic health record data (which includes all diagnoses made in primary or secondary care) or dementia was listed as a primary or contributory cause of death on the death certificate. The Read and ICD-10-codes used to define dementia are listed in the Supplementary Codes. During our seven-year follow-up period, 35,307 among 282,541 adults in our sample were newly diagnosed with dementia.

**Fig. 3:**
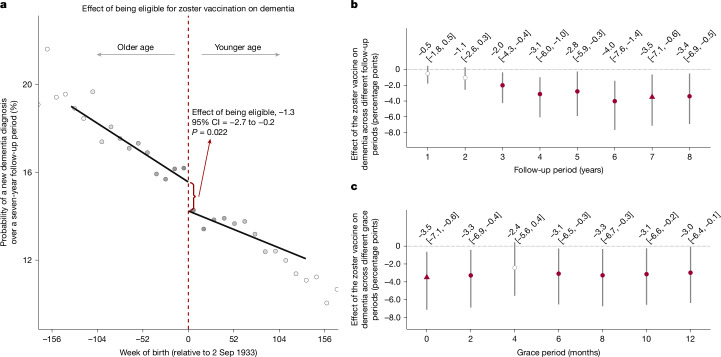
The effect of the zoster vaccine on new diagnoses of dementia. **a**–**c**, Effect estimates of being eligible for (**a**), and having received (across different follow-up periods (**b**) and across different grace periods (**c**)), the zoster vaccine on new diagnoses of dementia. For **a**, the MSE-optimal bandwidth is 134.4 weeks (83,167 adults). The grey dots show the mean value for each 10-week increment in week of birth. The grey shading of the dots is proportionate to the weight that observations from this 10-week increment received in the analysis. For **b** and **c**, the MSE-optimal bandwidth for our primary specification is 90.6 weeks (56,098 adults). The triangles (rather than points) depict our primary specification. The red (as opposed to white) fillings denote statistical significance (*P* < 0.05). Grace periods refer to time periods since the index date after which the follow-up time is considered to begin. The grey vertical bars show the 95% CIs around the point estimate of the regression coefficient (two-sided *t* tests).

Using our regression discontinuity approach, we estimate that the effect of being eligible for the zoster vaccine is a 1.3 (95% CI = 0.2–2.7; *P* = 0.022) percentage points absolute and 8.5% (95% CI = 1.9–15.1) relative reduction in the probability of a new dementia diagnosis over 7 years (Fig. 3a). Scaled to account for the fact that not all those who were eligible received the vaccine, we find that actually receiving the zoster vaccine reduced the probability of a new dementia diagnosis by 3.5 (95% CI = 0.6–7.1; *P* = 0.019) percentage points, corresponding to a relative reduction of 20.0% (95% CI = 6.5–33.4) (Fig. 3b). The effect estimates were generally not sensitive to different grace periods (Fig. 3c), the functional form of our regressions (Supplementary Fig. 8) nor the width of the week-of-birth window (bandwidth) drawn around the date-of-birth eligibility cut-off (Supplementary Fig. 6b). We also find significant effects of the zoster vaccine on reducing dementia diagnoses if a diagnosis is defined solely as a new prescription of a medication (donepezil hydrochloride, galantamine, rivastigmine or memantine hydrochloride) that is frequently prescribed to slow the progression of Alzheimer’s disease (Supplementary Table 2 (column 2)). Similarly, the effects remain similar when adjusting for all input variables to the Dementia Risk Score^32^ (as recorded before 1 September 2013) (Supplementary Table 2 (column 7)).

## Other interventions using an identical cut-off

The key strength of our study is that a confounding variable can bias our analysis only if the variable changes abruptly at the 2 September 1933 date-of-birth threshold^28,29^. Thus, confounding bias could occur if another intervention also used the date of birth cut-off of 2 September 1933 as an eligibility criterion. Such an intervention is unlikely to affect only the risk of developing dementia without also influencing other health outcomes. We therefore implemented the same regression discontinuity approach as we did for shingles and dementia for the ten leading causes of disability-adjusted life years and mortality for the age group 70+ years in Wales in 2019^33^, and all conditions that are part of the Charlson Comorbidity Index^34^. As shown in Supplementary Figs. 9 and 10, we generally do not detect effects of zoster vaccination on new diagnoses of these other common health outcomes.

We undertook four additional types of analysis, all of which provide evidence against another intervention having used the identical day-month-year combination (2 September 1933) as was used as the date-of-birth eligibility threshold for the zoster vaccine rollout. First, we show that the 2 September 1933 date-of-birth threshold does not affect the probability of taking up other preventive health interventions (Supplementary Fig. 11). Second, we examined whether the day–month (that is, 2 September) date-of-birth cut-off used for zoster vaccine eligibility was also used by other interventions that affect dementia risk. We did so by implementing the identical analysis as for 1 September 2013 (the actual date on which the zoster vaccine program started) for 1 September of each of the three years before and after 2013. Thus, for example, when shifting the start date of the program to 1 September 2012, we compared those around the 2 September 1932 date-of-birth threshold with the follow-up period starting on 1 September 2012. As an additional check that enabled us to maintain the length of the seven-year follow-up period used in our primary analyses, we shifted the program start date to 1 September of each of the 6 years preceding (but not after) 2013. As expected, for both of these checks, we find a significant effect on dementia occurrence only for the date-of-birth cutoff (2 September 1933) that was actually used by the zoster vaccination program (Supplementary Figs. 12 and 13). Third, we find that there is no difference in the seven-year incidence of dementia between age cohorts around the 2 September 1933 date-of-birth threshold for the seven-year period before the zoster vaccine rollout (Supplementary Fig. 14). Fourth, using data from the 2011 Census, we show in Supplementary Figs. 15–17 that there are no discontinuities across the 2 September 1933 threshold in the proportion of individuals in Wales who reached a particular level of education.

## Robustness to a different analytical approach

**Fig. 4:**
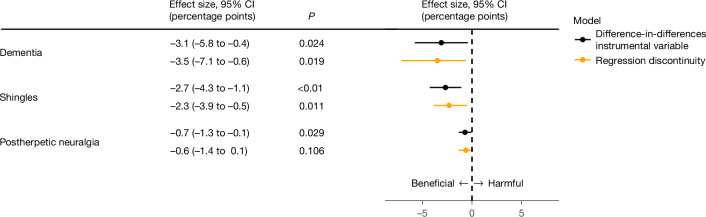
Comparison of effect estimates between the DID-IV and regression discontinuity approach. Comparison of absolute effect estimates of having received the zoster vaccine on new diagnoses of dementia, shingles and postherpetic neuralgia between the DID-IV and the regression discontinuity analyses. The data source for this analysis was the SAIL database for Wales. The sample size for the dementia outcome is 96,767 adults and the sample for the shingles and postherpetic neuralgia outcomes is 105,258 adults. *P* values were calculated using two-sided *t*-tests. The *P* value for the DID-IV effect on shingles is 0.001. The error bars depict the 95% CIs around the point estimate of the regression coefficient (two-sided *t*-tests).

As an additional test of the robustness of our findings, we implemented all primary analyses using a difference-in-differences instrumental variable analysis (DID-IV) that takes advantage of the fact that the only 2 September date-of-birth threshold at which we would expect an abrupt change in the outcome is the 2 September threshold in 1933 (that is, the day–month–year combination that was used as eligibility cut-off by the zoster vaccination program). In doing so, our analysis relaxes the continuity assumption of regression discontinuity (that is, the assumption that potential confounding variables do not display a sudden change at the 2 September 1933 date-of-birth eligibility threshold), and instead assumes that (in the absence of the zoster vaccination program) a possible discontinuity in the outcome at the 2 September 1933 threshold is not different in size from a discontinuity at the 2 September threshold in previous birth years. Details of our approach are provided in the Methods. Encouragingly, the effect of zoster vaccine receipt on the probability of a new dementia diagnosis during our seven-year follow-up period is remarkably similar between the DID-IV and regression discontinuity approach (−3.1 (95% CI = −5.8 to −0.4, *P* = 0.024) versus −3.5 (95% CI = −7.1 to −0.6, *P* = 0.019) percentage points) (Fig. 4). This is also the case for the outcomes of shingles and postherpetic neuralgia (Fig. 4). We conducted the same checks for balance in health characteristics between our comparison groups for the DID-IV as we implemented for our regression discontinuity analyses (Supplementary Fig. 18). We also verified that our DID-IV approach yields significant effects only for the outcomes of dementia, shingles and postherpetic neuralgia, but not for any other common health outcomes (Supplementary Fig. 18).

## Explorations of the effect mechanism

A protective effect of zoster vaccination on dementia diagnoses could arise from three (non-mutually exclusive) mechanisms: (1) changes in healthcare pathways as a result of a shingles episode; (2) a reduction in reactivations of the varicella zoster virus (VZV); and (3) a VZV-independent immunomodulatory effect (for example, one mediated through heterologous adaptive immunity or trained innate immunity). In this section, we present evidence to examine each of these mechanisms.

### Changes in healthcare after shingles

Reduced healthcare use resulting averted shingles episodes from zoster vaccination receipt could have translated to fewer opportunities for the health system to (1) diagnose dementia (ascertainment bias); or (2) implement care changes (for example, initiation of a new medication) that increase the risk of being diagnosed with dementia in the future. It is important to point out that this mechanism is unlikely to fully explain our findings, because the size of our effect estimates for reductions in shingles episodes from zoster vaccination were considerably too small to plausibly account for the observed reduction in dementia diagnoses.

We nonetheless conducted five types of analysis to examine this potential mechanism further. First, if shingles episodes presented an opportunity for the health system to diagnose dementia, then they would probably also present an opportunity to diagnose other chronic conditions. We therefore applied the same regression discontinuity approach as for shingles and dementia to all chronic conditions that are either among the ten leading causes of disability-adjusted life years and mortality for the age group 70+ years in Wales in 2019^33^ or part of the Charlson Comorbidity Index^34^. We plotted our estimates across one-year increments in the follow-up period. With the exception of rheumatological diseases, we show that being eligible for the zoster vaccine did not have an effect on new chronic disease diagnoses (Supplementary Fig. 19). Second, we adjusted our regressions for the frequency of health service use (the number of primary care visits, outpatient visits, hospital admissions and influenza vaccinations received) during the follow-up period, which did not substantially change our effect estimates (Supplementary Table 2 (column 4)). Third, we implemented our analyses when restricting the analysis cohort to the 247,784 (87.6% of the analysis cohort for our primary analyses) patients who visited their primary care provider at least once a year during each of the 5 years before the start of the zoster vaccine rollout. The rationale for this analysis is that, among patients who already interact frequently with the health system, a reduction of one further contact with the health system due to an averted shingles episode is less likely to affect the probability of detecting undiagnosed dementia. The effect sizes among this cohort of frequent healthcare users remain similar to those in our primary analytical cohort (Supplementary Table 2 (column 3)). Fourth, we added whether individuals experienced a shingles episode during the follow-up period as a covariate in our primary regression discontinuity analysis. We found that adjusting our analysis for shingles episodes did not substantially change our point estimate (Supplementary Fig. 20). Fifth, we implemented an event study among those participants in the mean-squared-error (MSE)-optimal bandwidth of our primary regression discontinuity analysis for dementia who received a shingles diagnosis during the follow-up period. To investigate whether episodes of shingles led to changes in healthcare received by patients, we examined the effect of the shingles diagnosis on the following outcomes in each of the 36 months after the diagnosis: (1) the probability of receiving a new dementia diagnosis; (2) a set of indicators of health service use; (3) the probability of receiving a new medication prescription for antiviral drugs, opioid medications, gabapentin or pregabalin, and any of 216 medications that were associated with an increased risk of dementia in another analysis in the SAIL database^23^; and (4) the probability of being diagnosed with any of the chronic conditions that are part of the Charlson Comorbidity Index^34^. We found that shingles diagnoses did not increase the probability of receiving a new dementia diagnosis in the months after the shingles diagnosis, and led to only short-term increases in healthcare service use and new medication prescriptions (Supplementary Fig. 21). The increase in the probability of receiving a gabapentin or pregabalin prescription in the months after the shingles episode, while more sustained, was small in magnitude. Similarly, the increase in the probability of being diagnosed with any chronic condition in the month of a shingles episode compared with the month before the episode was less than one percentage point (Supplementary Fig. 21).

As the effect of zoster vaccination on shingles episodes is moderate (Fig. 2), and the five types of analysis in this section document only small and short-lived effects of shingles episodes on healthcare pathways, even the most conservative assumptions about the effect of these care paths on dementia imply that changes in healthcare as a result of a shingles episode cannot explain our findings.

### Reduction in reactivations of VZV

As described in the previous section, adjusting our regression discontinuity analysis for whether a patient had a record of at least one shingles episode during the follow-up period did not change our point estimate substantially (Supplementary Fig. 20). However, conclusions from this analysis regarding reductions in VZV reactivations as the effect mechanism are limited by the fact that (1) zoster vaccination probably reduces both clinical as well as subclinical reactivations of VZV^30,35^; and (2) having a shingles episode may be an unreliable indicator of the degree of subclinical VZV reactivations experienced during the entire follow-up period, given that shingles episodes may boost immunity for VZV^30,35^. We therefore conducted the following analyses to further examine reductions in VZV reactivations as the effect mechanism.

First, we examined the time during the follow-up period at which the effect of zoster vaccination on dementia appears to begin. Specifically, among patients who were born in close proximity to the 2 September 1933 date-of-birth threshold, we plotted the Kaplan–Meier and cumulative incidence curves for dementia for those who were eligible versus ineligible for zoster vaccination (Methods). If the effect mechanism is through a reduction in VZV reactivations, then one would expect that the effects of the vaccine on reductions in clinical and subclinical reactivations of the virus would begin before observing an effect on dementia. The live-attenuated zoster vaccine is thought to begin being efficacious within weeks after vaccine administration^30,36^. Consistent with the principle that the effect on VZV reactivations should precede the dementia effect, we observe that the reduction in the incidence of dementia begins to emerge only after more than one year, both among the full population as well as among women only (Supplementary Fig. 22).

Second, while a shingles episode may boost VZV immunity and, therefore, reduce subsequent subclinical VZV reactivations^30,35^, individuals who experience multiple episodes as opposed to a single shingles episode during the follow-up period probably experience a greater degree of both clinical and subclinical VZV reactivations during the follow-up period^30^. Using propensity score matching (Methods), we therefore compared the association with dementia from experiencing multiple versus a single shingles episode. We find a higher incidence of dementia among those who experienced multiple shingles episodes (Supplementary Fig. 23).

Third, if VZV reactivations increase the risk of dementia, then limiting the degree of replication of the virus during a shingles episode through antiviral medication could be expected to decrease dementia incidence. Using a multivariable Cox proportional hazards model (Methods), we therefore compared the association with dementia between individuals whose shingles episode was treated with antiviral medication and those for whom the episode was untreated. We find that antiviral treatment of a shingles episode is associated with a reduced incidence of dementia (Supplementary Fig. 23).

### VZV-independent immunomodulatory effect

**Fig. 5:**
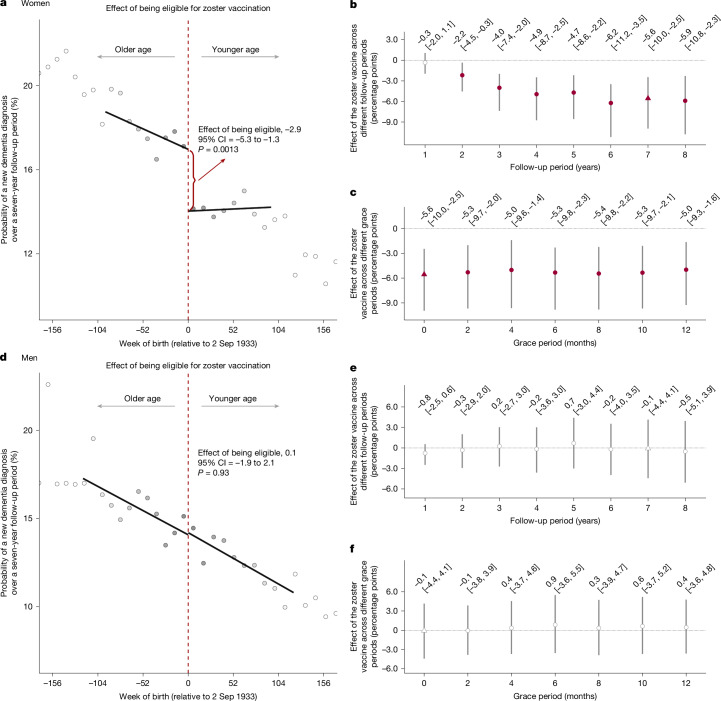
The effect of the zoster vaccine on new diagnoses of dementia, separately for women and men. **a**–**f**, Effect estimates of being eligible for (**a** (women) and **d** (men)) and having received (**b** and **c** (women) and **e** and **f** (men); across different follow-up periods (**b** and **e**) and across different grace periods (**c** and **f**)) the zoster vaccine on new diagnoses of dementia, separately for women and men. The data source for this analysis was the SAIL database for Wales. The triangles (rather than points) depict our primary specification. Red (as opposed to white) fillings denote statistical significance (*P* < 0.05). Grace periods refer to time periods since the index date after which the follow-up time is considered to begin. The grey vertical bars depict the 95% CIs around the point estimate of the regression coefficient (two-sided *t*-test). The grey dots show the mean value for each 10-week increment in week of birth. For **a**, among women, the MSE-optimal bandwidth is 95.5 weeks (32,601 women). For **b** and **c**, among women, the MSE-optimal bandwidth for our primary specification is 149.1 weeks (50,816 women). For **d**, among men, the MSE-optimal bandwidth for our primary specification is 121.3 weeks (33,725 men). For **e** and **f**, among men, the MSE-optimal bandwidth for our primary specification is 91.8 weeks (25,563 men). The grey shading of the dots is proportionate to the weight that observations from this 10-week increment received in the analysis.

To probe this mechanism, we take advantage of two observations on pathogen-independent immunomodulatory effects from vaccination in the literature: they tend to (1) vary strongly by sex, with beneficial effects from live-attenuated vaccination often seen only in female but not male individuals^6–8^; and (2) depend on the receipt of other vaccines before, or at the same time as, receipt of the vaccine in question^6–8^. Consistent with these observations, we find that the effect of zoster vaccination on new diagnoses of dementia was markedly greater among women than men (Fig. 5 and Supplementary Table 3 (column 1)). There was no significant difference between women and men in the effect of the zoster vaccine on diagnoses of shingles and postherpetic neuralgia (Supplementary Table 3 (columns 2 and 3)). Similarly, the magnitude of the abrupt increase in vaccine uptake at the 2 September 1933 date-of-birth eligibility threshold was comparable between women and men (Supplementary Fig. 24), with a slightly larger magnitude among men.

We also find strong effect heterogeneity by receipt of previous influenza vaccination. Specifically, the protective effect of zoster vaccination for dementia was larger among those who did not recently receive the influenza vaccine (Supplementary Fig. 25). Influenza vaccination is the only vaccine that was provided within the 5 years preceding zoster vaccination eligibility to a substantial proportion of individuals in our study population (pneumococcal vaccination is already provided at age 65 years in the United Kingdom^37^).

Finally, we examined the differences in the effect of the zoster vaccine on dementia incidence between those with versus without an autoimmune or allergic condition. Our reasoning for this analysis was based on the observation that the incidence of shingles is increased among individuals with an autoimmune or allergic condition^38–41^, while there do not appear to be major differences in vaccine immunogenicity and its relative effectiveness for shingles prevention between those with versus without such conditions^30^. Thus, if the protective effect of zoster vaccination for dementia is mainly driven through a reduction of clinical and subclinical virus reactivations, then those with an autoimmune condition will likely benefit equally or more than those without such a condition. However, because autoimmune and allergic conditions are generally characterized by a heightened activation of the (adaptive) immune system^42,43^, individuals with such a condition might benefit less from further activation of more generalized, VZV-independent, immune system pathways than those without such a condition. Consistent with this second hypothesis, we observe suggestive evidence for stronger effectiveness of the zoster vaccine for dementia among those without an autoimmune or allergic condition than those with such a condition (Supplementary Fig. 25). The patterns that we observe remain largely unaffected by whether or not patients were taking any immunosuppressive medications in the year preceding the start of the zoster vaccination program.

Thus, overall and with the caveat that these exploratory analyses are suggestive only, our analyses indicate that both a mechanism of action through a reduction in clinical and subclinical reactivations of VZV as well as through a VZV-independent immunomodulatory effect are plausible. Importantly, these two mechanisms are not mutually exclusive.

## Discussion

Here we found that the zoster vaccine reduced the probability of a new dementia diagnosis by approximately one-fifth over a seven-year follow-up period. By taking advantage of the fact that the unique way in which the zoster vaccine was rolled out in Wales constitutes a natural experiment, and examining each possible remaining source of bias, our study provides evidence that is more likely to be causal in nature than the existing, exclusively associational^15–24^, evidence on this topic. Our substantial effect sizes, combined with the relatively low cost of the zoster vaccine, imply that, if these findings are truly causal, the zoster vaccine will be both far more effective as well as cost-effective in preventing or delaying dementia than existing pharmaceutical interventions.

Our quasi-experimental approach reduces the probability of confounding compared with more standard associational analyses. Moreover, we have provided evidence from a series of analyses against any of the possible remaining sources of bias being a likely explanation of our findings. Nonetheless, it is possible (even if statistically unlikely) that our findings are due to chance. Confirmation of our findings in other populations, settings and data sources is therefore critical. Importantly, we have successfully confirmed our findings using country-wide death certificate data from England and Wales^10^. Specifically, because England rolled out the zoster vaccine in an almost identical way to Wales^44^, we were able to use the same quasi-experimental approach as in our electronic health record data from Wales to determine the effect of eligibility for zoster vaccination based on one’s date of birth on deaths for which the underlying cause was recorded as being dementia. We found that, over a nine-year follow-up period, approximately 1 in 20 such deaths were averted from being eligible for zoster vaccination. This study constitutes an important confirmation of our results because it analysed a different population (England’s population accounts for approximately 95% of England’s and Wales’s combined population^45^), type of data (death certificates as opposed to electronic health records) and outcome (deaths due to dementia). In addition to this confirmation of our results in mortality data, the probability of a chance finding is further reduced by the fact that we successfully replicate our main findings using a second analysis approach (DID-IV) and that our effect sizes remain stable across a multitude of analysis choices, including choice of grace periods, follow-up periods, study population definitions (for example, restriction to frequent healthcare users), functional form of our regressions, width of the week-of-birth window drawn around the date-of-birth eligibility cut-off and index date definitions.

We observed large differences in the effect of zoster vaccination on dementia between women and men, with women benefitting more than men. In our view, these large differences between women and men are plausible for several reasons. First, we cannot exclude the possibility of substantial reductions in new dementia diagnoses from zoster vaccination among men, especially given the lower incidence of dementia in older age among men than women in our data and, therefore, our wider confidence intervals for analyses among men. Second, off-target effects of vaccines have often been observed to be far stronger among female than male individuals, with female individuals benefiting more from live-attenuated vaccines in particular^6,7^. Third, there appear to be important sex differences in the immunological response to vaccines more generally^46^. Lastly, there is a growing body of evidence that there may be differences in the pathogenesis of dementia between women and men^47^.

Other than investing into randomized trials, investments into basic science research on the potential role of VZV and the immune response to the zoster vaccine in the pathogenesis of dementia could provide critical mechanistic insights. There are already several lines of evidence on plausible mechanistic pathways that link VZV reactivations to dementia. Specifically, VZV reactivations have been found to lead to long-lasting cognitive impairment through vasculopathy^48,49^, amyloid deposition and aggregation of tau proteins^50^, neuroinflammation^51–54^, as well as a similar spectrum of cerebrovascular disease as seen in Alzheimer’s disease, including small to large vessel disease, ischaemia, infarction and haemorrhage^51–56^. As suggested by a recent study^57^, it may also be the case that reducing subclinical and clinical reactivations of VZV reduces reactivations of the herpes simplex virus-1 in the brain through neuroinflammatory pathways. This mechanism would link VZV to the body of literature on the role of herpes simplex virus-1 in the pathogenesis of dementia^1–5^. Nonetheless, our exploratory analyses on the effect mechanism that links zoster vaccination to dementia suggest that both a mechanism through reducing clinical and subclinical reactivations of VZV as well as a pathogen-independent immune mechanism are plausible. Some of these possible pathogen-independent immune mechanisms have recently been detailed elsewhere^58^.

Our study has several limitations. First, our outcome ascertainment probably suffers from some degree of under-detection, both in whether and in how timely a fashion dementia is diagnosed. Importantly, because the probability of under-detecting dementia, as well as the delay in doing so, is unlikely to change abruptly at the 2 September 1933 date-of-birth eligibility threshold for zoster vaccination, this outcome misclassification is most likely non-differential. Our effect estimates are therefore likely to be conservative (that is, our absolute effect sizes would be an underestimate of the true absolute effect magnitude). Similarly, changes in the accuracy and timeliness of dementia ascertainment over the years of our follow-period, such as due to changing clinical practice or health system incentives to detect and record dementia, affected those born immediately before versus immediately after 2 September 1933 equally. We would therefore not expect these changes to be a source of bias in our analyses. Second, we are unable to provide estimates for the effectiveness of the zoster vaccine for reducing dementia occurrence in age groups other than those who were weighted most heavily in our regression discontinuity analyses (primarily those aged 79 to 80 years). Third, the COVID-19 pandemic probably affected the timeliness with which dementia was diagnosed. However, the follow-up period used in our primary analyses ended before the start of the COVID-19 pandemic. Moreover, because the pandemic affected those born just before versus just after 2 September 1933 equally, pandemic-related under-detection of dementia is unlikely to bias our relative effect estimates. Fourth, we were limited to a maximum follow-up period of 8 years. Our study can therefore not inform on the effectiveness of the zoster vaccine for reducing dementia occurrence beyond this time period. Lastly, because the newer recombinant subunit zoster vaccine (Shingrix) replaced the live-attenuated zoster vaccine (Zostavax) in the United Kingdom only in September 2023^25^, which is after our follow-up period ended, our effect estimates apply to the live-attenuated zoster vaccine only.

## Methods

### Description of the zoster vaccine rollout in Wales

The live-attenuated zoster vaccine (Zostavax) was made available to eligible individuals in Wales through a staggered rollout system starting on 1 September 2013. Under this system, individuals aged 71 years or older were categorized into three groups on 1 September of each year: (1) an ineligible cohort of those aged 71 to 78 years (or 77 years, depending on the year of the program), who became eligible in the future; (2) a catch-up cohort, consisting of individuals aged 79 years (or 78 years, again depending on the year of the program); and (3) those who were ineligible as they were aged 80 years or older and who never became eligible.

Our analysis focused on adults born between 1 September 1925 (88 years old at program start) and 1 September 1942 (71 years old at program start). Those born between 1 September 1925 and 1 September 1933 never became eligible, whereas those born between 2 September 1933 and 1 September 1942 became progressively eligible in a catch-up cohort. Specifically, the vaccine was offered to those born between 2 September 1933 and 1 September 1934 in the first year of the program (1 September 2013 to 31 August 2014); those born between 2 September 1934 and 1 September 1936 in the second year (1 September 2014 to 31 August 2015); those born between 2 September 1936 and 1 September 1937 in the third year (1 September 2015 to 31 August 2016); and those born between 2 September 1937 and 1 September 1938 in the fourth year (1 September 2016 to 31 August 2017). As of 1 April 2017, individuals become eligible for the vaccine on their 78th birthday and remain eligible until their 80th birthday. Our analysis principally compared individuals born on or shortly after 2 September 1933, to individuals who never became eligible as they were born shortly before 2 September 1933. We show in Supplementary Figs. 26–28 that most eligible individuals, especially in the first two eligibility cohorts, which are the focus of our analysis, took up the vaccination during their first year of eligibility (as opposed to during later years) and that vaccination uptake in these first two eligibility cohorts was of a similar magnitude.

### Data source

Healthcare in Wales is provided through the Welsh National Health Service (NHS), which is part of the United Kingdom’s single-payer single-provider healthcare system^59^. NHS Wales and Swansea University created the SAIL Databank^26,60–64^, which includes full electronic health record data for primary care visits linked to information on hospital-based care as well as the country’s death register data.

SAIL generates a list of all individuals who have ever been registered with a primary care provider in Wales (which is the case for over 98% of adults residing in Wales^27^) from the Welsh Demographic Service Dataset^65^. SAIL then links this universe of individuals to each of the following datasets. Electronic health record data from primary care providers is made available in SAIL through the Welsh Longitudinal General Practice dataset^66^, which contains data from approximately 80% of primary care practices in Wales and 83% of the Welsh population. These electronic health record data use Read codes, which provide detailed information on patients and their care encounters, including diagnoses, clinical signs and observations, symptoms, laboratory tests and results, procedures performed and administrative items^67^. Zoster vaccination was defined using both codes for the administration of the vaccine as well as product codes (Supplementary Table 1). Diagnoses made and procedures performed in the hospital setting (as part of inpatient admissions or day-case procedures) are provided in SAIL through linkage to the Patient Episode Database for Wales^68^, which begins in 1991 and contains data for all hospital-based care in Wales as well as hospital-based care provided in England to Welsh residents. Procedures are encoded using OPCS-4 codes^69^ and diagnoses using ICD-10 codes^70^. Attendance information at any NHS Wales hospital outpatient department is provided through linkage to the Outpatient Database for Wales^71^, which starts in 2004. ICD-10-encoded diagnoses of cancers are identified through linkage to the Welsh Cancer Intelligence and Surveillance Unit^72^, which is the national cancer registry for Wales that records all cancer diagnoses provided to Welsh residents wherever they were diagnosed or treated. This dataset begins in 1994. Finally, cause-of-death data are provided for all Welsh residents (regardless of where they died in the United Kingdom) through linkage to the Annual District Death Extract^73^, which begins in 1996 and includes primary and contributory causes of death from death certificates. Dates for deaths were those on which the death was registered, as opposed to when it occurred. Cause-of-death data use ICD-9 coding until 2001 and ICD-10 coding thereafter.

When testing for any discontinuities in educational attainment across the date-of-birth eligibility threshold, we used a dataset provided by the Office for National Statistics (ONS)^74^. This dataset was generated by the ONS from the 2011 UK Census for all usual residents aged 16 or over, born in Wales between January 1925 and December 1950, regardless of their employment status. The data were categorized by the ONS by sex, month and year of birth (January 1925 to December 1950), highest level of qualification and occupation.

Ethics approval was granted by the Information Governance Review Panel (IGRP, application number, 1306). Composed of government, regulatory and professional agencies, the IGRP oversees and approves applications to use the SAIL databank. All analyses were approved and considered minimal risk by the Stanford University Institutional Review Board on 9 June 2023 (protocol number, 70277).

### Study cohort, follow-up period and loss to follow-up

Our study population consisted of 296,603 individuals born between 1 September 1925 and 1 September 1942 who were registered with a primary care provider in Wales on the start date of the zoster vaccine program rollout (1 September 2013). As we only had access to the date of the Monday of the week in which an individual was born, we were unable to determine whether the individuals born in the cut-off week starting on 28 August 1933 were eligible for the zoster vaccine in the first year of its rollout. We therefore excluded 279 individuals born in this particular week. Among the remaining individuals, 13,783 had a diagnosis of dementia before 1 September 2013 and were therefore excluded from the analyses with new diagnoses of dementia as outcome. The size of our final analysis cohort for all primary analyses for new dementia diagnoses was therefore 282,541. This analysis cohort was used for all analyses except those with incidence of dementia before zoster vaccination program start, shingles and postherpetic neuralgia as outcomes; analyses for which we did not exclude individuals with a dementia diagnosis before 1 September 2013.

We followed individuals from 1 September 2013 to 31 August 2021, which allowed for a maximum follow-up period of 8 years. In our primary specification, we selected a follow-up period of 7 years (that is, until 31 August 2020) because this enabled us to include grace periods of up to 12 months while still keeping the follow-up period constant for individuals on either side of the date-of-birth eligibility cut-off. However, we also show all results for follow-up periods from one to eight years in one-year increments. Owing to the unique anonymized NHS number assigned to each patient, we were able to follow individuals across time even if they changed primary care provider. Patients were therefore only lost to follow-up in our cohort if they emigrated out of Wales or changed to one of the approximately 20% of primary care practices in Wales that did not contribute data to SAIL. Over our seven-year follow-up period, this was the case for 23,049 (8.2%) of adults in our primary analysis cohort, with no significant difference in this proportion between those born just before versus just after the 2 September 1933 eligibility threshold. In total, 92,629 (37.8%) of adults in our primary analysis cohort died during the seven-year follow-up period. Our primary analysis approach does not adjust for any competing risk of death for three reasons. First and foremost, in the absence of a zoster vaccination program, there is no reason that the competing risk of death should differ across the 2 September 1933 date-of-birth eligibility threshold. Second, not adjusting for competing risk of death in our setting is a conservative choice because eligibility for zoster vaccination may reduce (but is very unlikely to increase) all-cause mortality^10,75^. Thus, those eligible for zoster vaccination will, on average, be exposed to a longer time period during which they could become newly diagnosed with dementia. Third, to date, no well-established approach exists for survival analysis in a regression discontinuity framework, including the ability to determine the CACE and optimal bandwidth^76^.

### Definition of outcomes

Owing to the neuropathological overlap between dementia types and difficulty in distinguishing dementia types clinically^77–79^, we chose to define dementia as dementia of any type or cause. Dementia was defined as a diagnosis of dementia made either in primary care (as recorded in the primary care electronic health record data), specialist care or hospital-based care, or dementia being named as a primary or contributory cause of death on the death certificate. The date of the first recording of dementia across any of these data sources was used to define the date on which the patient was diagnosed with dementia. Similarly, all other outcomes were defined using a diagnosis made in any care setting or mentioning as a primary or contributory cause of death. For chronic conditions, the date of the first recording across any of these data sources was used to define the date on which the chronic condition occurred. For non-chronic conditions or events (that is, shingles, postherpetic neuralgia, stroke, lower respiratory tract infections, falls, lower back pain, medication prescriptions, influenza vaccination and breast cancer screening), the date of first recording after the program date across any of these data sources was used for defining the occurrence of the outcome during the follow-up. The Read and ICD-10 codes used to define all outcomes are detailed in the Supplementary Codes.

### Statistical analysis

The two authors who analysed the data (M.E. and M.X.) have coded all parts of the analysis independently. Occasional minor differences, resulting from different data coding choices, were resolved through discussion.

#### Our regression discontinuity approach

We used a regression discontinuity design to analyse our data, which is a well-established method for causal inference in the social sciences^80^. Regression discontinuity analysis estimates expected outcome probabilities just left and just right of the cut-off, to obtain an estimate of the treatment effect. We used local linear triangular kernel regressions (assigning a higher weight to observations lying closer to the date-of-birth eligibility threshold) in our primary analyses and quadratic polynomials in robustness checks. This is the recommended and most robust approach for regression discontinuity analyses even in situations in which the relationship between the assignment variable (here, date of birth) and the outcome is exponential^81^. An important choice in regression discontinuity analyses is the width of the data window (the bandwidth) that is drawn around the threshold. Following standard practice, we used an MSE-optimal bandwidth^82^, which minimizes the MSEs of the regression fit, in our primary analyses. We determined this optimal bandwidth separately for each combination of sample and outcome definition. In robustness checks, we examined the degree to which our point estimates vary across different bandwidth choices ranging from 0.25 times to two times the MSE-optimal bandwidth. We used robust bias-corrected standard errors for inference^83^.

#### Estimating the effect of being eligible for the zoster vaccine

In the first step, we determined the effect of being eligible for the zoster vaccine (regardless of whether the individual actually received the vaccine) on our outcomes. To do so, we estimated the following regression equation:

1 $${Y}_{i}=\alpha +{\beta }_{1}\times {D}_{i}+{\beta }_{2}\times ({{\rm{W}}{\rm{O}}{\rm{B}}}_{i}-{c}_{o})+{\beta }_{3}\times {D}_{i}\times ({{\rm{W}}{\rm{O}}{\rm{B}}}_{i}-{c}_{0})+{{\epsilon }}_{i},$$

where *Y*_*i*_ is a binary variable equal to one if an individual experienced the outcome (for example, shingles or dementia). The binary variable *D*_*i*_ indicates eligibility for the zoster vaccine and is equal to one if an individual was born on or after the cut-off date of 2 September 1933. The term (WOB_*i*_ − *C*_0_) indicates an individual’s week of birth centred around the cut-off date. The interaction term *D*_*i*_ × (WOB_*i*_ − *C*_0_) allows for the slope of the regression line to differ on either side of the threshold. The parameter *β*_1_ identifies the absolute effect of being eligible for the vaccine on the outcome. Wherever we report relative effects, we calculated these by dividing the absolute effect estimate *β*_1_ by the mean outcome just left of the date-of-birth eligibility threshold, that is, the estimate of *α*.

#### Estimating the effect of actually receiving the zoster vaccine

In the second step, we estimated the effect of actually receiving the zoster vaccine on our outcomes. This effect is commonly referred to as the complier average causal effect (CACE) in the econometrics literature^84^. As is standard practice^84^, we used a fuzzy regression discontinuity design to estimate the CACE. Fuzzy regression discontinuity analysis takes into account the fact that the vaccine is not deterministically assigned at the week-of-birth cut-off. Instead, a proportion of ineligible individuals still received the vaccine and a proportion of eligible individuals did not receive the vaccine. To account for this fuzziness in the assignment, the fuzzy regression discontinuity design uses an instrumental variable approach, with the instrumental variable being the binary variable that indicates whether or not an individual was eligible to receive the vaccine, that is, is born on or after 2 September 1933. As we verify in our plot of vaccine receipt by week of birth (Fig. 1a), individuals who were born immediately after the date-of-birth eligibility threshold had a far higher probability of receiving the zoster vaccine compared with those born immediately before the threshold. Other than the abrupt change in the probability of receiving the zoster vaccine, there probably is no other difference in characteristics that affect the probability of our outcomes occurring between those born immediately after versus immediately before the date-of-birth eligibility threshold. Thus, the indicator variable for the date-of-birth eligibility threshold is a valid instrumental variable to identify the causal effect of receipt of the zoster vaccine on our outcomes. To compare the probability of experiencing the outcome between those who actually received the zoster vaccine versus those who did not, the instrumental variable estimation scales the effect size for being eligible for the zoster vaccine by the size of the abrupt change in the probability of receiving the vaccine at the date-of-birth eligibility threshold. The size of the jump is estimated through the following first-stage regression equation:

2 $${V}_{i}=\gamma +{\theta }_{1}\times {D}_{i}+{\theta }_{2}\times ({{\rm{W}}{\rm{O}}{\rm{B}}}_{i}-{c}_{o})+{\theta }_{3}\times {D}_{i}\times ({{\rm{W}}{\rm{O}}{\rm{B}}}_{i}-{c}_{0})+{{\epsilon }}_{i},$$

where *V*_*i*_ is a binary variable indicating whether the individual received the zoster vaccine and *θ*_1_ identifies the discontinuous increase in vaccine receipt at the date-of-birth eligibility threshold. All other parameters are the same as in regression (1).

The CACE estimated by rescaling the effect of eligibility with the first-stage effect from equation (2) can be represented as an IV estimate for *μ*_1_ from the following second-stage regression:

3 $${Y}_{i}=\varphi \,+\,{\mu }_{1}\times {\hat{V}}_{i}+{\mu }_{2}\times ({{\rm{W}}{\rm{O}}{\rm{B}}}_{i}-{c}_{o})+{\mu }_{3}\times {D}_{i}\times ({{\rm{W}}{\rm{O}}{\rm{B}}}_{i}-{c}_{0})+{{\epsilon }}_{i},$$

where ${\hat{V}}_{i}$ is the predicted probability of zoster vaccine receipt obtained from the first-stage estimation from equation (2). This CACE, *μ*_1_, represents the (absolute) average causal effect of receiving the vaccine among compliers, that is, patients who take up the vaccine if and only if they are eligible.

To compute relative effect sizes, we first introduce some notation. Let *R*_0,*c*_ be the mean outcome among unvaccinated compliers and *R*_1,*c*_ the mean outcome among vaccinated compliers just at the threshold. By definition, the absolute CACE is *μ*_1_ = *R*_1,*c*_ − *R*_0,*c*_ and the relative effect is $\frac{{\mu }_{1}}{{R}_{0,c}}$. To estimate the relative effect, we need an estimate for *R*_0,*c*_. While it is impossible to identify compliers individually, we can estimate means of their observable characteristics, including *R*_0,*c*_ (ref. ^85^). Let *R*_1_ denote the mean outcome among all vaccinated individuals (including compliers) at the cut-off. Assuming no defiers exist (patients who get vaccinated if and only if they are not eligible), all vaccinated people are either compliers or always-takers (patients who get vaccinated irrespective of their eligibility). Thus, *R*_1_ is equal to the population-weighted average of the mean outcomes among vaccinated compliers and always-takers: *R*_1_ = *P*_*c*_ × *R*_1__,*c*_ + *P*_*a*_ × *R*_1__,*a*_, where *P*_*c*_ and *P*_*a*_ are the population share of the compliers and always-takers and *R*_1__,*a*_ is the mean outcome among always-takers at the cut-off. Solving for *R*_1__,*c*_ yields ${R}_{1,c}=\frac{{R}_{1}-{R}_{1,a}\times {P}_{a}}{{P}_{c}\,}$. All right-hand-side quantities in this equation can be estimated from data. First, *R*_1_ and *R*_1__,*a*_ can be estimated, respectively, as *α* + *β*_1_ and *α* from re-estimating regression (1) only among vaccinated individuals. Second, *P*_*a*_ and *P*_*c*_ can be estimated, respectively, as $\frac{\gamma }{{\theta }_{1}+\gamma }$ and $\frac{{\theta }_{1}}{{\theta }_{1}+\gamma }$ from regression (2). Finally, we estimate *R*_1__,*c*_ by the above formula and *R*_0,*c*_ = R_1,*c*_ − *μ*_1_. The relative effect is estimated as $\frac{{\mu }_{1}}{{R}_{0,c}}$. All regressions involved in these steps can be stacked and jointly estimated, so that the relative effect is expressed as a differentiable function of known estimators a 95% confidence interval of the relative CACE can be estimated using the delta method^86^.

#### Analyses to investigate whether another intervention used the identical date-of-birth eligibility cut-off

Our analysis can only be confounded if the confounding variable changes abruptly at the 2 September 1933 date-of-birth eligibility threshold such that individuals very close to either side of this threshold would no longer be exchangeable with each other. The most plausible scenario of such a confounding variable would be the existence of an intervention that used the exact same date-of-birth eligibility threshold as the zoster vaccine rollout and that also affected the probability of a dementia diagnosis during our follow-up period. We conducted five analyses to demonstrate that the existence of such an intervention is unlikely, by establishing that measures of outcomes and behaviours that would be affected by such an intervention are smooth across the date-of-birth eligibility cut-off.

First, across a range of birthdates around the 2 September 1933 eligibility threshold, we plotted the probability of having received the following diagnoses or interventions before the start of the zoster vaccine program (on 1 September 2013): diagnosis of shingles, influenza vaccine receipt in the preceding 12 months, receipt of the pneumococcal vaccine as an adult, current statin use (defined as a new or repeat prescription of a statin in the 3 months preceding program start), current use of an antihypertensive medication (defined as a new or repeat prescription of an antihypertensive drug in the 3 months preceding the program start), participation in breast cancer screening (defined as the proportion of women with a record of referral to, attendance at or a report from breast cancer screening or mammography), each of the ten leading causes of disability-adjusted life years and mortality for Wales in 2019 as estimated by the Global Burden of Disease Project^33^, and all comorbidities (except for AIDS, which falls under privacy-protected diagnoses not made available by the SAIL database) that are included in the widely-used Charlson Comorbidity Index^34^. Moreover, we used each of these conditions, gender, decile of Welsh Index of Multiple Deprivation^56^, as well as all input variables to the Dementia Risk Score (as recorded before 1 September 2013)^32^, to predict the probability (while imputing a fixed age) of a new dementia diagnosis for each patient in the MSE-optimal bandwidth in our primary regression discontinuity analysis for dementia. In addition to plotting these predicted probabilities across a range of birthdates around the 2 September 1933 eligibility threshold, we also plotted the distribution of these predicted probabilities for patients who were eligible versus patients who were ineligible for zoster vaccination. The Read codes for each of these variables are provided in Supplementary Tables 1 and 2. As is the case for balance tables in clinical trials, these plots provide reassurance that individuals close to either side of the 2 September 1933 eligibility threshold are likely to be exchangeable with each other.

Second, we conducted the same analysis as we did for individuals with birthdays on either side of the 2 September 1933 threshold also for people with birthdays around 2 September of each of the three years of birth preceding and succeeding 1933. For example, when moving the start date of the program to 1 September 2011, we started the follow-up period on 1 September 2011 and compared individuals around the 2 September 1931 eligibility threshold. To ensure the same length of follow-up in each of these comparisons, we had to reduce the follow-up period to 5 years for this set of analyses. Thus, as an additional check, we shifted the start date of the program to 1 September of each of the six years preceding (but not succeeding) 2013, which enabled us to maintain the same seven-year follow-up period as in our primary analysis. If another intervention that affects dementia risk also used the 2 September threshold to define eligibility, we may then expect to observe effects on dementia incidence for these comparisons of individuals just around the 2 September thresholds of other birth years.

Third, we conducted the identical comparison of individuals around the 2 September 1933 date-of-birth threshold as in our primary analysis, except for starting the follow-up period 7 years before the start of the zoster vaccine program rollout. If there was an intervention that used the 2 September 1933 date-of-birth eligibility threshold but was implemented before the rollout of the zoster vaccine program, then we may expect to see an effect of the September 1933 threshold on dementia incidence in this analysis.

Fourth, we verified that the effects that we observed in our analyses for dementia incidence appear to be specific to dementia. If an intervention that used the exact same date-of-birth eligibility threshold as the zoster vaccine program indeed existed, it would be unlikely to only affect dementia risk without also having an influence on other health outcomes. We therefore conducted the same analysis as for when using dementia incidence as the outcome but for each of the ten leading causes of disability-adjusted life years and mortality in Wales in 2019 for the age group 70+ years^33^, as well as all conditions that are part of the Charlson Comorbidity Index^34^.

Fifth, we tested for discontinuities in educational attainment at the 2 September 1933 date-of-birth threshold using data from the 2011 census in Wales^74^. If an educational policy had used a 2 September (or specifically 2 September 1933) date-of-birth threshold and the policy was effective in increasing educational attainment, we would then expect discontinuities at the 2 September 1933 threshold in the attained education level between eligible and ineligible individuals. We used the identical analysis approach for this balance test as for our primary analyses in the SAIL database, except that we computed ‘honest’ confidence intervals based on the approach by Armstrong and Kolesár because the assignment variable (month of birth) in these data was monthly, and therefore coarser than the assignment variable (week of birth) in our analyses in the SAIL database^87,88^. This approach guards against potential vulnerability to model misspecification and resulting under-coverage of confidence intervals computed with more standard methods. These honest confidence intervals are conservative in the sense that they have good coverage properties irrespective of whether the functional form in the regression discontinuity analysis is misspecified, provided that the true functional form falls within a certain class of functions. For this class, we considered a function class defined by bounds on the second derivative of the conditional expectation function mapping date of birth to the probability attaining a certain educational level. We used conservative bounds of the respective curvatures by relying on global estimation of higher-order polynomials as proposed by Armstrong and Kolesár^88^.

#### Robustness to a different analytical approach

We additionally used a difference-in-differences instrumental variable approach (DID-IV) to confirm the findings from our regression discontinuity design because, in contrast to the regression discontinuity analysis, this approach does not rely on the continuity assumption (that is, the assumption that potential confounding variables do not abruptly change at precisely the date-of-birth eligibility threshold for the zoster vaccine program). To do so, we restricted our sample to patients born between 1 March 1926 and 28 February 1934. This sample consists of 96,767 adults, of whom 7,752 (8.0%) were eligible for, and 3,949 (4.1%) actually received, zoster vaccination. We then divided our sample into yearly cohorts centred around 1 September (that is, a cohort is all patients born between 1 March of one year and 28 February of the following year). Finally, we divided each yearly cohort into a pre-September birth season and a post-September birth season. Using a difference-in-differences approach, we then compared the outcome (new diagnoses of dementia) between patients born in pre- and post-September birth seasons and across yearly cohorts. More precisely, we tested whether the difference in outcomes across birth seasons is different for the 1933/1934 cohort than for the other cohorts. In doing so, we exploit the fact that zoster vaccination eligibility only differs between the two birth seasons in the 1933/1934-cohort but not in other cohorts, while accounting for the possibility that pre-September and post-September birth seasons may be systematically different for other reasons.

Our difference-in-differences setup implies that the interaction between the post-September birth season indicator and the 1933/1934-cohort indicator constitutes an instrumental variable for receipt of the zoster vaccine, enabling us to estimate the CACE (that is, the effect of actually receiving the vaccine among the compliers). This DID-IV approach relies on two important assumptions. As per the standard exclusion restriction assumption of IV analyses, the IV component of our DID-IV approach assumes that vaccine eligibility affects the outcome solely through a change in actual vaccine receipt. The DID component of our DID-IV approach assumes that, in the absence of the vaccine eligibility rule, the between-birth-season difference in vaccine uptake and in dementia incidence would have been the same in the 1933/1934 cohort as in the other cohorts. To investigate the validity of this assumption, we plotted the mean vaccine uptake and dementia incidence with 95% CIs by birth cohorts and birth seasons (Supplementary Fig. 29). As expected, we find that the between-birth-season differences in vaccine uptake diverge only in the 1933/1934 birth cohort. The absence of a between-birth-season difference in other birth cohorts supports the validity of our DID assumption.

To estimate the CACE in this DID-IV framework, we used two-stage least-squares regression. In the first stage, we identify the vaccine uptake due to the exogeneous change in vaccination eligibility by the following regression equation:

4 $${V}_{i}=\theta +\gamma \times {S}_{i}\times {C}_{i}+{\eta }_{m}+{\eta }_{c}+{{\epsilon }}_{i}$$

where *V*_*i*_ is a binary variable indicating patient *i* actually received the zoster vaccine. *S*_*i*_ and *C*_*i*_ are binary variables indicating that patient *i* is born in the post-September birth season and in the 1933/1934 birth cohort, respectively. *γ* identifies the vaccine uptake due to the change in eligibility. *θ*, *η*_*m*_ and *η*_*c*_ are the constant term, birth month (January, February, …, December) and birth cohort (1926/1927, 1927/1928, …, 1933/1934) fixed effect, respectively. *ϵ*_*i*_ is the error term.

In the second stage, we estimate the effect of vaccine receipt by the following regression:

5 $${Y}_{i}=\alpha +{\beta \times \hat{V}}_{i}+{\eta }_{m}+{\eta }_{c}+{{\epsilon }}_{i}$$

where *Y*_*i*_ is the outcome of patient *i*. ${\widehat{V}}_{i}$ is the probability of vaccine receipt predicted from the first-stage regression (4). The coefficient *β* identifies the CACE. *α*, *η*_*m*_ and *η*_*c*_ are the constant term, birth month and birth cohort (1926/1927, 1927/1928, …, 1933/1934) fixed effect, respectively. *ϵ*_*i*_ is the error term.

#### Robustness checks to different analytical specifications

We conducted a series of additional robustness checks. First, instead of starting the follow-up period for all individuals on 1 September 2013, we adjusted the follow-up period to account for the staggered rollout of the program by beginning the follow-up period for each individual on the date on which they first became eligible for the zoster vaccine (as described in the ‘Description of the zoster vaccine rollout in Wales’ section) (Supplementary Fig. 30). We controlled for cohort fixed effects in these analyses to account for the one- to two-year (depending on the year of the program) differences between cohorts in the calendar year in which this moving follow-up window started. That is, we defined one cohort fixed effect for ineligible individuals and the first catch-up cohort and then included additional cohort fixed effects for each group of patients who became eligible at the same time. Second, we varied our definition of a new diagnosis of dementia by implementing our analysis when defining dementia as a new prescription of donepezil hydrochloride, galantamine, rivastigmine or memantine hydrochloride. Third, we tested whether our results for the effect of being eligible for zoster vaccination on new diagnoses of dementia, shingles and postherpetic neuralgia hold across grace periods (that is, time periods since the index date after which follow-up time is considered to begin to allow for the time needed for a full immune response to develop after vaccine administration) of 0, 2, 4, 6, 8, 10 and 12 months (Supplementary Fig. 31). Fourth, we show our results with bandwidth choices of 0.25, 0.50, 0.75, 1.00, 1.25, 1.50, 1.75 and 2.00 times the MSE-optimal bandwidth (Supplementary Fig. 32). Fifth, we verified that our results are similar when using a local second-order polynomial specification instead of local linear regression.

#### Analyses to explore the effect mechanism

##### Changes in healthcare pathways as a result of a shingles episode

We conducted four analyses to examine this potential effect mechanism, the first three of which are described in detail in the main text. The fourth analysis was an event study that focused on the date of a shingles diagnosis during the follow-up period. Our event study compared the mean outcome in each month relative to the month before the date of the shingles diagnosis. Our regression model controls for changes over time (such as due to ageing of the study population or seasonal patterns in healthcare provider visits) using month-level fixed effects.

To implement our event study, we restricted our study population to those 56,098 individuals born within the MSE-optimal bandwidth of our primary regression discontinuity analysis for dementia. We then aggregated our event-level data into monthly longitudinal data, spanning September 2013 to March 2022. For each outcome of interest (as described in the main text), we then estimated the following event-study regression:

6 $$E[{Y}_{it}]={\beta }_{0}\sum _{k\ne -1}{\gamma }_{k}\times {D}_{k}\times {{\rm{shingles}}}_{i}+\,{\eta }_{i}+{\lambda }_{t},$$

where *Y*_*it*_ is the outcome of interest for individual *i* in period *t*; shingles is a binary variable equal to one if the individual was diagnosed with shingles during the follow-up period; *D*_*k*_ are indicator variables for the *k* months before and after the shingles diagnosis (with *k* = −36, −35,…, 35, 36, and set to zero for individuals who were never diagnosed with shingles during the follow-up period); *γ*_*k*_ are the coefficients of interest, which capture the difference in the outcome in month *k* relative to the month before the shingles diagnosis; *η*_*i*_ is an individual-level fixed effect capturing time-invariant differences across individuals; and *λ*_*t*_ is a month-level fixed effect, capturing differences across periods. We used standard errors that allowed for clustering at the individual level, and therefore for autocorrelation.

##### Reduction in reactivations of the varicella zoster virus

We conducted four analyses to examine this potential effect mechanism. First, we implemented the identical regression discontinuity as in our primary analysis, except that we included a binary variable for being diagnosed with shingles during the follow-up period as a covariate. For the resulting estimate to be an unbiased measure of the degree to which the effect of zoster vaccination on dementia incidence is mediated by shingles diagnoses, there must be no variables that are related to both new dementia diagnoses and the probability of being diagnosed with shingles (that is, no confounding of the mediator-to-outcome relationship)^89^.

Second, to examine when during the follow-up period the dementia-delaying or dementia-preventing effect of zoster vaccination begins to emerge, we plotted Kaplan–Meier plots and (to account for competing risks) cumulative incidence curves among individuals born close to 2 September 1933. This analysis was based on the concept of local randomization^28,29^, which relies on exchangeability of individuals born immediately before versus immediately after 2 September 1933. To define the bandwidth for our analysis in which we could reasonably assume exchangeability across the threshold while maximizing statistical power, we used the widest bandwidth for which we achieved balance in baseline demographic and clinical characteristics of individuals eligible versus ineligible for zoster vaccination. We evaluated bandwidths ranging from 100% to 10% of the MSE-optimal bandwidth (90.6 weeks) in our primary regression discontinuity analysis in 10% decrements. The variables we used for our balance tests were the 14 variables listed in Supplementary Figs. 1 and 2 (except for the more sex-specific variables of past breast cancer screening, breast cancer and prostate cancer diagnoses) using a significance threshold of *P* < 0.05, while controlling for the false-discovery rate using the Benjamini–Hochberg procedure^90^. The largest bandwidth that achieved balance across all variables was 54.4 weeks.

Third, to investigate whether antiviral treatment during a shingles episode was associated with a reduction in the risk of dementia relative to not receiving treatment during a shingles episode, we restricted our study population to those individuals who received a diagnosis of shingles at any time after 1 January 2000 and had not received a diagnosis of dementia before 1 January 2000. Our exposure of interest in this analysis was whether or not an individual received a prescription of antiviral medication (acyclovir, famcyclovir, valacyclovir or inosine pranobex) within three months of the first shingles diagnosis. Individuals were followed up from the date of first shingles diagnosis until either the date of death, moving out of Wales, GP deregistration or end of data availability (1 March 2022). We then used a multivariable Cox proportional hazards model to regress diagnoses of dementia made after the date of the first recorded shingles episode onto whether or not the patient received an antiviral medication prescription for the first shingles episode. In a robustness check, we required that a new diagnosis of dementia must have been made at least 12 months after the date of the first shingles diagnosis. We adjusted our regressions for gender, restricted cubic splines (with three knots) of age at the first shingles infection, and the 12 variables in Supplementary Fig. 1 (excluding past breast and prostate cancer diagnoses).

Fourth, to explore whether experiencing recurrent shingles episodes was associated with a higher risk of dementia than having only a single episode, we used the same study population as in our analysis for treated versus untreated shingles. We matched individuals (via 1:1 propensity score matching) who had more than one shingles diagnosis (with the diagnosis dates having to be at least three months apart) after 1 January 2000 to individuals who only received a single shingles diagnosis after 1 January 2000. We matched individuals on proximity in the date of their first shingles diagnosis as well as the same list of baseline variables as for our analysis of treated versus untreated shingles, and forced an exact match on week of birth and gender. In each matched pair, we used the date of the second shingles diagnosis of the individual with more than one shingles diagnosis as the start date of the follow-up period. Using a Cox proportional hazards model, we then regressed new diagnoses of dementia made during the follow-up period onto whether or not the individual had received more than one shingles diagnosis. In a robustness check, we again required that a new diagnosis of dementia must have been made at least 12 months after the start date of the follow-up period.

##### VZV-independent immunomodulatory effect

To estimate the treatment effect heterogeneities described under this section in the main text, we fully interacted our fuzzy regression discontinuity model with a binary variable that indicates having the condition in question (for example, an autoimmune condition). Precisely, the fully interacted model was specified as:

7 $$\begin{array}{c}{Y}_{i}=\alpha +{\beta }_{1}\times {V}_{i}+{\beta }_{2}\times ({{\rm{W}}{\rm{O}}{\rm{B}}}_{i}-{c}_{0})+{\beta }_{3}\times {D}_{i}\times ({{\rm{W}}{\rm{O}}{\rm{B}}}_{i}-{c}_{0})\\ \,+{\beta }_{4}\times {V}_{i}\times {{\rm{H}}{\rm{E}}{\rm{T}}}_{i}+{\beta }_{5}\times ({{\rm{W}}{\rm{O}}{\rm{B}}}_{i}-{c}_{0})\times {{\rm{H}}{\rm{E}}{\rm{T}}}_{i}\\ \,+{\beta }_{6}\times {D}_{i}\times ({{\rm{W}}{\rm{O}}{\rm{B}}}_{i}-{c}_{0})\times {{\rm{H}}{\rm{E}}{\rm{T}}}_{i}+{\beta }_{7}\times {{\rm{H}}{\rm{E}}{\rm{T}}}_{i}+{{\epsilon }}_{i}\end{array}$$

where the subscript *i* indexes individuals. *Y*_*i*_ is a binary variable equal to 1 if an individual was newly diagnosed with dementia during the follow-up period. The binary variable *V*_*i*_ indicates receipt of the zoster vaccine. The binary variable *D*_*i*_ indicates eligibility for the zoster vaccine (that is, born on or after 2 September 1933). The term WOB_*i*_ − *c*_0_ indicates an individual’s week of birth centred around the date-of-birth eligibility threshold. The interaction term *D*_*i*_ × (WOB_*i*_ − *c*_0_) allows for the slope of the regression line to differ on either side of the date-of-birth eligibility threshold. The binary variable HET_*i*_ is equal to one if an individual had the condition in question. Adding the terms (WOB_*i*_ − *c*_0_) × HET_*i*_ and *D*_*i*_ × (WOB_*i*_ − *c*_0_) × HET_*i*_ allows the slopes to vary by this condition.

*V*_*i*_ and *V*_*i*_ × HET_*i*_ are instrumented by *D*_*i*_ and *D*_*i*_ × HET_*i*_. Using the two-stage least-squares approach, the parameter *β*_4_ identifies the effect heterogeneity, that is, the difference in CACE on the outcome between patients with and without the condition. *β*_1_ and *β*_1_ + *β*_4_ identify the effect among compliers in the reference and comparison group, respectively. The estimates of the effects and heterogeneity are reported in absolute terms. To be consistent with our primary fuzzy regression discontinuity model (that is, without the HET_*i*_ and interaction terms), we used local linear triangular kernel regressions and the MSE-optimal bandwidth from the primary model of the respective outcome.

For our analyses for autoimmune and allergic conditions, we used the 19 most common autoimmune conditions as defined previously^91^, and grouped the 11 least common conditions among them into a rare conditions category. We judged those conditions to be rare that had an incidence of less than 1% during the follow-up period in our cohort. These rare conditions included Addison’s disease, ankylosing spondylitis, Coeliac disease, Hashimoto’s thyroiditis, multiple sclerosis, myasthenia gravis, primary biliary cirrhosis, Sjögren’s syndrome, systemic lupus erythematosus, systemic sclerosis and vitiligo. For common allergic conditions, we used those defined previously^92^.

### Reporting summary

Further information on research design is available in the Nature Portfolio Reporting Summary linked to this article.

## Online content

Any methods, additional references, Nature Portfolio reporting summaries, source data, extended data, supplementary information, acknowledgements, peer review information; details of author contributions and competing interests; and statements of data and code availability are available at 10.1038/s41586-025-08800-x.

## Supplementary information

Supplementary Information Supplementary Figs. 1–32 and Supplementary Tables 1–3.

Reporting Summary

Supplementary Codes All Read and ICD-10 codes used throughout the analysis.

## Data Availability

The data supporting the findings of this study are available from the SAIL Databank^26^. Researchers must request access to the data directly from SAIL. The authors have no permission to share the data. This paragraph describes how access to the data in the SAIL Databank can be obtained. All proposals to use SAIL data are subject to review by an independent Information Governance Review Panel (IGRP). Before any data can be accessed, approval must be given by the IGRP. The IGRP carefully considers each project to ensure the proper and appropriate use of SAIL data. When access has been granted, it is gained through a privacy-protecting trusted research environment (TRE) and remote access system referred to as the SAIL Gateway. SAIL has established an application process, which includes the payment of a fee, to be followed by anyone who would like to access data through SAIL at https://saildatabank.com/ data/apply-to-work-with-the-data/. Once approved, researchers will have to sign a data access agreement and request a gateway account. After the account is approved, researchers will be able to log into the secure SAIL Gateway remotely. Once logged in, researchers can import our SQL/R code and run the analyses by downloading our replication package (https://osf.io/cfnr6/?view_only=d3774e4fda2649e2b2031431b1234874), uploading the package (SQL and R scripts) to the SAIL Gateway through the secure file upload process, and executing the scripts in the Gateway environment.

## Code availability

All Read and ICD-10 codes to define variables are available in the Supplementary Codes. All statistical packages including version numbers for version control, algorithms to define variables and R analysis code are provided in an OSF repository (https://osf.io/cfnr6/?view_only=d3774e4fda2649e2b2031431b1234874)^93^.
