# Supplementary material for: A natural experiment on the effect of herpes zoster vaccination on dementia
Source: Nature. 2025 Apr 2;641(8062):438–46. doi: 10.1038/s41586-025-08800-x (PMC12058522; doi:10.1038/s41586-025-08800-x)
Supplement: Supplementary file 1 — Supplementary Figs. 1–32 and Supplementary Tables 1–3. [file 41586_2025_8800_MOESM1_ESM.docx]

**A natural experiment on the effect of herpes zoster vaccination on dementia**

**Supplementary Information A**

Correspondence to: pgeldsetzer@stanford.edu

This PDF file includes:

- Figs. 1 to 32
- Tables 1 to 3


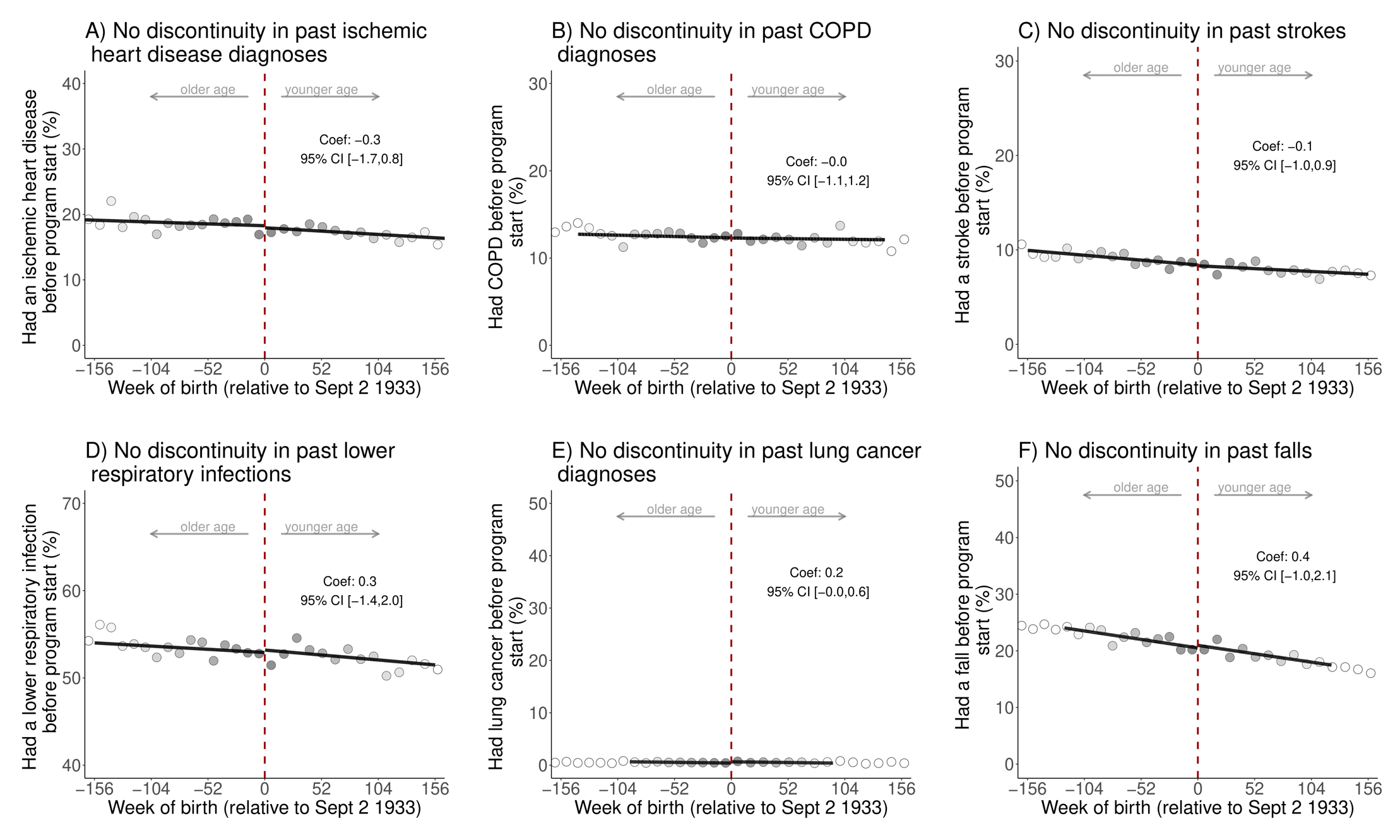
**Figs. 1 to 32**


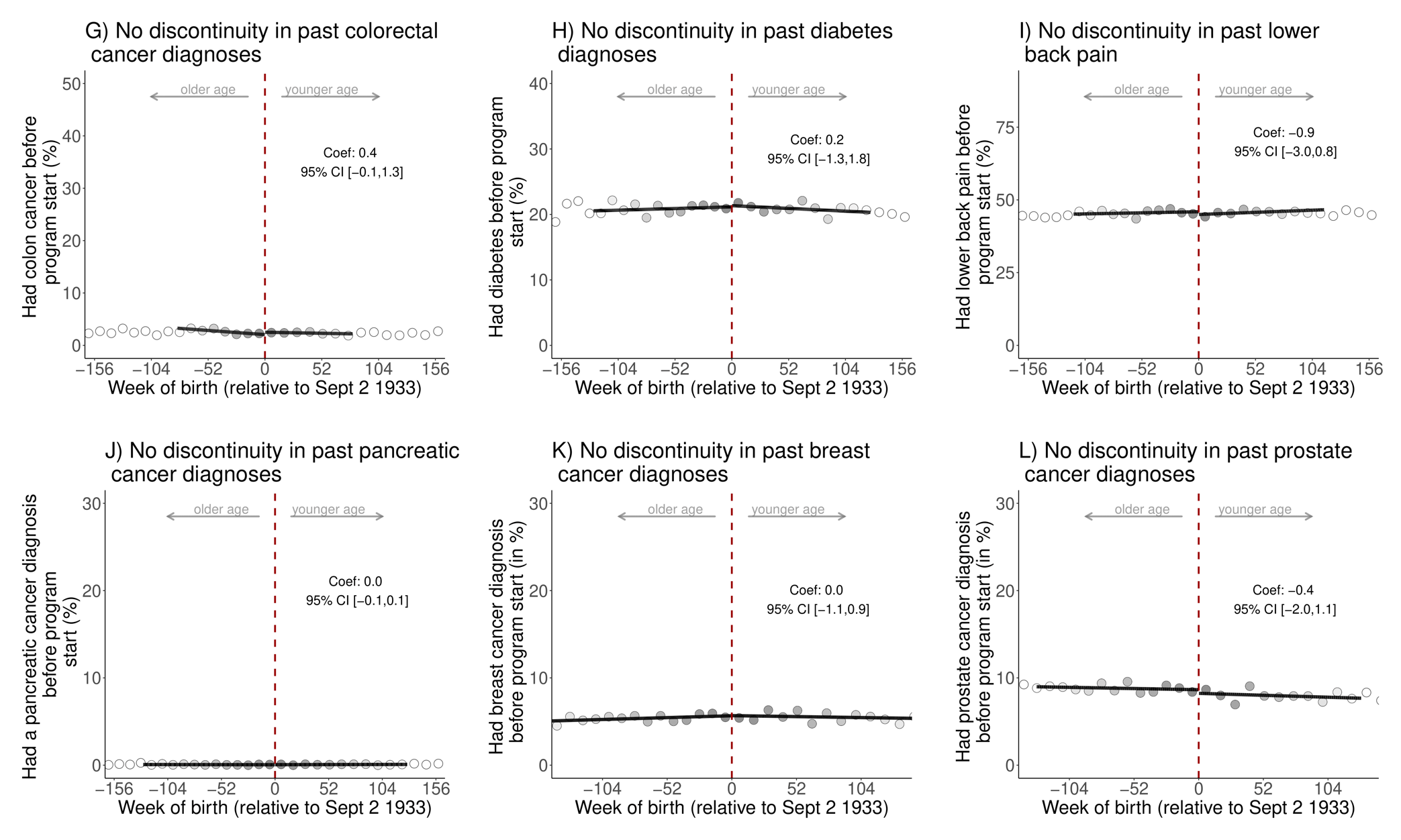


**Fig. 1**: There is exchangeability at baseline across the date-of-birth eligibility cutoff for the ten (other than dementia) leading causes of disability-adjusted life years and mortality in Wales in 2019.^1,2,3,4,5,6,7^

^1^ The data source for this analysis was the SAIL database for Wales.

^2^ Grey dots show the mean value for each 10-week increment in week of birth.

^3^ The grey shading of the dots is in proportion to the weight that observations from this 10-week increment received in the analysis.

^4^ All analyses were run on the same sample as those for the effect of the zoster vaccine on dementia occurrence (n=282,541 adults).

^5^ The analysis of breast cancer diagnoses was restricted to women only (n=154,218 women); the analysis of prostate cancer diagnoses was restricted to men only (n=128,322 men).

^6^ P-values based on two-sided t-tests and unadjusted for multiple hypothesis testing: A) ischemic heart disease: 0.477, B) COPD: 0.931, C) stroke: 0.873, D) lower respiratory infections: 0.687, E) lung cancer: 0.075, F) falls: 0.495, G) colorectal cancer: 0.114, H) diabetes: 0.761, I) lower back pain: 0.269, J) pancreatic cancer: 0.981, K) breast cancer: 0.871, L) prostate cancer: 0.542.

^7^ When adjusting for multiple hypothesis testing (using Anderson’s sharpened q-values) jointly for all baseline conditions and preventive health measures shown in Extended Data Fig. 1 to 3, all p-values were equal to 1.

Abbreviations: Coef=coefficient; CI=confidence interval; COPD=chronic obstructive pulmonary disease.


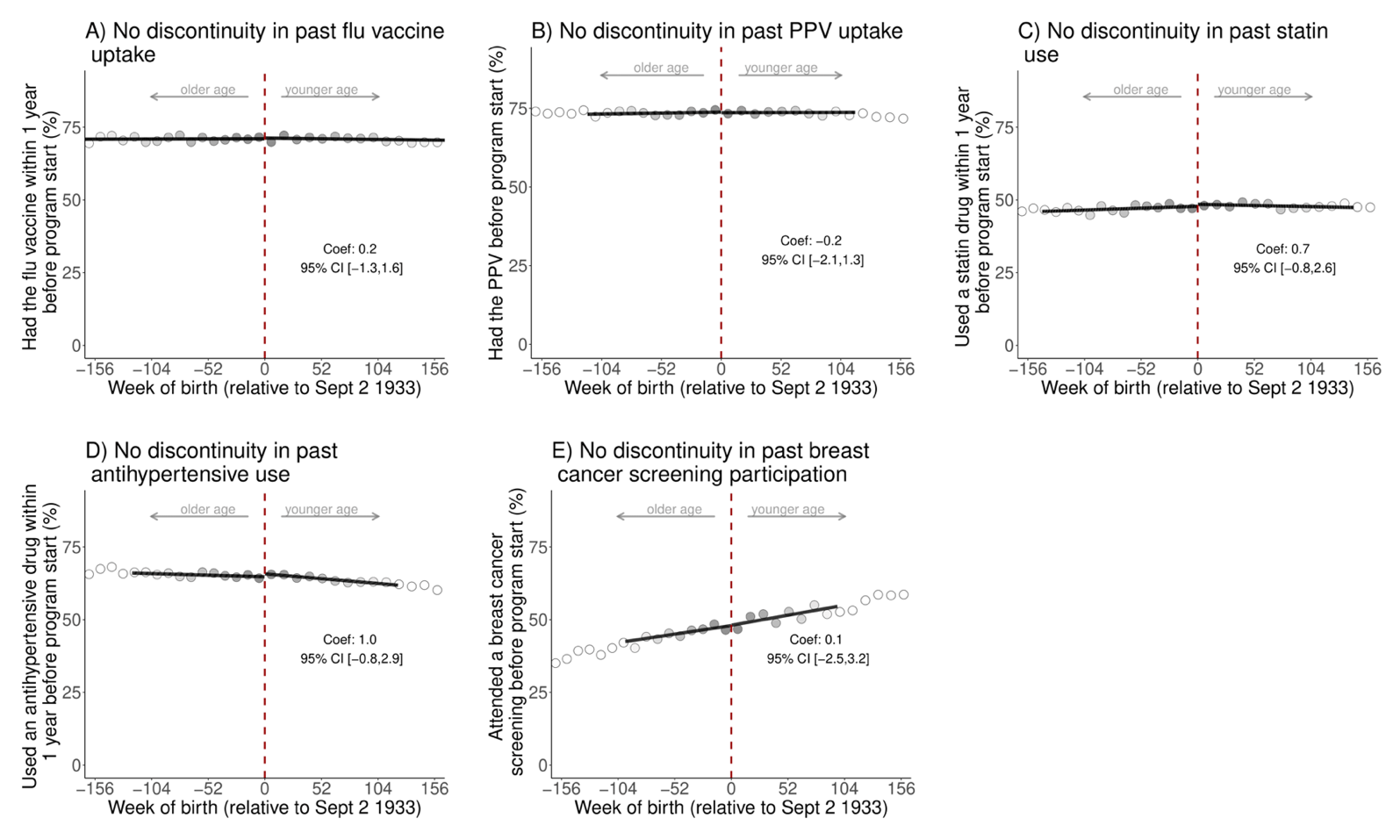


**Fig. 2**: No abrupt change at the date-of-birth eligibility cutoff in the probability of having taken up preventive health measures before the start date of the zoster vaccine program.^1,2,3,4,5,6,7,8^

^1^ The data source for this analysis was the SAIL database for Wales.

^2^ Panels A and B are also shown in Fig. 1 in the main manuscript. They have been repeated here for comprehensiveness as flu vaccine uptake and PPV uptake also constitute preventive health measures.

^3^ All analyses were run on the same sample as those for the effect of the zoster vaccine on dementia occurrence (n=282,541 adults).

^4^ Breast cancer screening participation was defined as having a record of referral to, attendance at, or a report from “breast cancer screening” or mammography. The analysis of breast cancer screening participation was restricted to women only (n=154,218 women).

^5^ Grey dots show the mean value for each 10-week increment in week of birth.

^6^ The grey shading of the dots is in proportion to the weight that observations from this 10-week increment received in the analysis.

^7^ P-values based on two-sided t-tests unadjusted for multiple hypothesis testing: A) flu vaccine: 0.802, B) PPV: 0.638, C) statins: 0.302, D) antihypertensive medications: 0.258, E) breast cancer screening: 0.787.

^8^ When adjusting for multiple hypothesis testing (using Anderson’s sharpened q-values) jointly for all baseline conditions and preventive health measures shown in Extended Data Fig. 1 to 3, all p-values were equal to 1.

Abbreviations: PPV=pneumococcal polysaccharide vaccine; Coef=coefficient; CI=95% confidence interval; Sept=September.

*
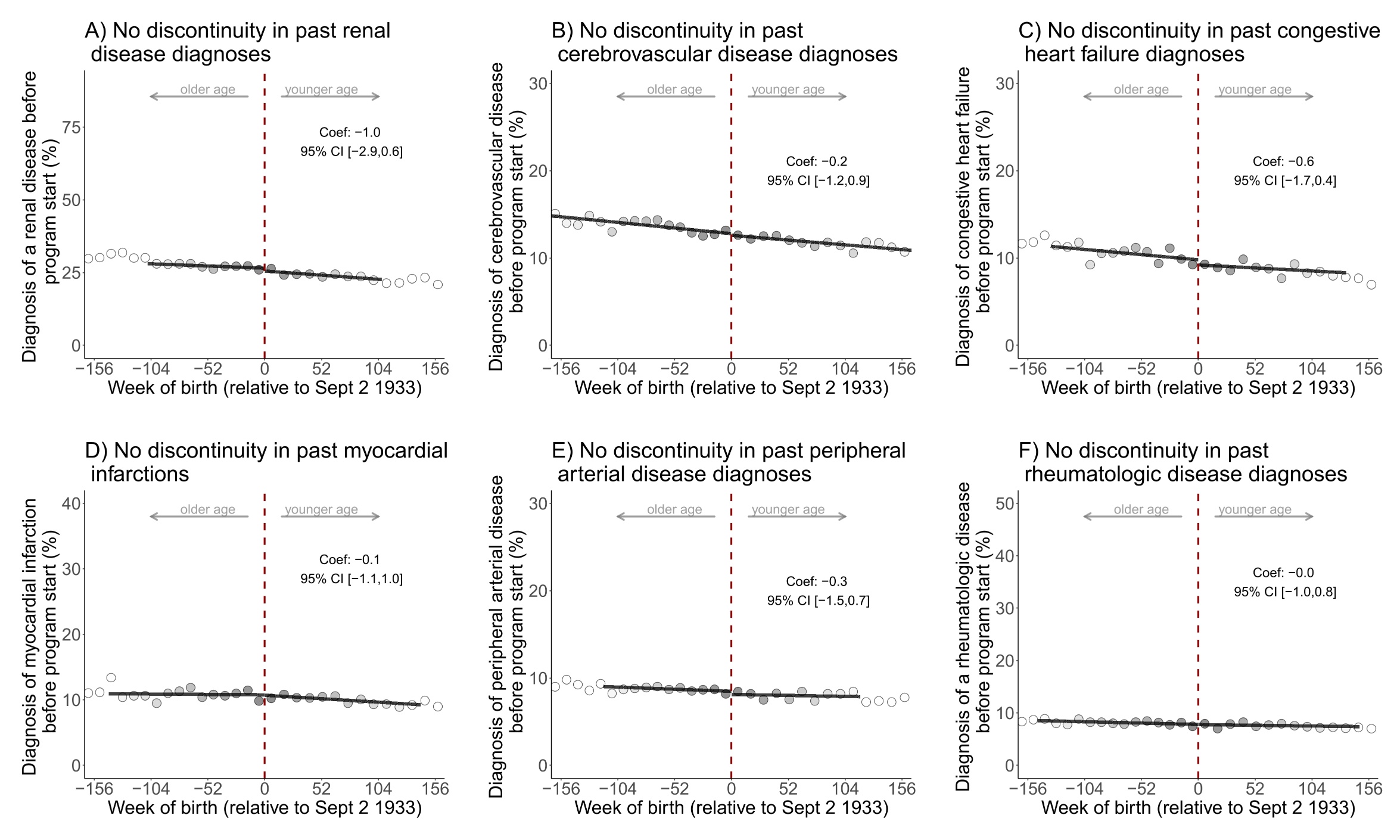
*

*
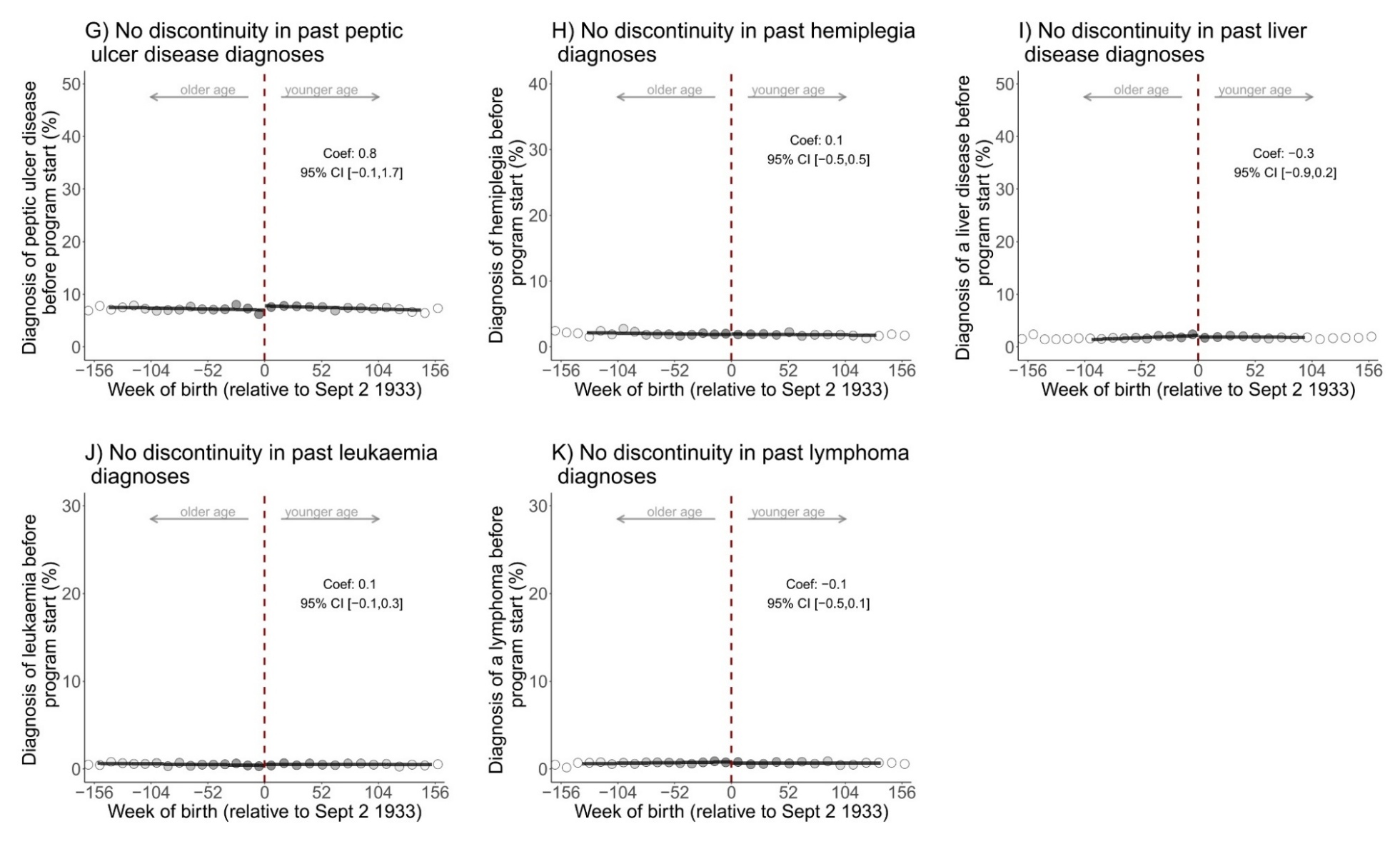
*

**Fig. 3**: There is exchangeability at baseline across the date-of-birth eligibility cutoff for conditions in the Charlson Comorbidity Index.^1,2,3,4,5,6,7^

^1^ This figure only shows those conditions that are not already shown in Extended Data Fig. 1.

^2^ The data source for this analysis was the SAIL database for Wales.

^3^ All analyses were run on the same sample as those for the effect of the zoster vaccine on dementia occurrence (n=282,541 adults).

^4^ Grey dots show the mean value for each 10-week increment in week of birth.

^5^ The grey shading of the dots is in proportion to the weight that observations from this 10-week increment received in the analysis.

^6^ P-values based on two-sided t-tests unadjusted for multiple hypothesis testing: A) renal disease: 0.206, B) cerebrovascular disease: 0.777, C) congestive heart failure: 0.220, D) myocardial infarction: 0.945, E) peripheral arterial disease: 0.492, F) rheumatologic disease: 0.826, G) peptic ulcer disease: 0.092, H) hemiplegia: 0.865, I) liver disease: 0.227, J) leukaemia: 0.342, K) lymphoma: 0.313.

^7^ When adjusting for multiple hypothesis testing (using Anderson’s sharpened q-values) jointly for all baseline conditions and preventive health measures shown in figures 1 to 3, all p-values were equal to 1.

Abbreviations: Coef=coefficient; CI=95% confidence interval; Sept=September.


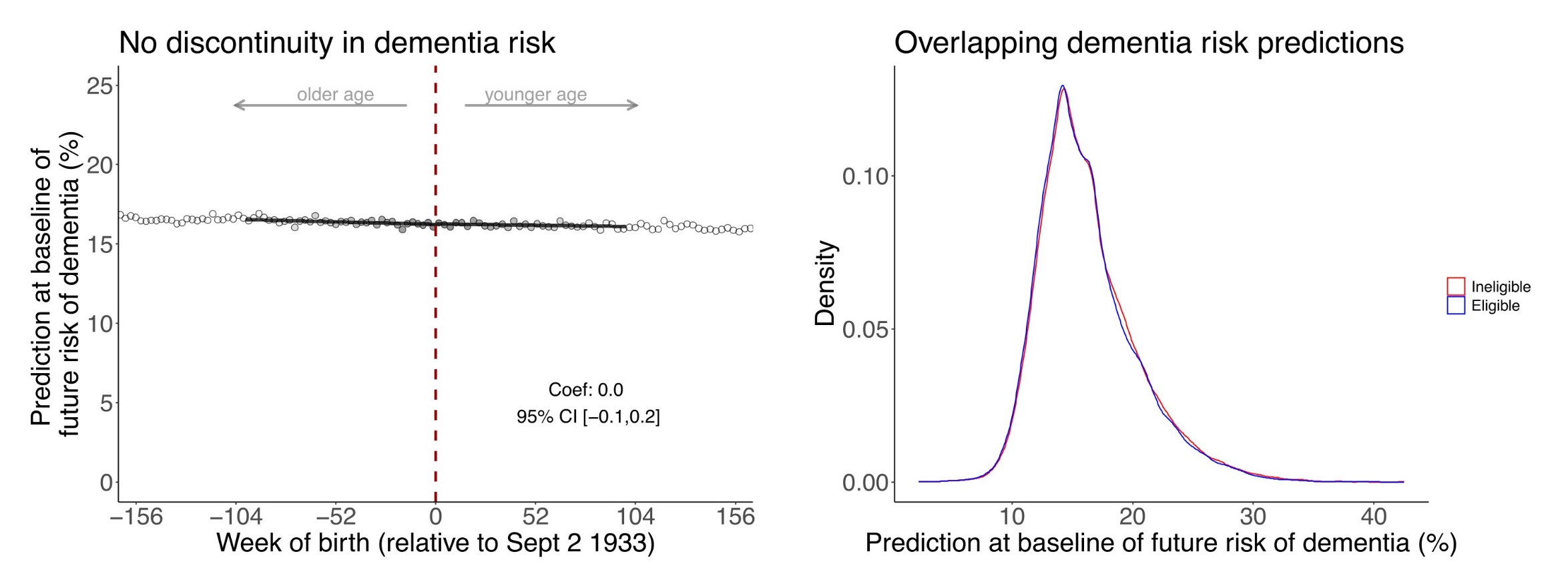


**Fig. 4**: There is exchangeability at baseline across the date-of-birth eligibility cutoff for predicted dementia risk.^1,2,3,4,5^

^1^ The data source for this analysis was the SAIL database for Wales.

^2^ Grey dots show the mean value for each week of birth.

^3^ This analysis was run on the same sample as those for the effect of the zoster vaccine on dementia occurrence (n=282,541 adults).

^4^ The grey shading of the dots is in proportion to the weight that observations from this weekly increment received in the analysis.

^5^ Dementia risk was predicted by using all conditions in Extended Data Fig. 1 to 3, as well as gender, decile of the Welsh Index of Multiple Deprivation (WIMD), and all input variables to the Dementia Risk Score.

Abbreviations: Coef=coefficient; CI=95% confidence interval; Sept=September.


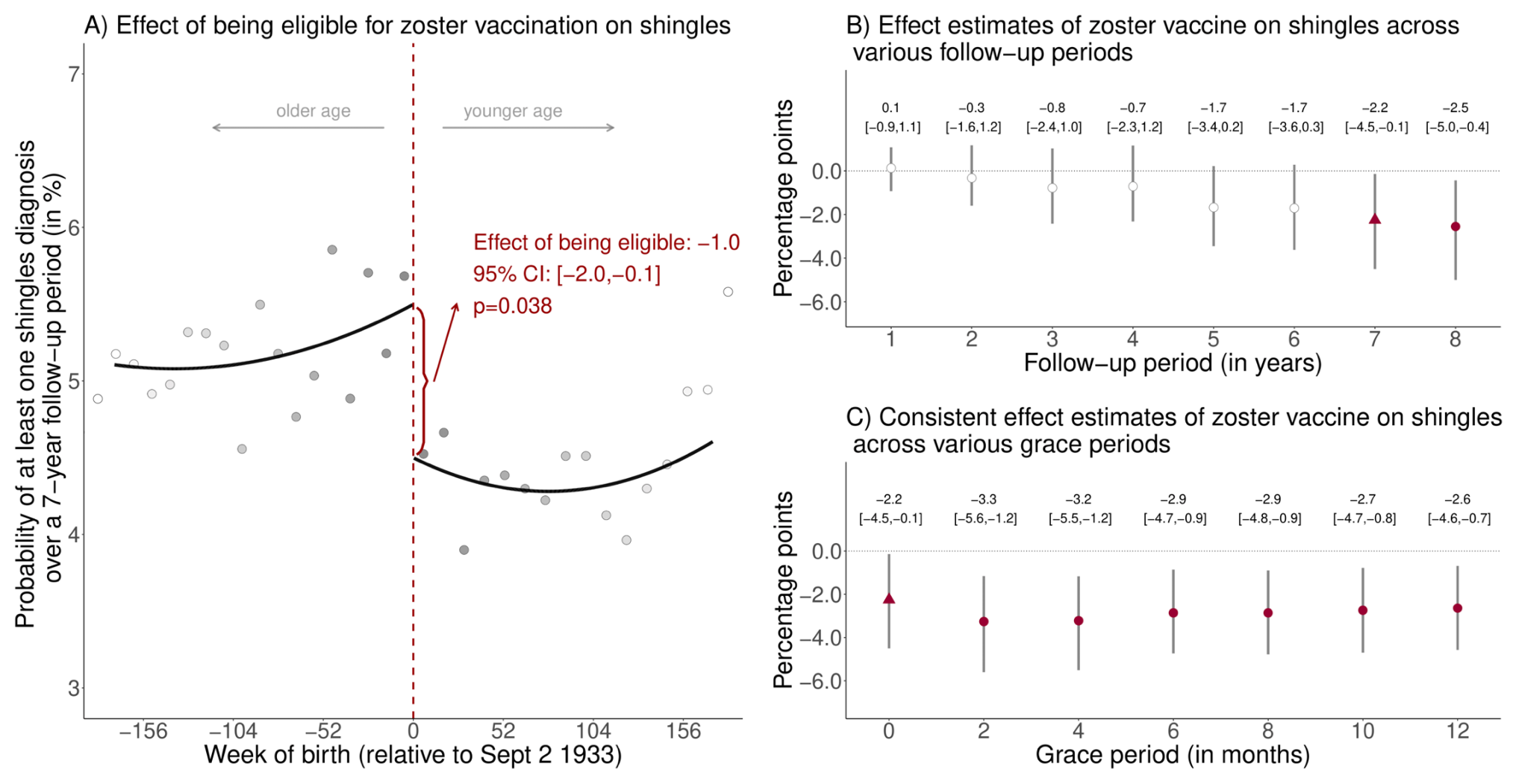


**Fig. 5**: Effect estimates of being eligible (A) and having received the zoster vaccine (B and C) on having at least one shingles diagnosis using local squared regression instead of local linear regression.^1,2,3,4,5,6,7,8^

^1^ The data source for this analysis was the SAIL database for Wales

^2^ Triangles (rather than points) depict our primary specification.

^3^ Red (as opposed to white) fillings denote statistical significance (p<0.05).
^4^ With “grace periods” we refer to time periods since the index date after which follow-up time is considered to begin to allow for the time needed for a full immune response to develop after vaccine administration.
^5^ Grey vertical bars depict 95% confidence intervals around the point estimate of the coefficients (two-sided t-tests).

^6^ Grey dots show the mean value for each 10-week increment in week of birth.

^7^ The grey shading of the dots is in proportion to the weight that observations from this 10-week increment received in the analysis.

^8^ For panel A, the mean squared error-optimal bandwidth is 173.0 weeks (113,030 adults). For panel B and C, in our primary specification the mean squared error-optimal bandwidth is 153.5 weeks (100,527 adults).

Abbreviations: Coef=coefficient; CI=95% confidence interval; Sept=September.

**Fig. 6**: Point estimates for the effect of the zoster vaccine on at least one shingles diagnosis and new dementia diagnoses across different bandwidth specifications.^1,2,3,4,5,6^

^1^ The data source for this analysis was the SAIL database for Wales.

^2^ The bandwidth is the window (in weeks) in participants’ date of birth that is drawn around the September 2 1933 eligibility threshold. The shorter the bandwidth is, the wider is the 95% confidence interval.

^3^ 95% confidence intervals are not symmetrical around the point estimate because we used robust bias-corrected confidence intervals.

^4^ The MSE-optimal bandwidth is 90.6 weeks (n=56,098 adults) and 116.9 weeks (n=76,316 adults) on either side of the threshold for the dementia and shingles outcome, respectively.

^5^ Grey vertical bars depict 95% confidence intervals around the point estimates of the coefficients (two-sided t-tests).

^6^ White points depict statistically insignificant point estimates (p>0.05).

Abbreviations: MSE=mean squared error.


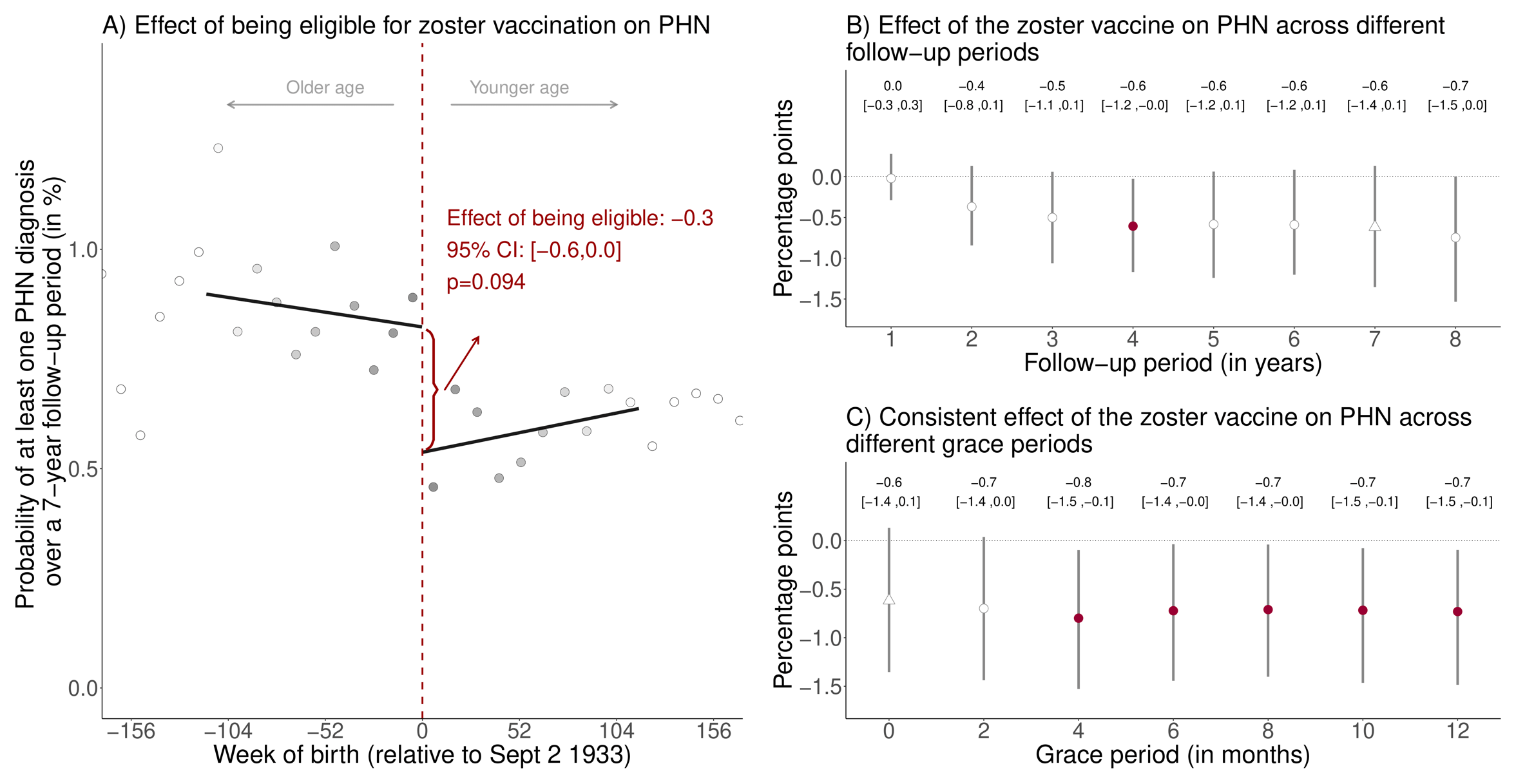

**Fig. 7**: Effect estimates of being eligible (A) and having received the zoster vaccine (B and C) on having at least one diagnosis of postherpetic neuralgia (PHN) during the follow-up period.^1,2,3,4,5,6,7,8^

^1^ The data source for this analysis was the SAIL database for Wales.

^2^ Triangles (rather than points) depict our primary specification.

^3^ Red (as opposed to white) fillings denote statistical significance (p<0.05).
^4^ With “grace periods” we refer to time periods since the index date after which follow-up time is considered to begin to allow for the time needed for a full immune response to develop after vaccine administration.
^5^ Grey vertical bars depict 95% confidence intervals around the point estimates of the coefficients (two-sided t-tests).

^6^ Grey dots show the mean value for each 10-week increment in week of birth.

^7^ The grey shading of the dots is in proportion to the weight that observations from this 10-week increment received in the analysis.

^8^ For panel A, the mean squared error-optimal bandwidth is 116.0 weeks (75,664 adults) and the p-value is calculated based on a two-sided t-test. For panel B and C, in our primary specification the mean squared error-optimal bandwidth is 96.6 weeks (63,039 adults).

Abbreviations: PHN=postherpetic neuralgia; Coef=coefficient; CI=95% confidence interval; Sept=September.

**
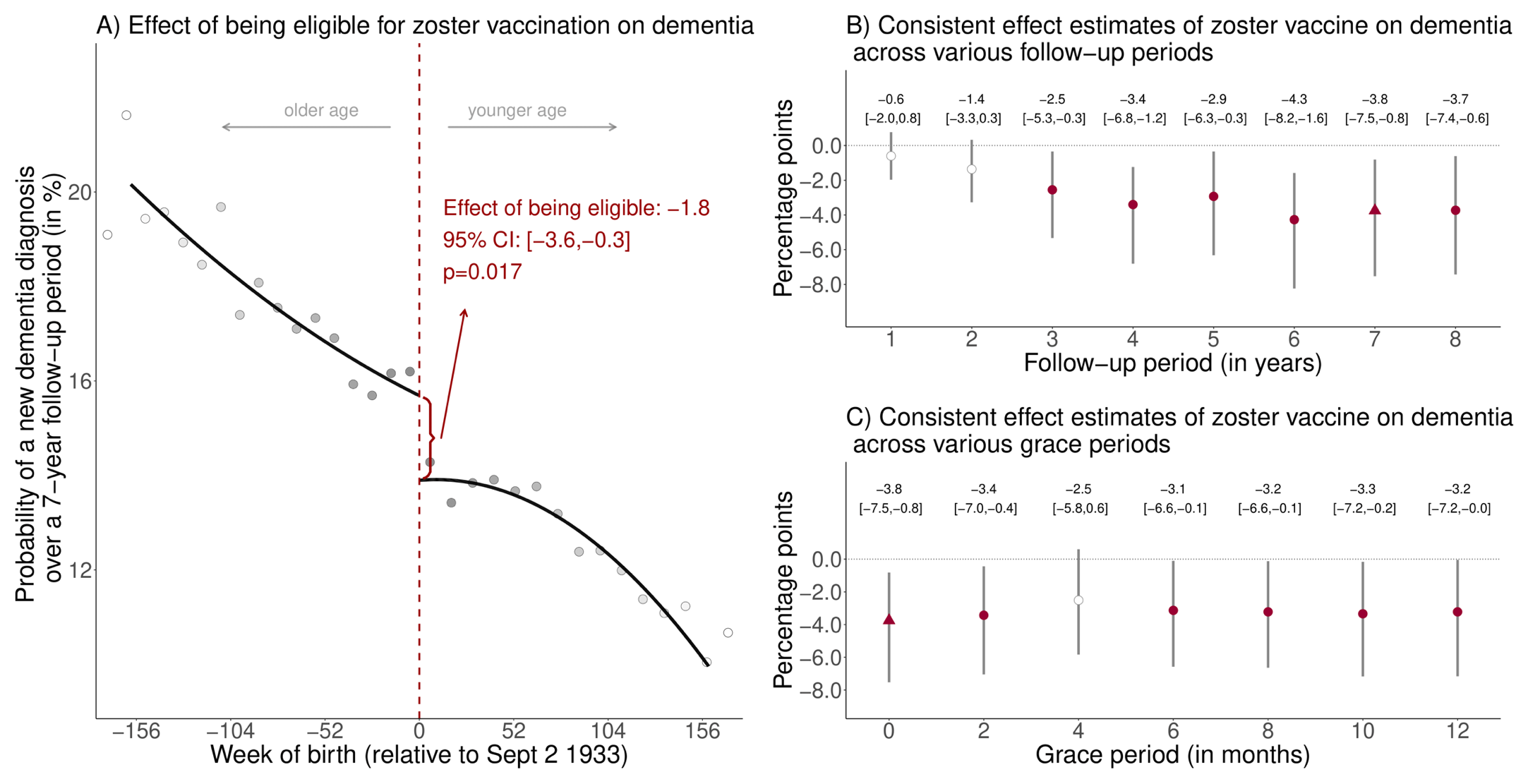
**

**Fig. 8**: Effect estimates of being eligible (A) and having received the zoster vaccine (B and C) on new diagnoses of dementia using local squared regression instead of local linear regression.^1,2,3,4,5,6,7,8^

^1^ The data source for this analysis was the SAIL database for Wales.

^2^ Triangles (rather than points) depict our primary specification.

^3^ Red (as opposed to white) fillings denote statistical significance (p<0.05).
^4^ With “grace periods” we refer to time periods since the index date after which follow-up time is considered to begin to allow for the time needed for a full immune response to develop after vaccine administration.
^5^ Grey vertical bars depict 95% confidence intervals around the point estimates of the coefficients (two-sided t-tests).

^6^ Grey dots show the mean value for each 10-week increment in week of birth.

^7^ The grey shading of the dots is in proportion to the weight that observations from this 10-week increment received in the analysis.

^8^ For panel A, the mean squared error-optimal bandwidth is 159.4 weeks (98,939 adults) and the p-value is calculated based on a two-sided t-test. For panel B and C, in our primary specification the mean squared error-optimal bandwidth is 170.1 weeks (105,232 adults).

Abbreviations: Coef=coefficient; CI=95% confidence interval; Sept=September.


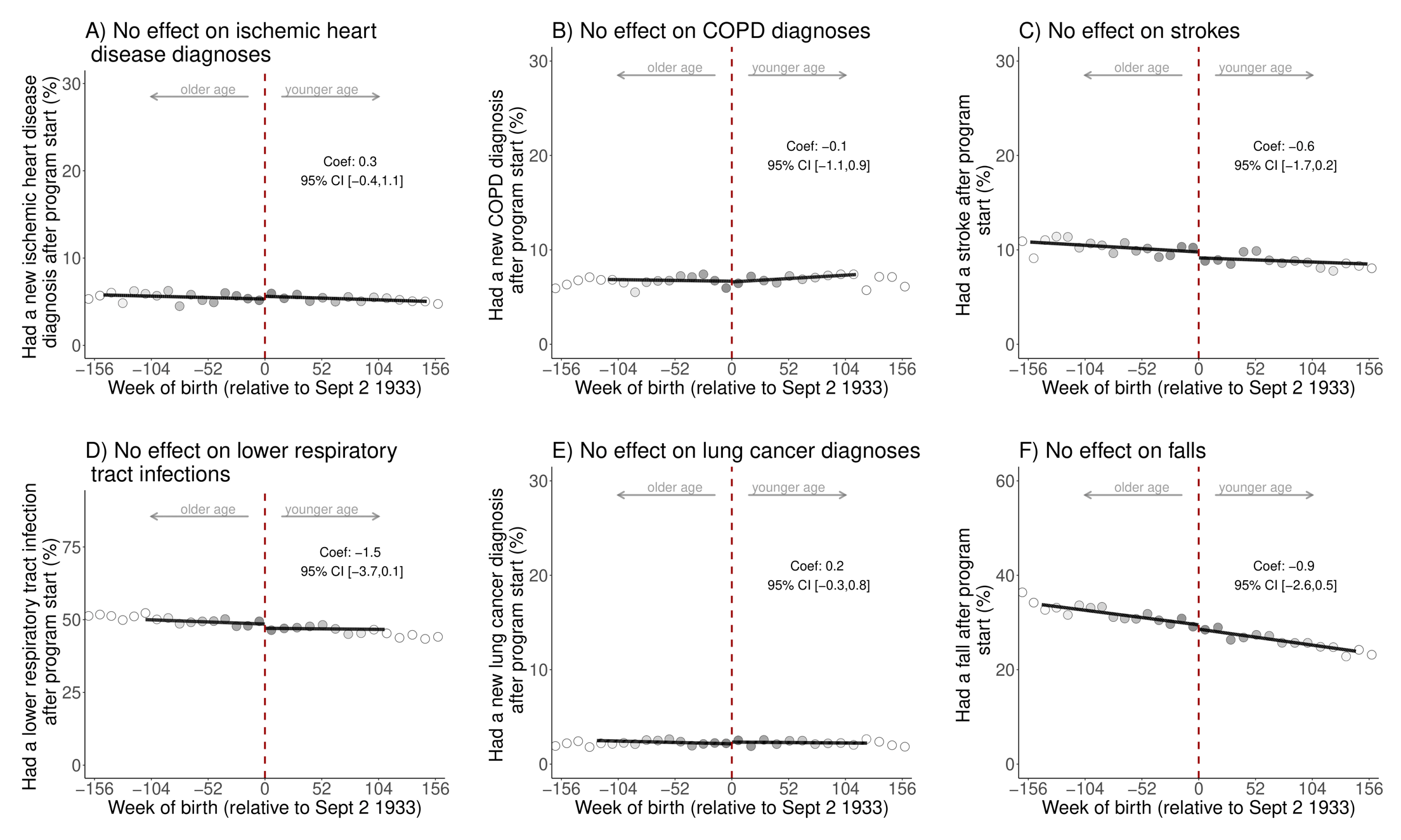

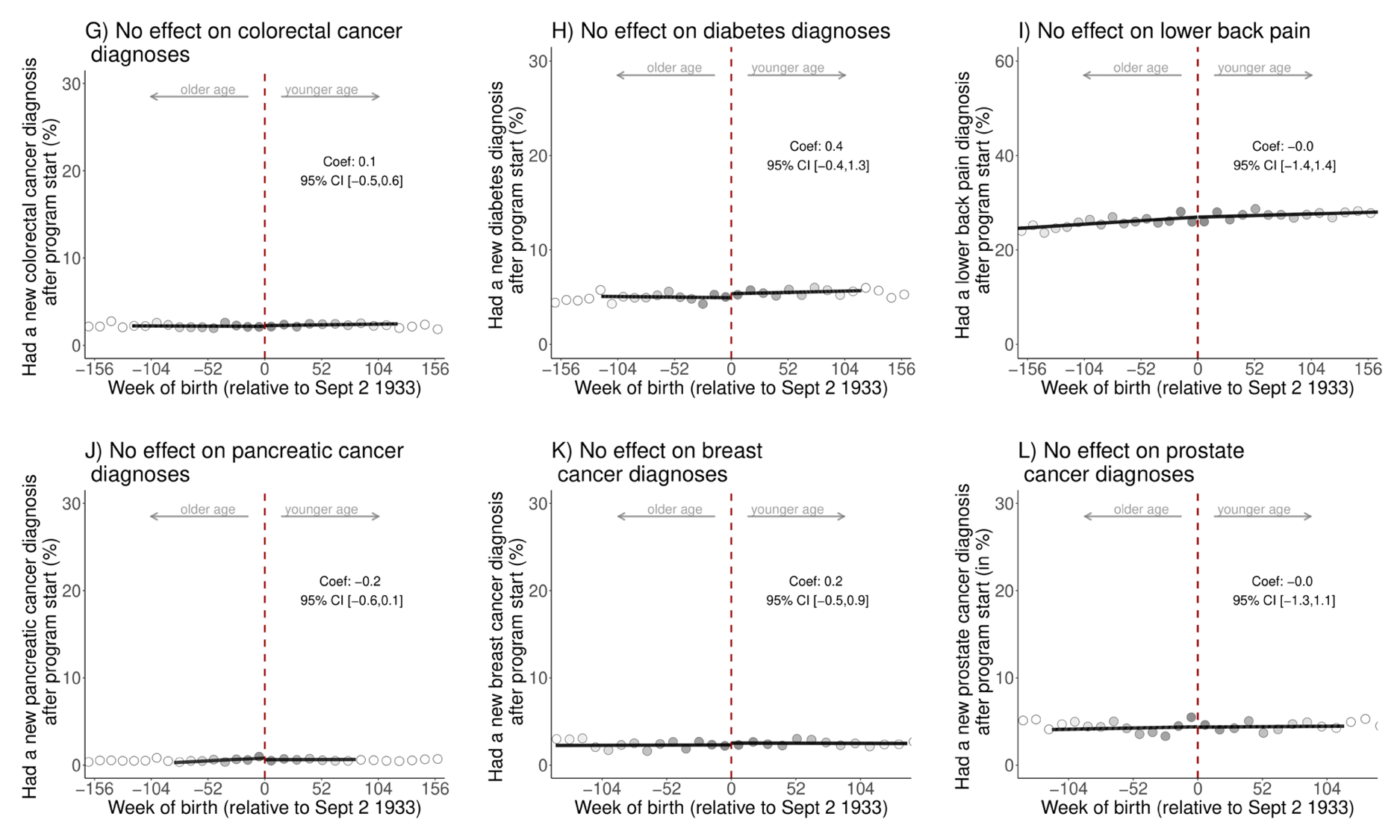


**Fig. 9**: No statistically significant effects of being eligible for the zoster vaccine on each of the ten (other than dementia) leading causes of disability-adjusted life years and mortality in Wales.^1,2,3,4,5,6,7^

^1^ The data source for this analysis was the SAIL database for Wales.

^2^ These analyses used the same follow-up period (September 1 2013 to September 1 2020) as our primary analyses for dementia shown in the main manuscript and were run on the same sample (n=282,541 adults).

^3^ The analysis of breast cancer diagnoses was restricted to women only (n=154,218 women); the analysis of prostate cancer diagnoses was restricted to men only (n=128,322 men).

^4^ Grey dots show the mean value for each 10-week increment in week of birth.

^5^ The grey shading of the dots is in proportion to the weight that observations from this 10-week increment received in the analysis.

^6^ P-values based on two-sided t-tests and unadjusted for multiple hypothesis testing: A) ischemic heart disease: 0.367, B) COPD: 0.858, C) stroke: 0.138, D) lower respiratory infections: 0.068, E) lung cancer: 0.429, F) falls: 0.180, G) colorectal cancer: 0.911, H) diabetes: 0.302, I) lower back pain: 0.993, J) pancreatic cancer: 0.163, K) breast cancer: 0.582, L) prostate cancer: 0.853.

^7^ When adjusting for multiple hypothesis testing (using Anderson’s sharpened q-values) jointly for all baseline conditions and preventive health measures shown in Extended Data Fig. 9 to 11, all p-values were equal to 1, except for the p-value for F) rheumatologic disease (p-value: 0.189).

Abbreviations: Coef=coefficient; CI=confidence interval; Sept=September; COPD=chronic obstructive pulmonary disease.

**
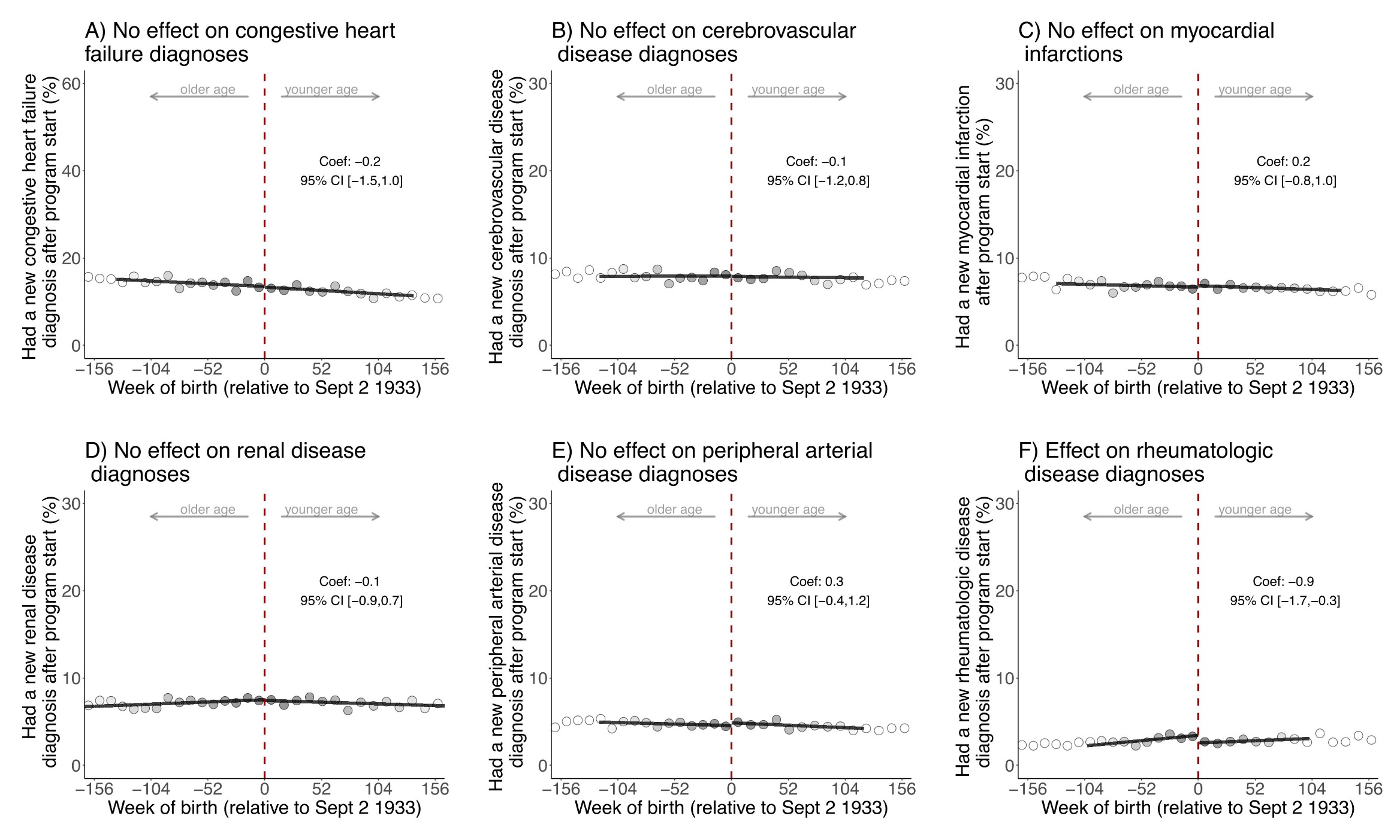
**

**
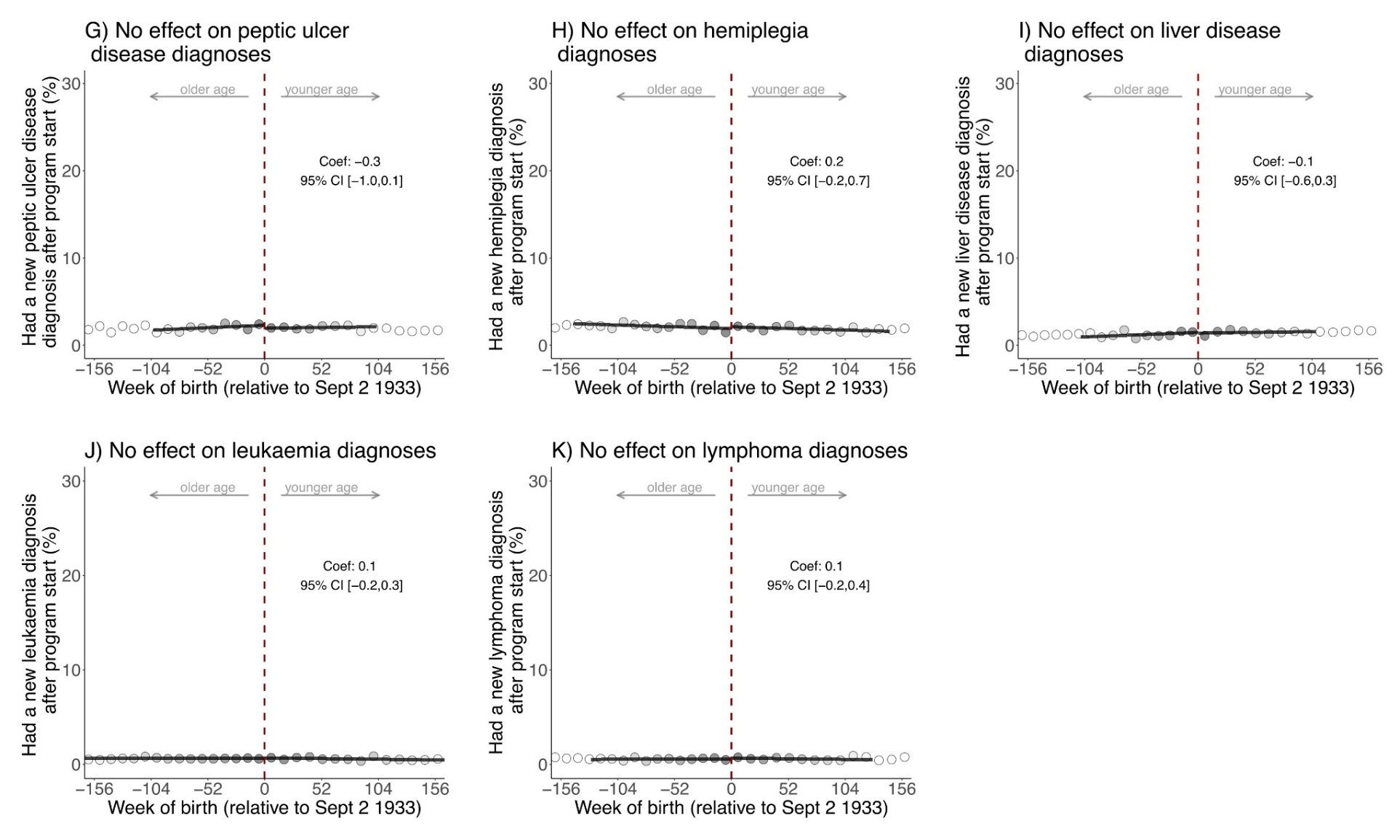
**

**Fig. 10**: Effects of being eligible for the zoster vaccine on each condition in the Charlson Comorbidity Index^1,2,3,4,5,6,7^

^1^ This figure only shows those conditions that are not already shown in Extended Data Fig. 9.

^2^ The data source for this analysis was the SAIL database for Wales.

^3^ These analyses used the same follow-up period (September 1 2013 to September 1 2020) as our primary analyses for dementia shown in the main manuscript and were run on the same sample (n=282,541 adults).

^4^ Grey dots show the mean value for each 10-week increment in week of birth.

^5^ The grey shading of the dots is in proportion to the weight that observations from this 10-week increment received in the analysis.

^6^ P-values based on two-sided t-tests and unadjusted for multiple hypothesis testing : A) congestive heart failure: 0.682, B) cerebrovascular disease: 0.687, C) myocardial infarction: 0.828, D) renal disease: 0.829, E) peripheral arterial disease: 0.384, F) rheumatologic disease: 0.006, G) peptic ulcer disease: 0.141, H) hemiplegia: 0.300, I) liver disease: 0.597, J) leukaemia: 0.499, K) lymphoma: 0.529.

^7^ When adjusting for multiple hypothesis testing (using Anderson’s sharpened q-values) jointly for all baseline conditions and preventive health measures shown in Extended Data Fig. 9 to 11, all p-values were equal to 1, except for the p-value for Fig. 9 Panel F) rheumatologic disease (p-value: 0.189).

Abbreviations: Coef=coefficient; CI=confidence interval; Sept=September.

**
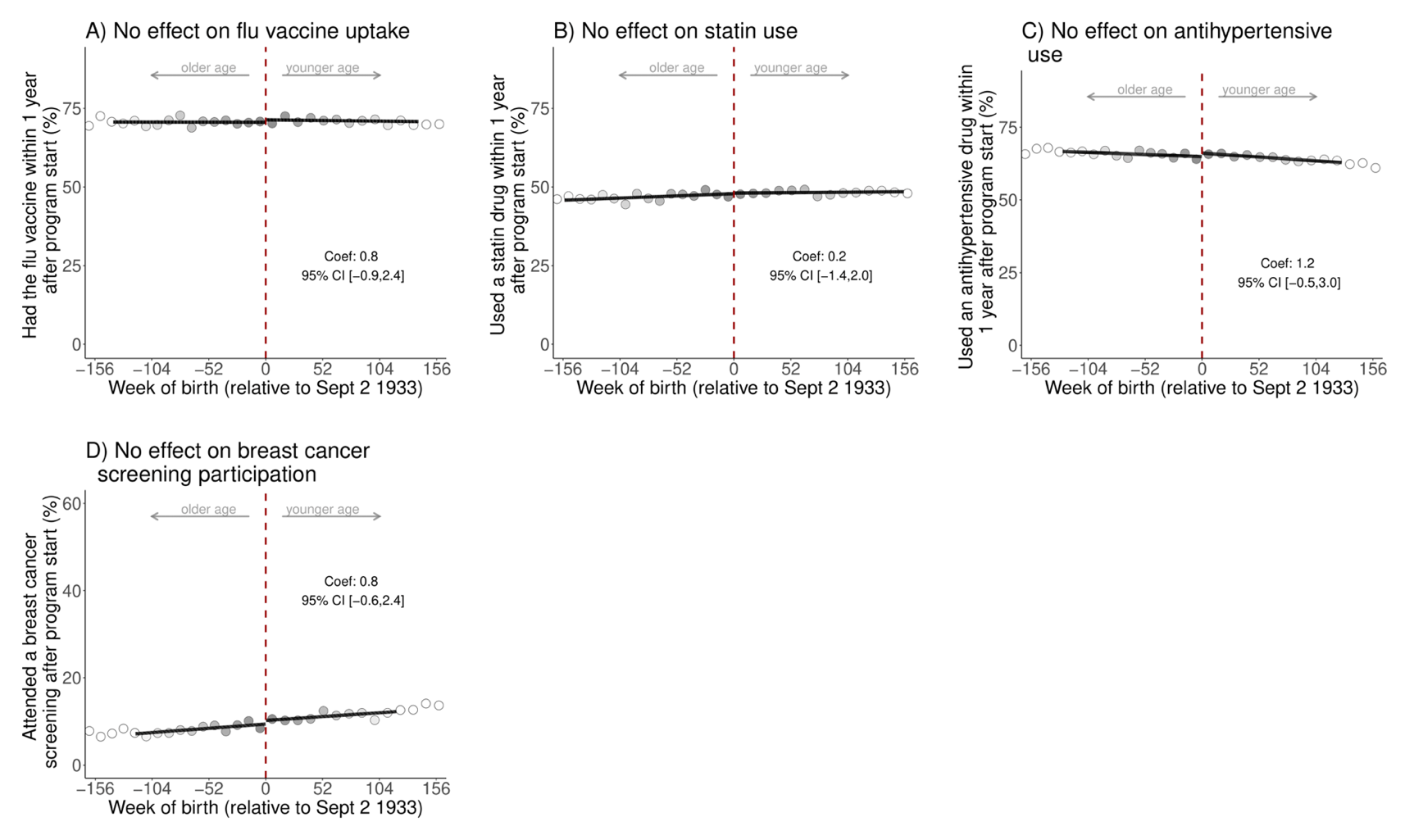
**

**Fig. 11:** No statistically significant effects of being eligible for the zoster vaccine on preventive health actions.^1,2,3,4,5,6,7^

^1^ The data source for this analysis was the SAIL database for Wales.

^2^ These analyses used the same follow-up period (September 1 2013 to September 1 2020) as our primary analyses for dementia shown in the main manuscript and were run on the same sample (n=282,541 adults).

^3^ Breast cancer screening participation was defined as having a record of referral to, attendance at, or a report from “breast cancer screening” or mammography. The analysis of breast cancer screening participation was restricted to women only (n=154,218 women).

^4^ Grey dots show the mean value for each 10-week increment in week of birth.

^5^ The grey shading of the dots is in proportion to the weight that observations from this 10-week increment received in the analysis.

^6^ P-values based on two-sided t-tests and unadjusted for multiple hypothesis testing: A) flu vaccine: 0.357, B) statins: 0.724, C) antihypertensive medications: 0.163, D) breast cancer screening: 0.248.

^7^ When adjusting for multiple hypothesis testing (using Anderson’s sharpened q-values) jointly for all baseline conditions and preventive health measures shown in Extended Data Fig. 9 to 11, all p-values were equal to 1, except for the p-value for Fig. 9 Panel F) rheumatologic disease (p-value: 0.189).

Abbreviations: Coef=coefficient; CI=95% confidence interval; Sept=September.

**
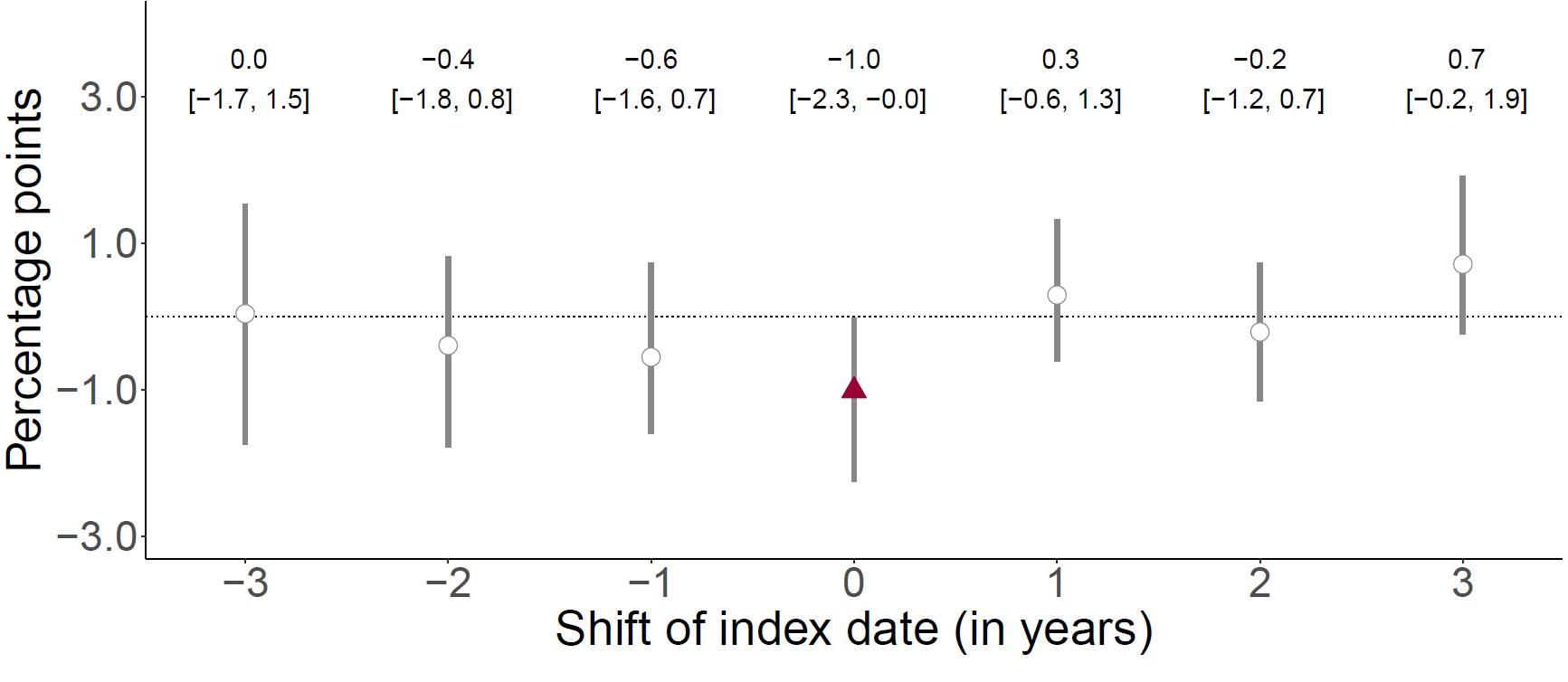
**

**Fig.12:** The September 2 date-of-birth threshold only has an effect on the occurrence of dementia over a five-year follow-up period in the year (2013) for which that date was used as eligibility threshold for zoster vaccination.^1,2,3,4,5,6^

^1^ The data source for this analysis was the SAIL database for Wales.

^2^ This analysis implements the identical analysis as for September 1 2013 (the date on which the zoster vaccine program started), for September 1 of each of the three years prior to and after 2013. For example, when moving the start date of the program to “-2” (i.e., September 1 2011), we started the follow-up period on September 1 2011 and compared individuals around the September 2 1931 eligibility threshold. The purpose of this analysis is to verify that the day-month (i.e., September 2) cutoff used for zoster vaccine eligibility was not also used for other interventions that affect dementia risk.

^3^ This analysis used a five-year follow-up period to allow each comparison to have the same length of follow-up.

^4^ Grey vertical bars depict 95% confidence intervals around the point estimates of the coefficients (two-sided t-tests).

^5^ White points depict statistically insignificant point estimates (p>0.05).

^6^ Sample sizes within the MSE-optimal bandwidth for shifts from -3 to 3 years are 38,037 adults, 60,755 adults, 73,023 adults, 80,775 adults, 106,042 adults, 112,035 adults, and 77200 adults, respectively.


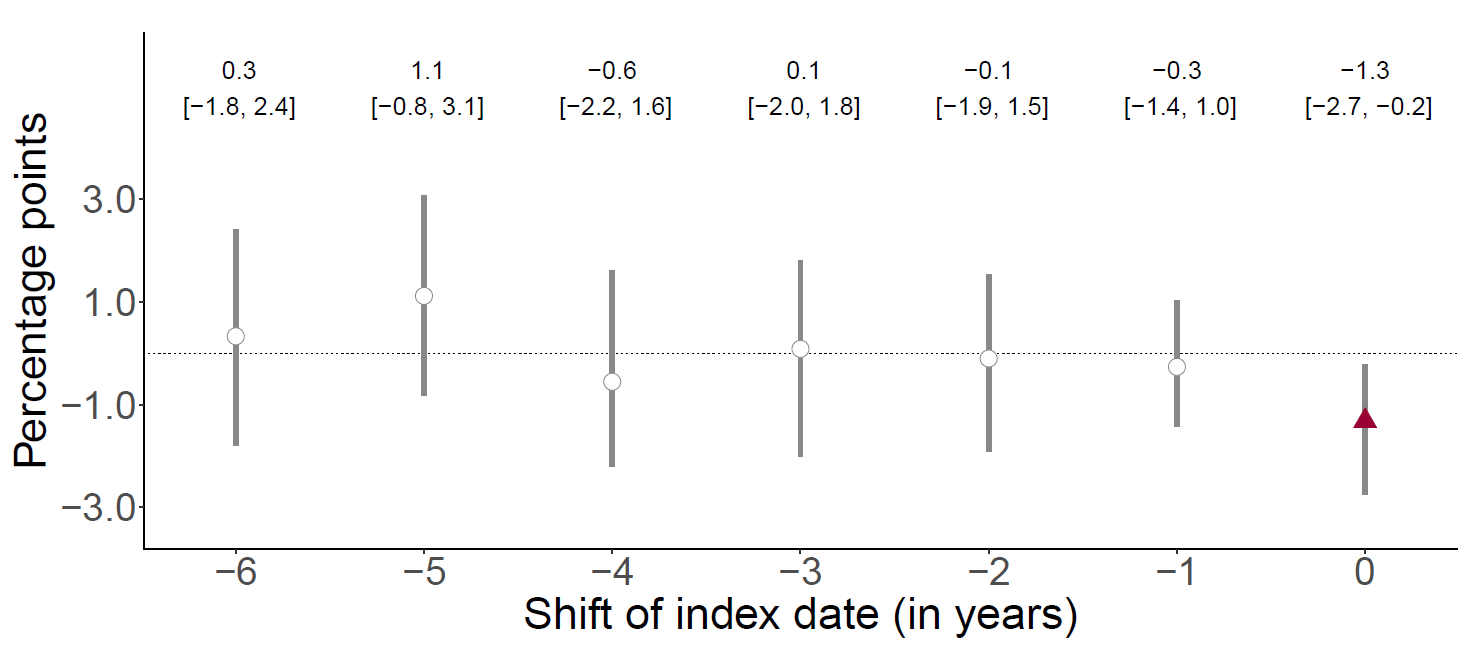


**Fig. 13**: The September 2 date-of-birth threshold only has an effect on the occurrence of dementia over a seven-year follow-up period in the year (2013) for which that date was used as eligibility threshold for zoster vaccination.^1,2,3,4,5^

^1^ The data source for this analysis was the SAIL database for Wales.

^2^ This analysis implements the identical analysis as for September 1 2013 (the date on which the zoster vaccine program started), for September 1 of each of the six years preceding 2013. For example, when moving the start date of the program to “-2” (i.e., September 1 2011), we started the follow-up period on September 1 2011 and compared individuals around the September 2 1931 eligibility threshold. The purpose of this analysis is to verify that the day-month (i.e., September 2) cutoff used for zoster vaccine eligibility was not also used for other interventions that affect dementia risk.

^3^ Grey vertical bars depict 95% confidence intervals around the point estimates of the coefficients (two-sided t-tests).

^4^ White points depict statistically insignificant point estimates (p>0.05).

^5^ Sample sizes within the MSE-optimal bandwidth for shifts from -6 to 0 years are 32,402 adults, 36,415 adults, 36,319 adults, 38,322 adults, 48,383 adults, 91,414 adults, and 83,167 adults, respectively.


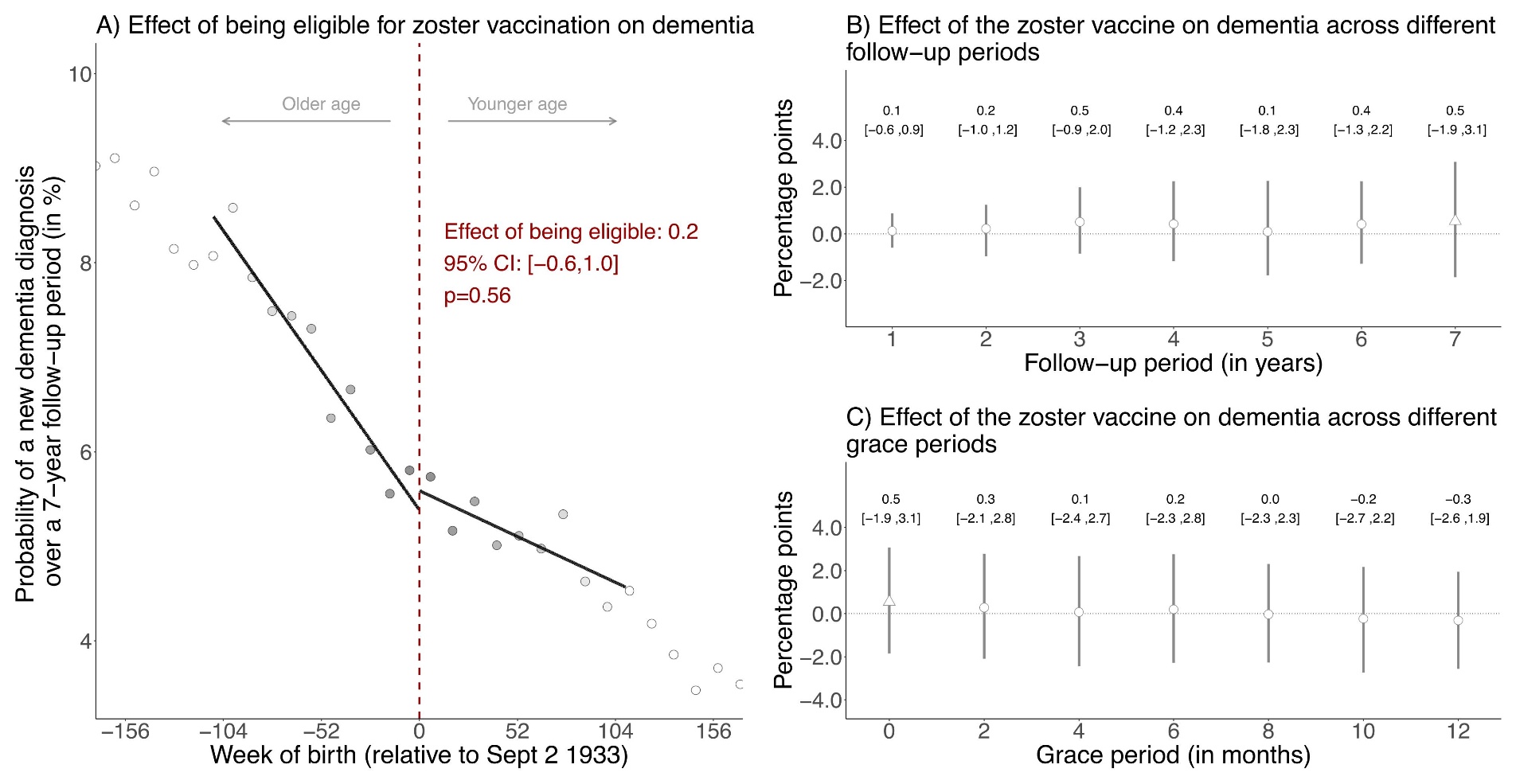


**Fig. 14**: No effects on new diagnoses of dementia diagnosed in the seven years *prior* to the start of the zoster vaccine program (i.e., between September 1 2006 to August 31 2013).^1,2,3,4,5,6,7,8,9^

^1^ The data source for this analysis was the SAIL database for Wales.

^2^ This figure shows the results from the identical analysis as implemented for our primary analysis (for which the results are shown in Fig. 3 in the main manuscript) except that we followed individuals from September 1 2006 to August 31 2013 instead of from September 1 2013 to August 31 2020. This analysis thus compares the exact same date-of-birth cohorts to each other and has the same length of follow-up as our primary analysis, but uses the seven years prior to the start of the zoster vaccine rollout as follow-up period instead of the seven years after program start. The purpose of this analysis is to verify that the same date-of-birth eligibility date used for zoster vaccine eligibility was not used for other interventions in the past that affect dementia risk.
^3^ Triangles (rather than points) depict our primary specification.

^4^ Red (as opposed to white) fillings denote statistical significance (p<0.05).
^5^ With “grace periods” we refer to time periods since the index date after which follow-up time is considered to begin to allow for the time needed for a full immune response to develop after vaccine administration.
^6^ Grey vertical bars depict 95% confidence intervals around the point estimates of the coefficients (two-sided t-tests).

^7^ Grey dots in Panel A show the mean value for each 10-week increment in week of birth.

^8^ The grey shading of the dots is in proportion to the weight that observations from this 10-week increment received in the analysis.

^9^ For panel A, the mean squared error-optimal bandwidth is 109.4 weeks (90,932 adults) and the p-value is calculated based on a two-sided t-test. For panel B and C, in our primary specification the mean squared error-optimal bandwidth is 85.4 weeks (70,853 adults).

Abbreviations: Coef=coefficient; CI=95% confidence interval; Sept=September.


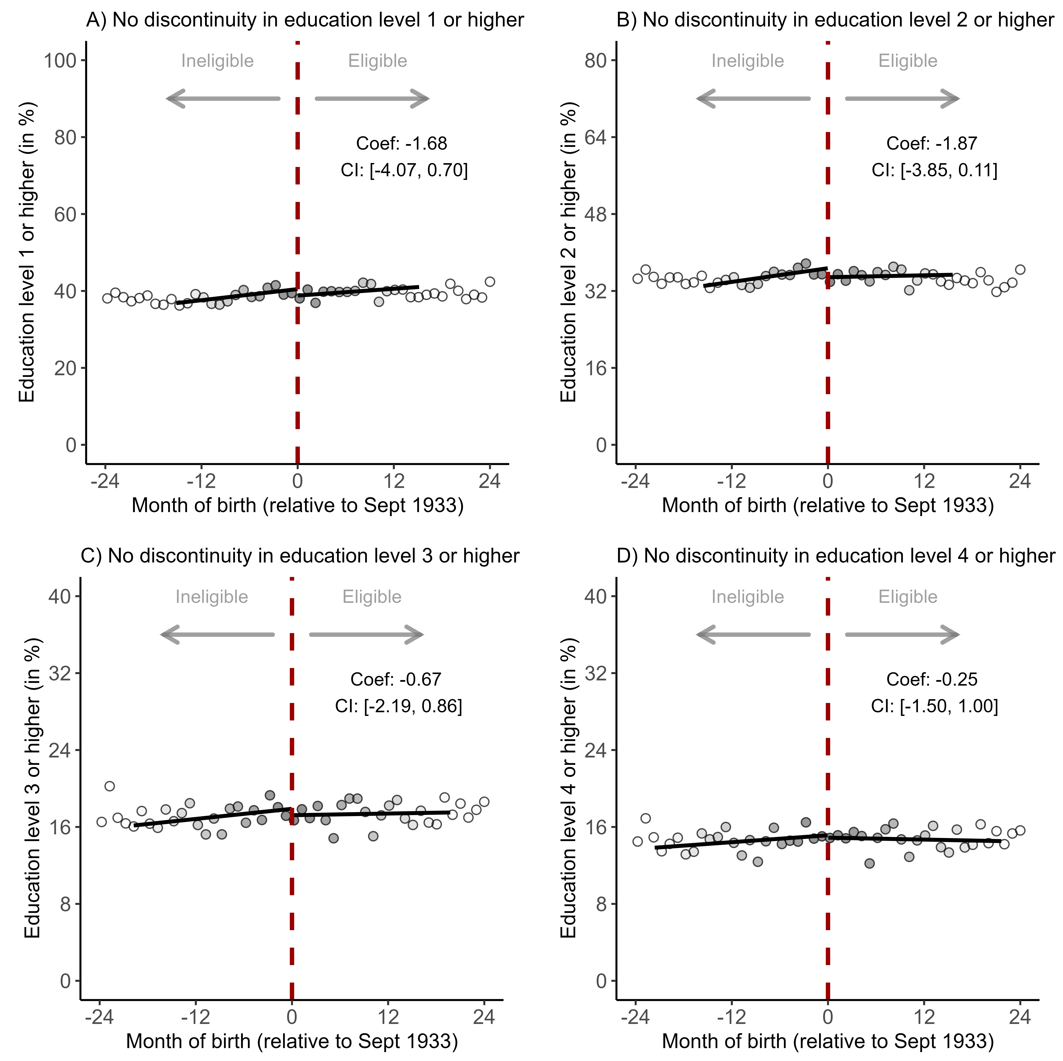


**Fig. 15:** No statistically significant effects of being eligible for the zoster vaccine on education levels in Wales.^1,2,3,4^

^1^ The data source for this analysis was the 2011 Census in Wales.

^2^ Grey dots in show the mean value for each birth-month.

^3^ The grey shading of the dots is in proportion to the weight that observations from this birth-month received in the analysis.

^4^ The linear regression lines are drawn only in the mean squared error-optimal bandwidth.

Abbreviations: Coef=coefficient; CI=95% confidence interval; Sept=September.


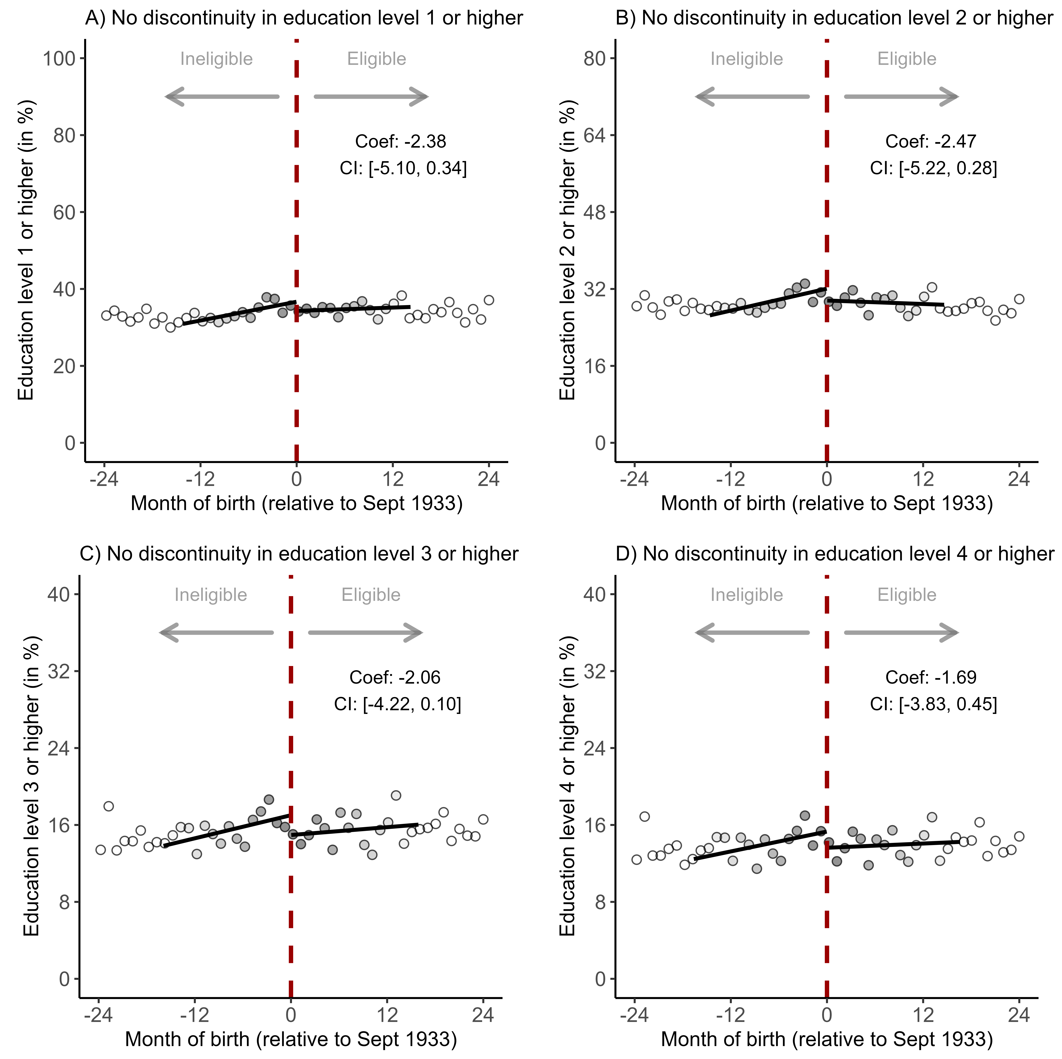


**Fig. 16:** No statistically significant effects of being eligible for the zoster vaccine on education levels in Wales among females.^1,2,3,4^

^1^ The data source for this analysis was the 2011 Census in Wales.

^2^ Grey dots in show the mean value for each birth-month.

^3^ The grey shading of the dots is in proportion to the weight that observations from this birth-month received in the analysis.

^4^ The linear regression lines are drawn only in the mean squared error-optimal bandwidth.

Abbreviations: Coef=coefficient; CI=95% confidence interval; Sept=September.


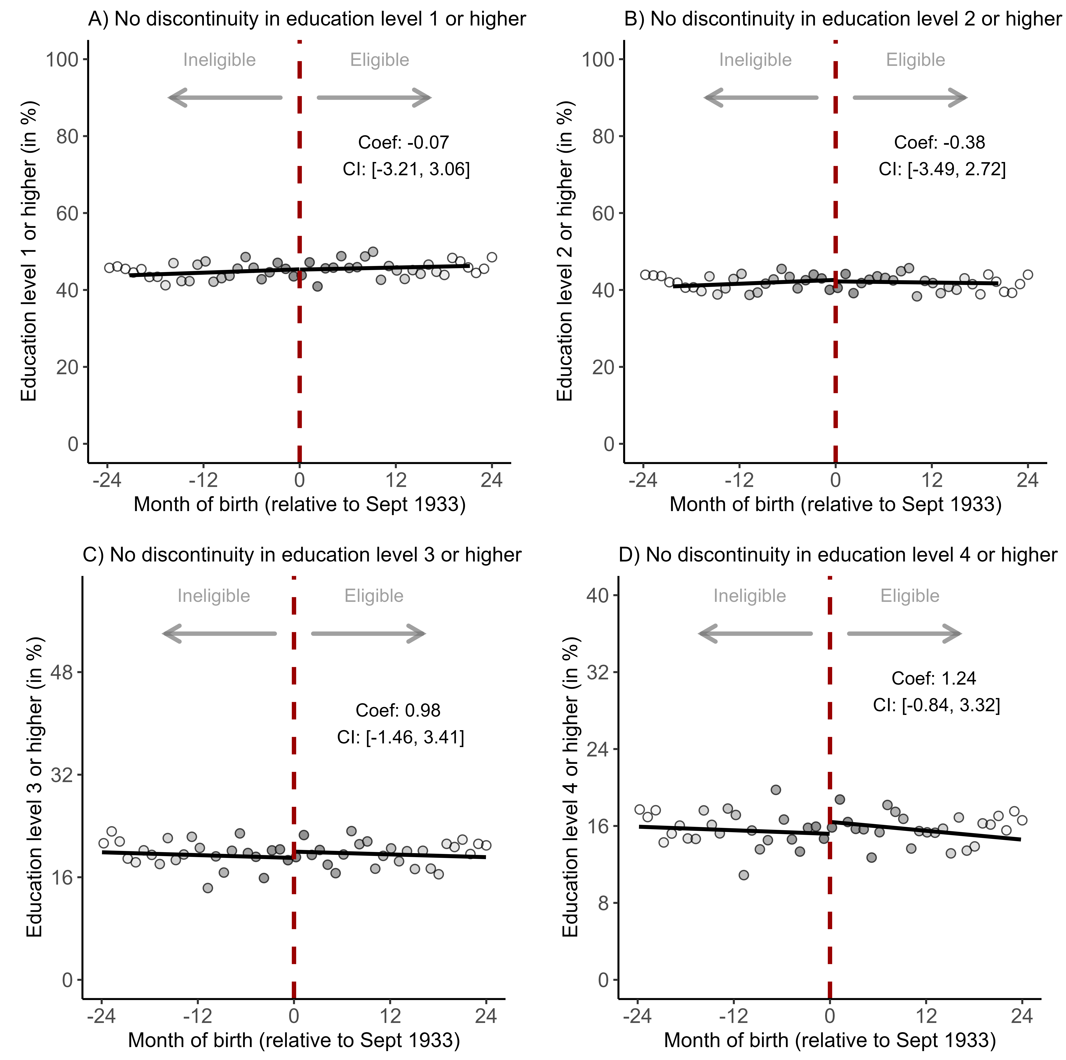


**Fig. 17:** No statistically significant effects of being eligible for the zoster vaccine on education levels in Wales among males.^1,2,3,4^

^1^ The data source for this analysis was the 2011 Census in Wales.

^2^ Grey dots in show the mean value for each birth-month.

^3^ The grey shading of the dots is in proportion to the weight that observations from this birth-month received in the analysis.

^4^ The linear regression lines are drawn only in the mean squared error-optimal bandwidth.

Abbreviations: Coef=coefficient; CI=95% confidence interval; Sept=September.


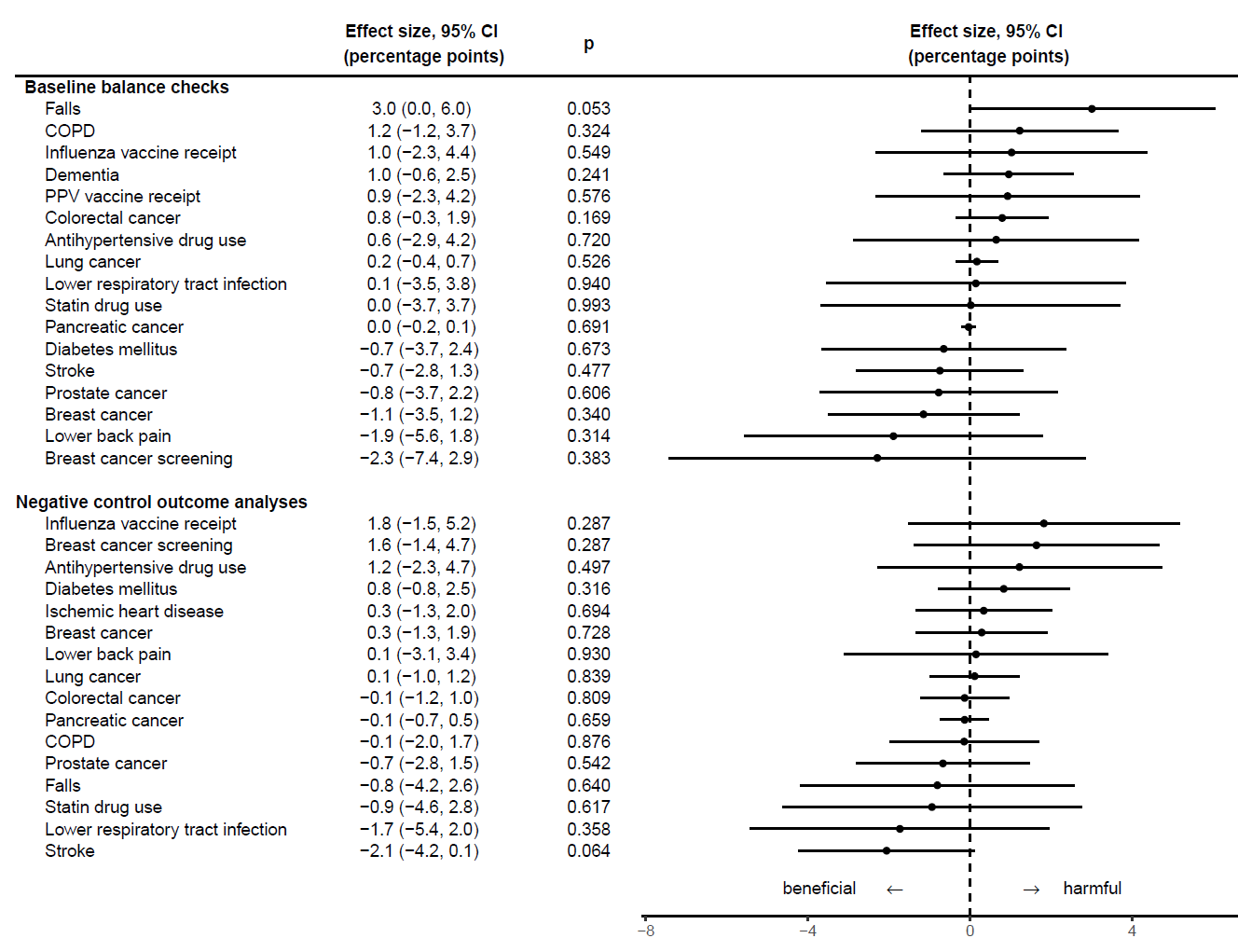


**Fig. 18**: No statistically significant effects of the zoster vaccine on each of the ten (other than dementia) leading causes of disability-adjusted life years and mortality, nor the take-up of preventive health measures in Wales before and after program start, using a difference-in-differences IV design.^1,2,3,4,5,6,7^

^1^ The data source for this analysis was the SAIL database for Wales.

^2^ Baseline balance checks examined occurrence of the listed outcomes at any time before September 1 2013.

^3^ Negative control outcome analyses used the same follow-up period (September 1 2013 to September 1 2020) as our analysis for dementia shown in the main manuscript (n=96,767 adults).

^4^ The analysis of breast cancer diagnoses and screenings was restricted to women only (n=55,900 women); the analysis of prostate cancer diagnoses was restricted to men only (n=40,867 men).

^5^ For the baseline balance check, influenza vaccine receipt was defined as having received the influenza vaccine within one year before September 1 2013; for the negative control outcome analysis, influenza vaccine receipt was defined as having received the influenza vaccine within one year after September 1 2013.

^6^ PPV vaccine receipt was defined as having received the pneumococcal polysaccharide vaccine at any time before September 1 2013.

^7^ Error bars depict 95% confidence intervals around the point estimates of the coefficients (two-sided t-tests).

Abbreviations: COPD=chronic obstructive pulmonary disease; PPV=pneumococcal polysaccharide vaccine.

**
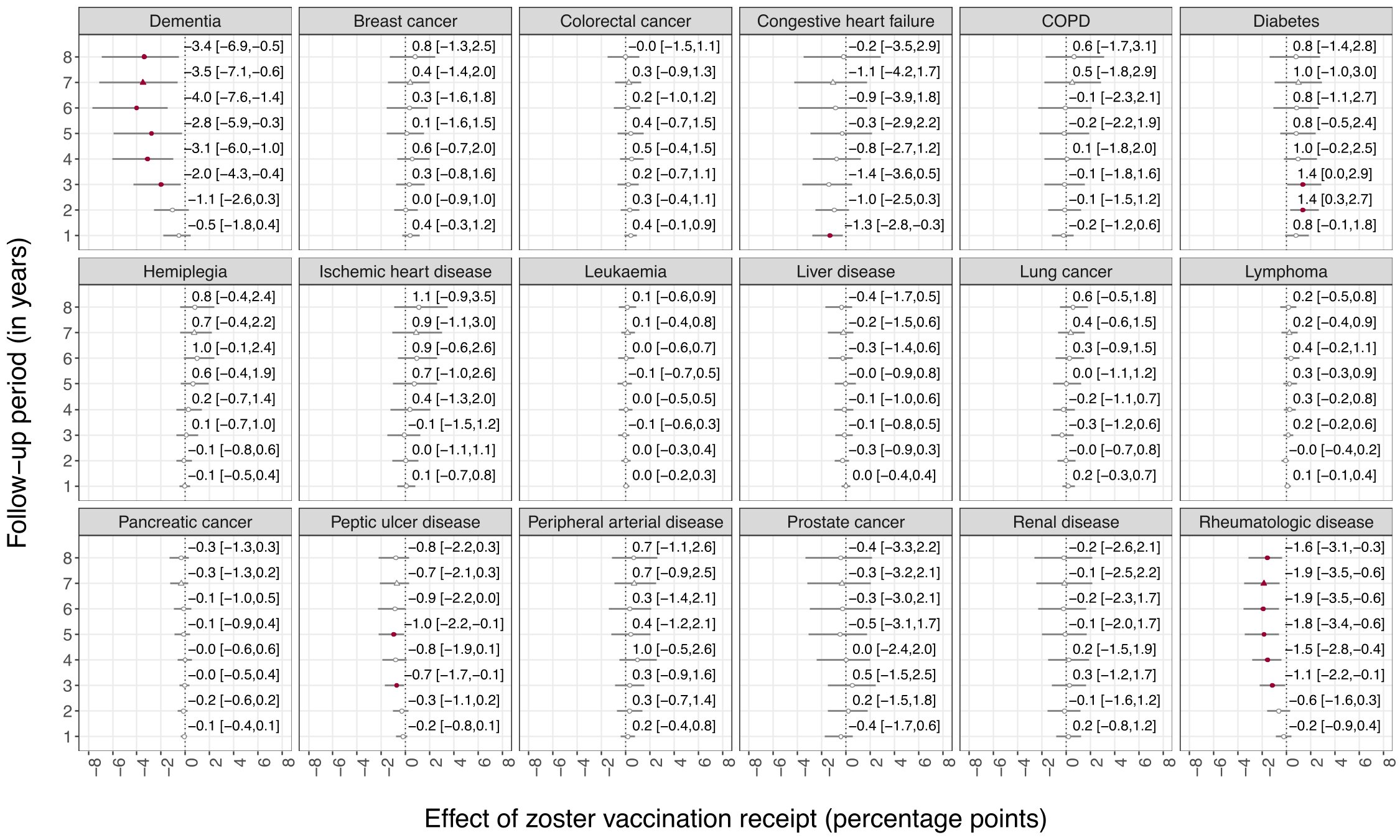
**

**Fig. 19**: Effects of zoster vaccination receipt on chronic conditions that are either among the ten leading causes of disability-adjusted life years and mortality in Wales, or part of the Charlson Comorbidity Index.^1,2,3,4,5,6^

^1^ The data source for this analysis was the SAIL database for Wales.

^2^ These analyses used different follow-up periods of 1 to 8 years to define the outcomes and estimated the effect of vaccination receipt using the fuzzy regression discontinuity design. Our primary follow-up period is seven years, as indicated by the triangular shape for the point estimate.

^3^ All analyses were run on the same sample as our primary analyses for dementia shown in the main manuscript (n=282,541 adults).

^4^ Breast cancer screening participation was defined as having a record of referral to, attendance at, or a report from “breast cancer screening” or mammography. The analysis of breast cancer screening participation was restricted to women only (n=154,218).

^5^ The analysis of breast cancer diagnoses was restricted to women only (n=154,218 women); the analysis of prostate cancer diagnoses was restricted to men only (n=128,322 men).

^6^ Error bars depict 95% confidence intervals around the point estimates of the coefficients (two-sided t-tests).

Abbreviations: COPD=chronic obstructive pulmonary disease.


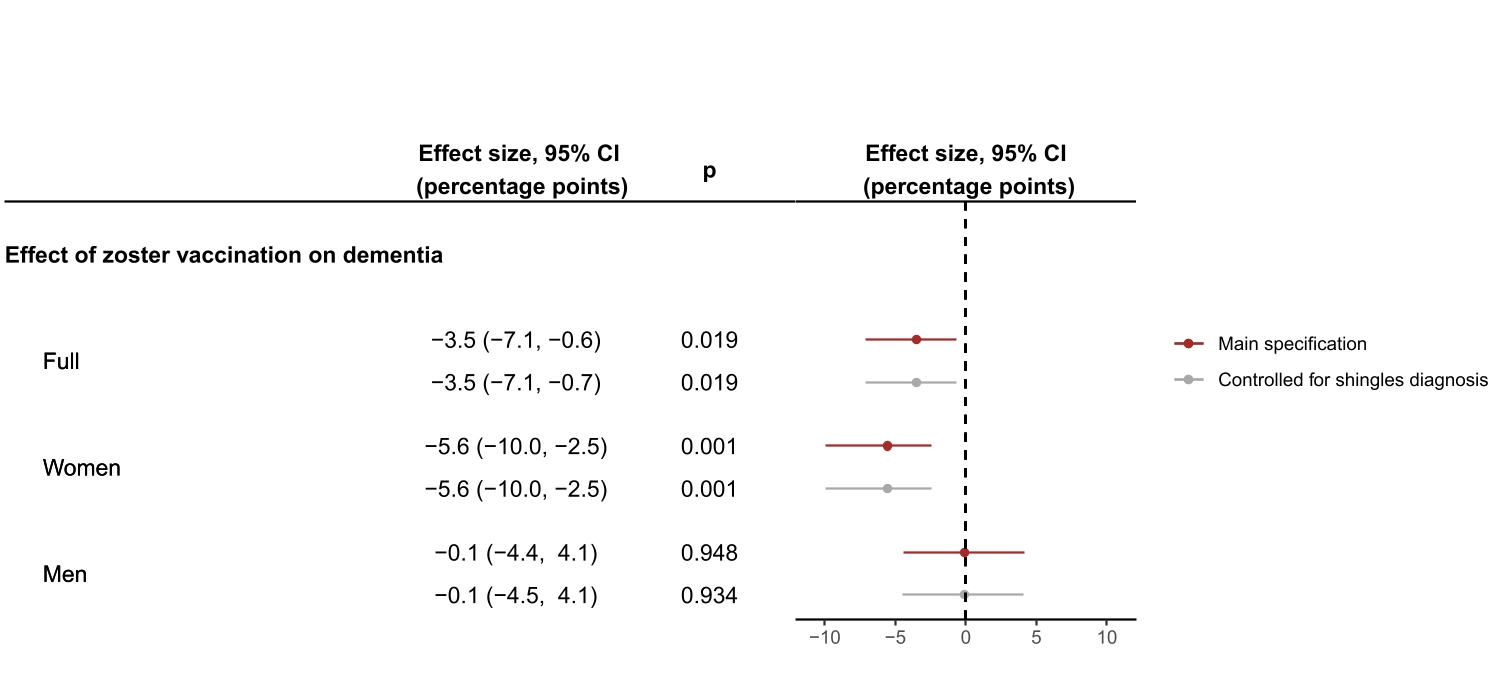


**Fig. 20**: Effect estimates of having received the zoster vaccine on new diagnoses of dementia with versus without adjustment for whether an individual received a diagnosis of shingles during the follow-up period.^1,2,3^
^1^ The data source for this analysis was the SAIL database for Wales.

^2^ The analyses for the full sample were run on the same sample as our primary analyses for dementia shown in the main manuscript (n=282,541 adults). The analyses for women consists of the n=154,218 women in this sample, the analyses for men was restricted to the n=128,322 men in this sample.

^3^ Error bars depict 95% confidence intervals around the point estimates of the coefficients (two-sided t-tests).

^
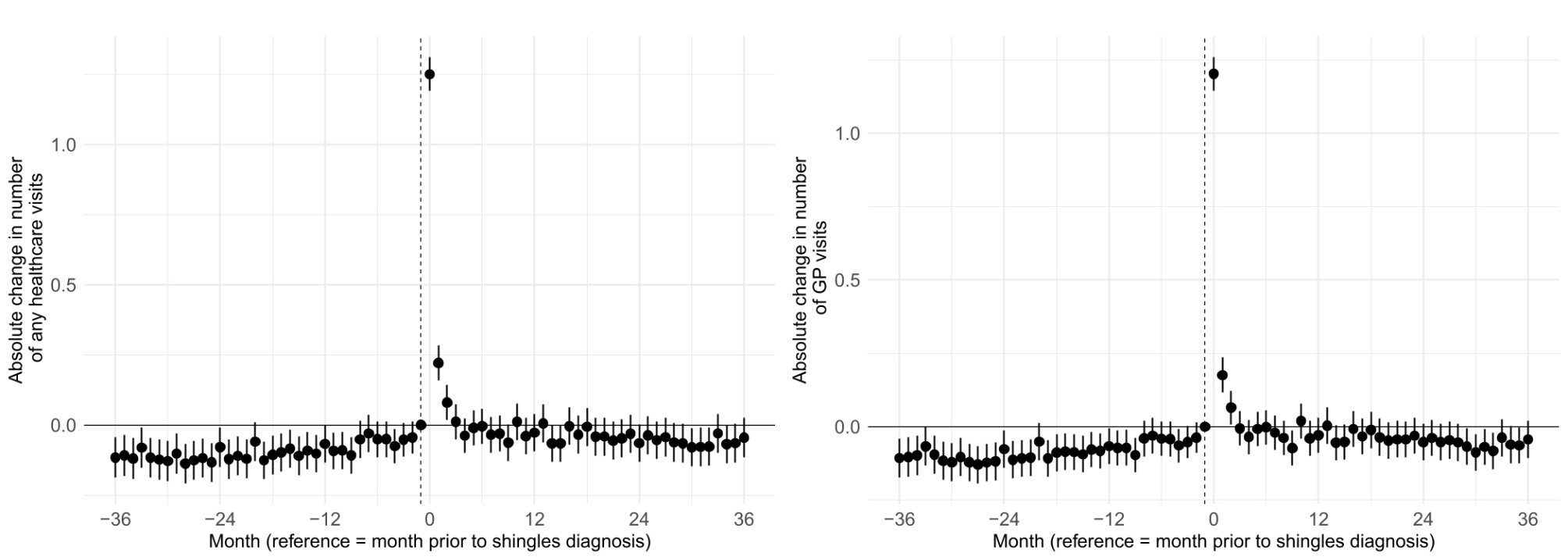

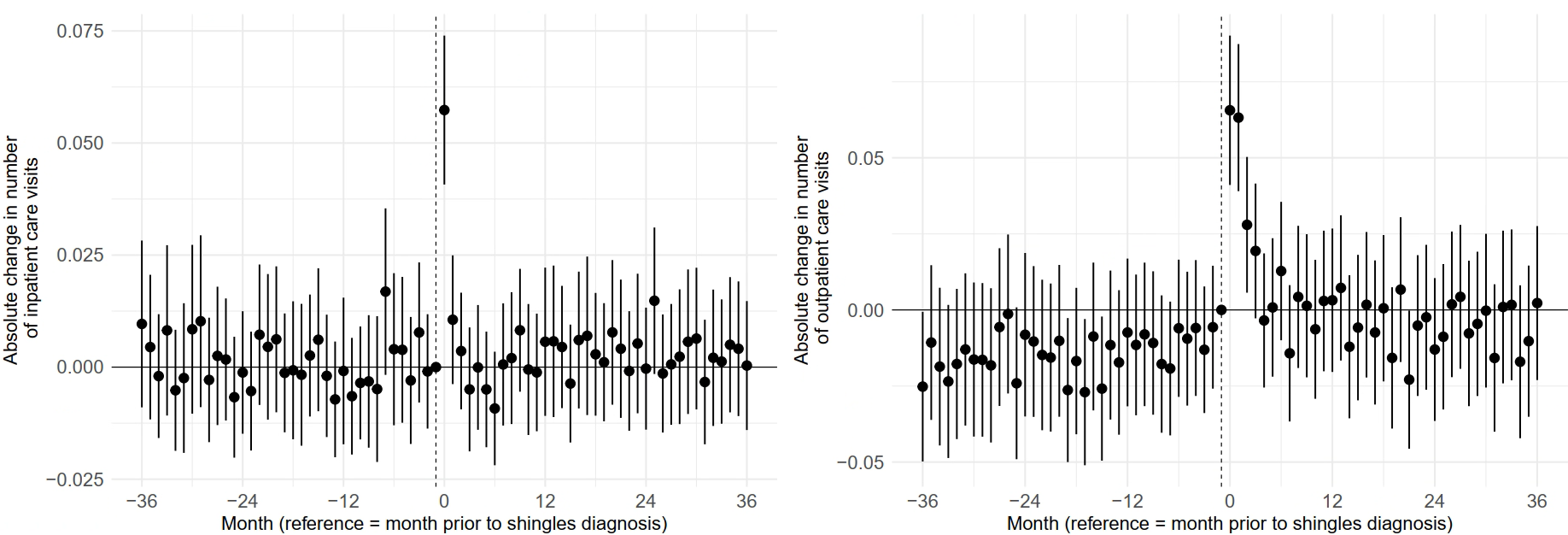
^


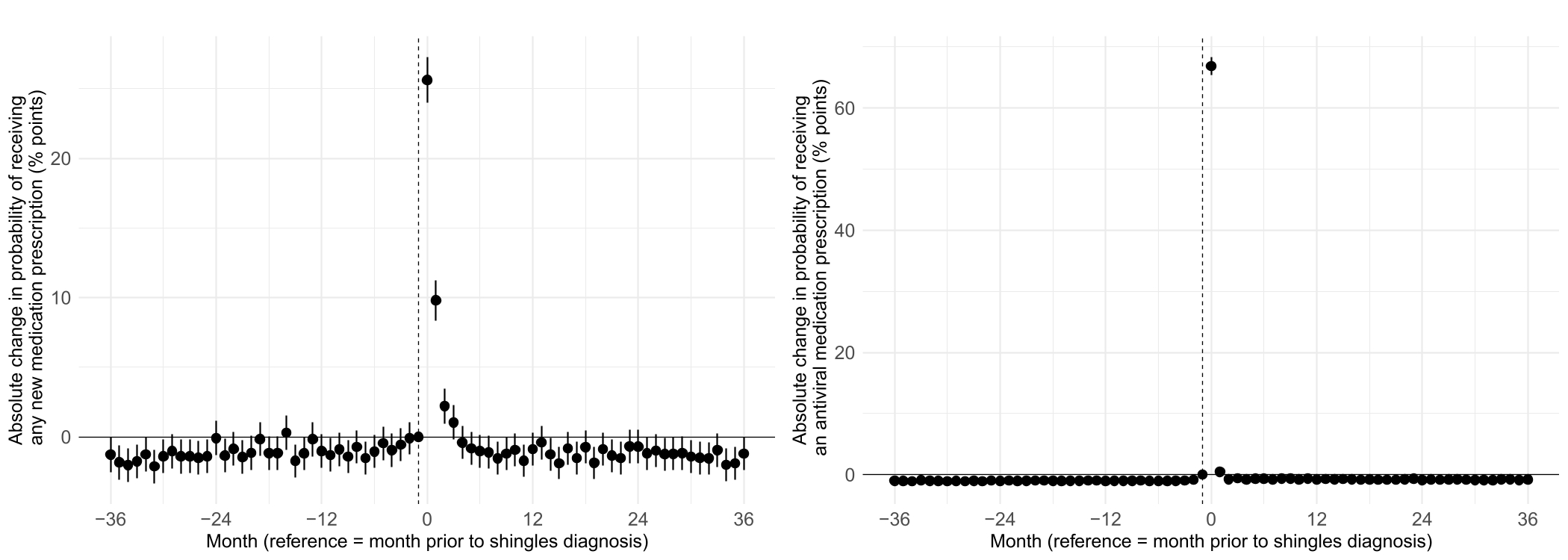


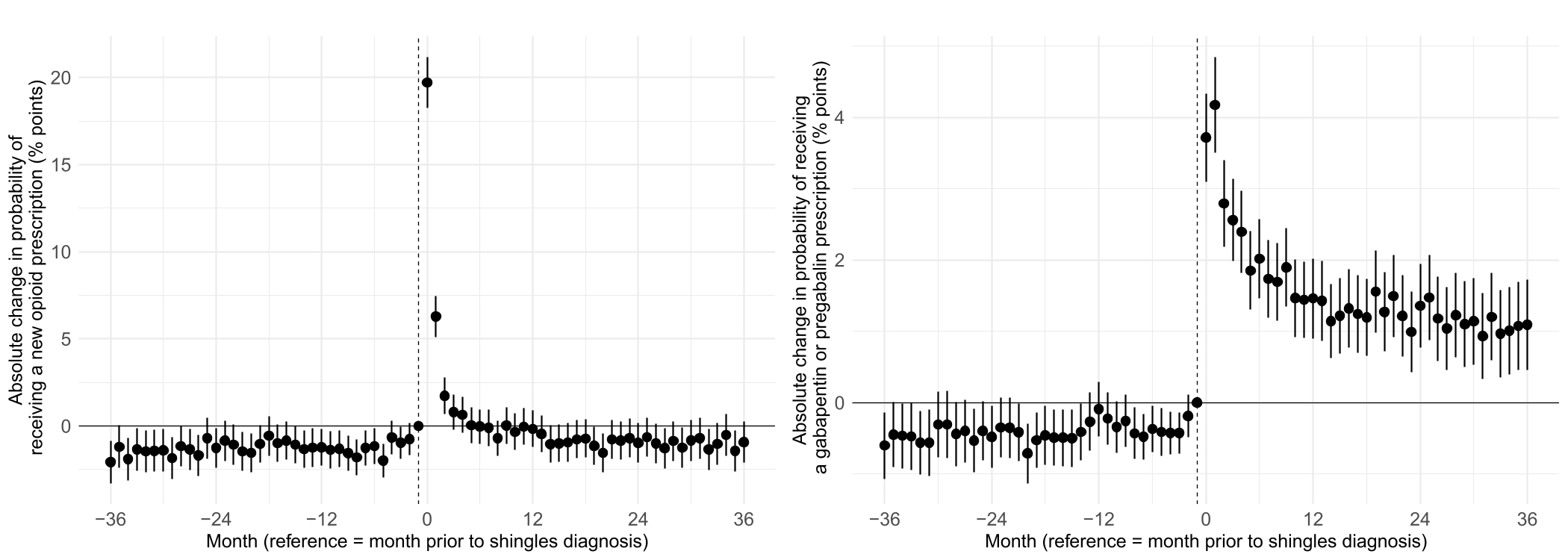


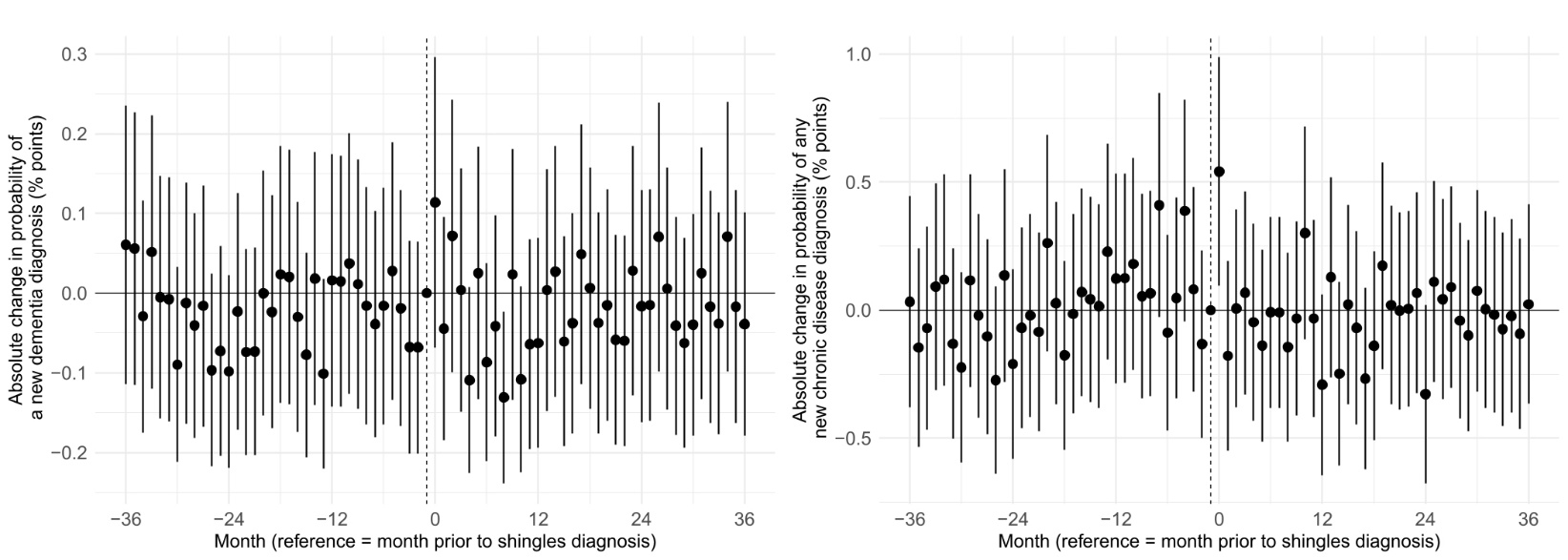


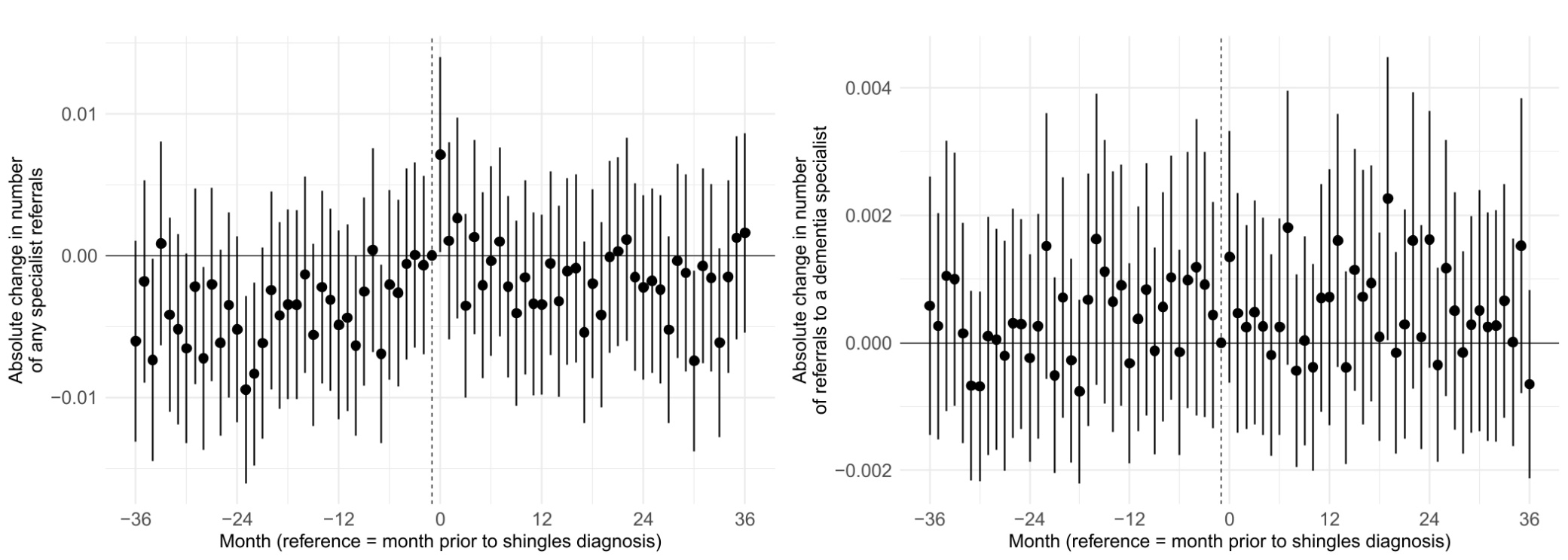


**Fig. 21**: Event-study effects for the effect of shingles diagnosis on various outcomes in each of 36 months after the shingles diagnosis.^1,2,3,4,5,6,7
1^ The data source for this analysis was the SAIL database for Wales.

^2^ Each point estimate shown is in comparison to the month prior to the shingles diagnosis. Month 0 is the month of the shingles diagnosis.

^3^ “Dementia specialist” was defined as a neurologist or psychiatrist.

^4^ “Any new medication” was defined as any of 216 medications that were found to be associated with an increased risk of dementia in an independent study in the SAIL database (reference in the Main Text).

^5^ “Any new chronic disease” was defined as any chronic condition that is either among the leading ten causes of disability-adjusted life years or mortality among individuals in Wales aged 70+ years (as per the Global Burden of Disease 2019 study), or among the conditions in the Charlson Comorbidity Index.

^6^ The sample of all event-study plots consists of n=59,158 adults.

^7^ Error bars depict 95% confidence intervals around the point estimates of the event-study coefficients.

Abbreviations: GP=general practitioner.


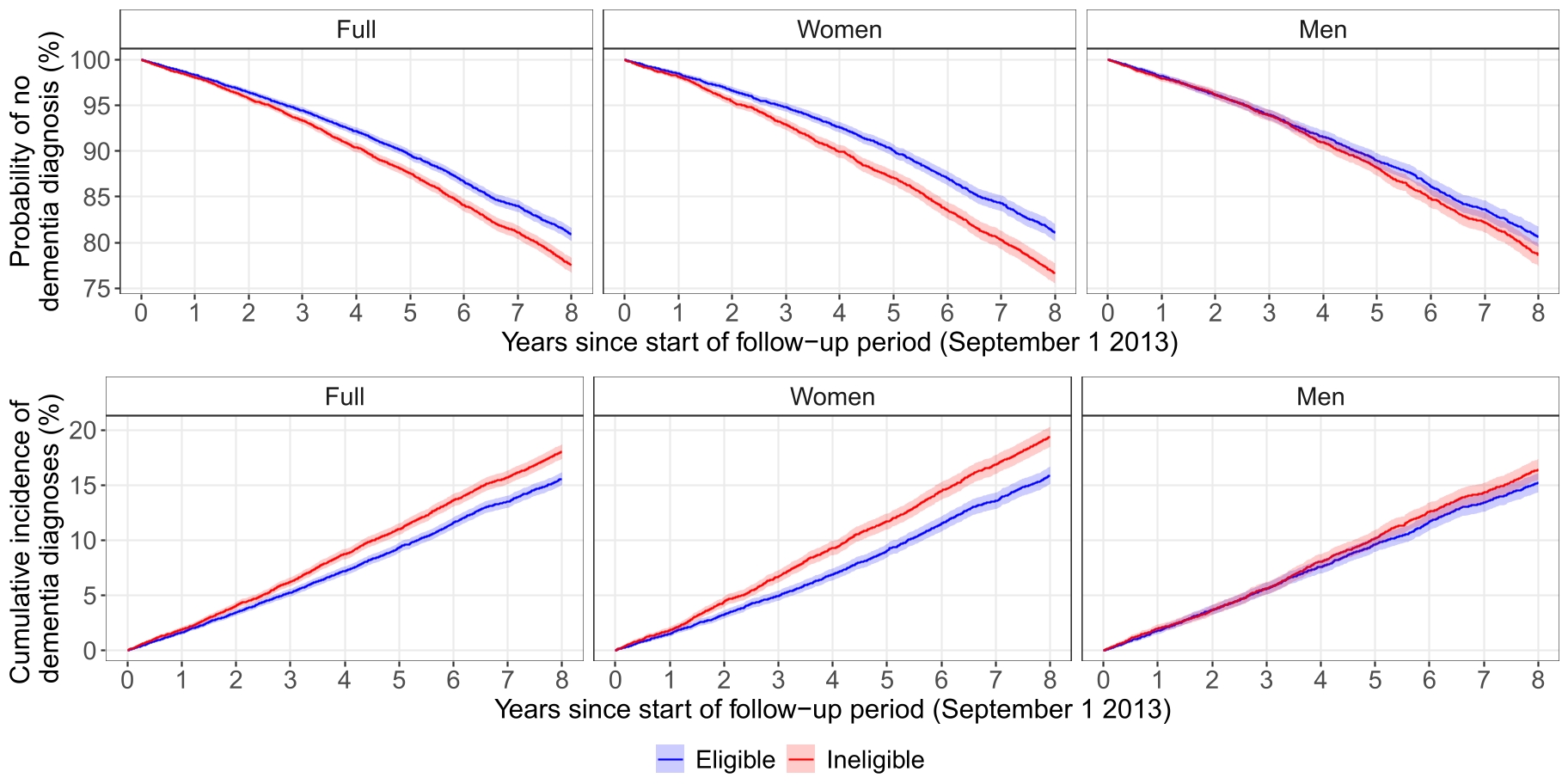


**Fig. 22**: Kaplan-Meier and cumulative incidence curves for dementia for those who were eligible versus ineligible for zoster vaccination.^1,2,3^
^1^ The data source for this analysis was the SAIL database for Wales.

^2^ The bandwidth used in this analysis was 54.4 weeks on either side of the September 2 1933 date-of-birth eligibility threshold.

^3^ Red and blue shadings depict 95% confidence intervals around the estimates labeled on the respective y-axes.

**
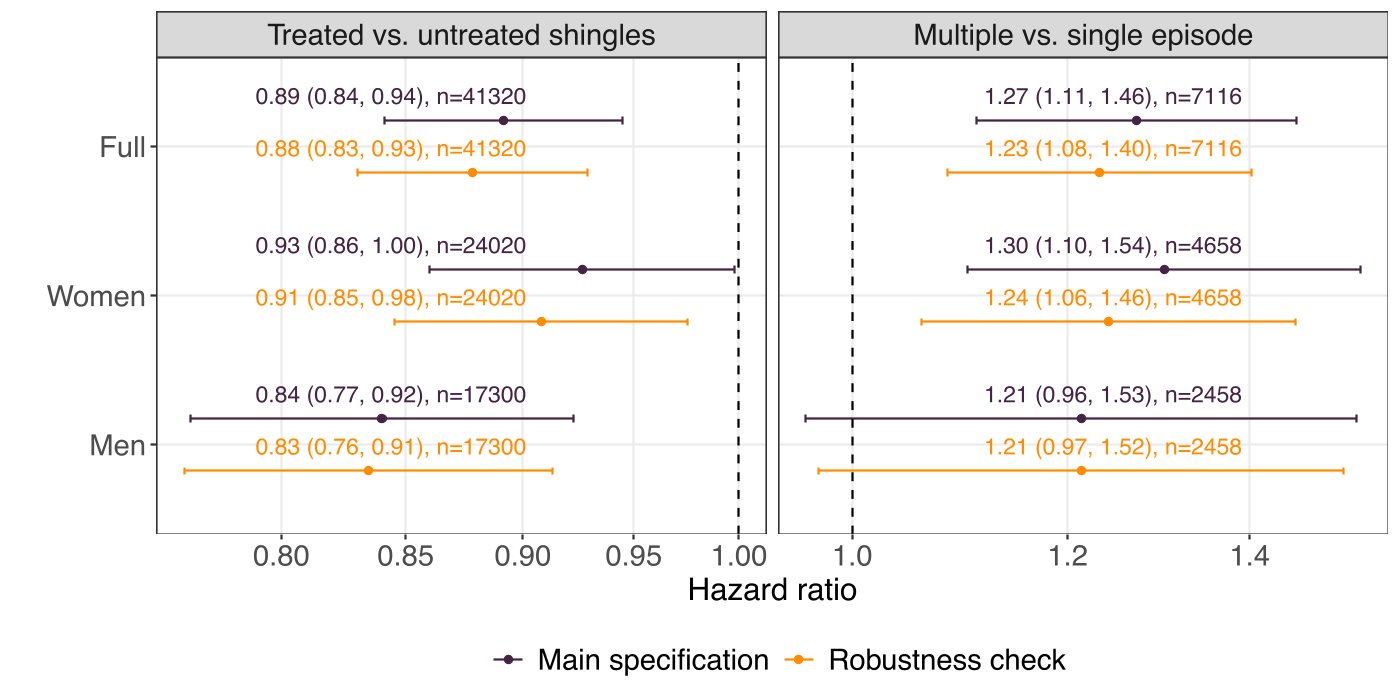
**

**Fig. 23**: Hazard ratios comparing the association of dementia with treated versus untreated shingles episodes, as well as with multiple versus a single shingles episode.^1,2,3,4^
^1^ The data source for this analysis was the SAIL database for Wales.

^2^ Hazard ratios were plotted on a logarithmic scale.

^3^ “Robustness check” refers to the analysis when requiring that a new diagnosis of dementia must have been made at least 12 months after the date of the first shingles diagnosis.

^4^ Error bars depict 95% confidence intervals around the point estimates of the hazard ratios.


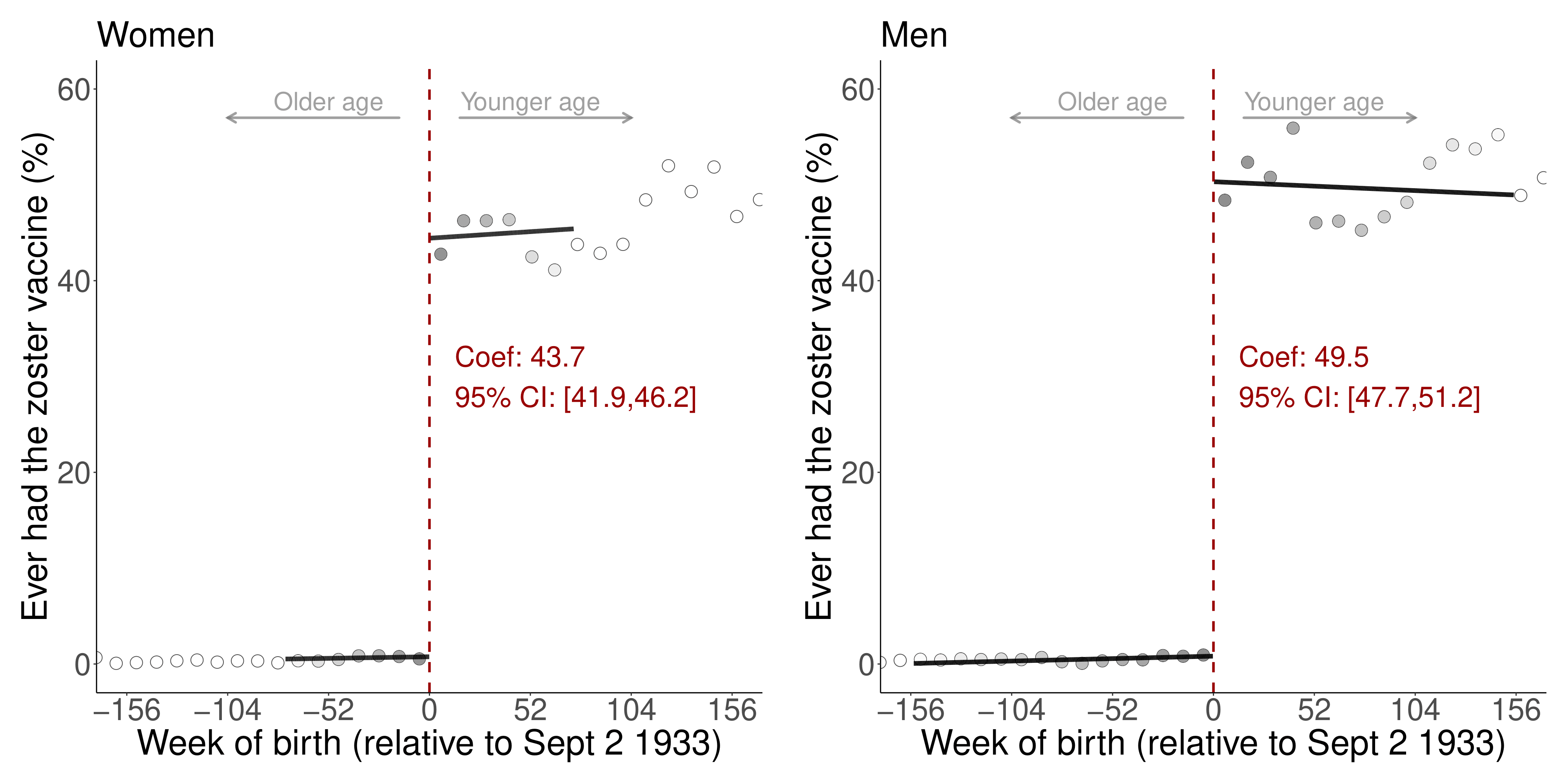


**Fig. 24**: The magnitude of the abrupt change in vaccine uptake at the September 2 1933 date-of-birth eligibility threshold was similar between men and women.^1,2,3,4^

^1^ The data source for this analysis was the SAIL database for Wales.

^2^ Grey dots show the mean value for each 10-week increment in week of birth.

^3^ The grey shading of the dots is in proportion to the weight that observations from this 10-week increment received in the analysis.

^4^ For women, the mean squared error-optimal bandwidth is 74.3 weeks (25,286 adults). For men, the mean squared error-optimal bandwidth is 154.7 weeks (43,230 adults).

Abbreviations: Coef=coefficient; CI=95% confidence interval; Sept=September.


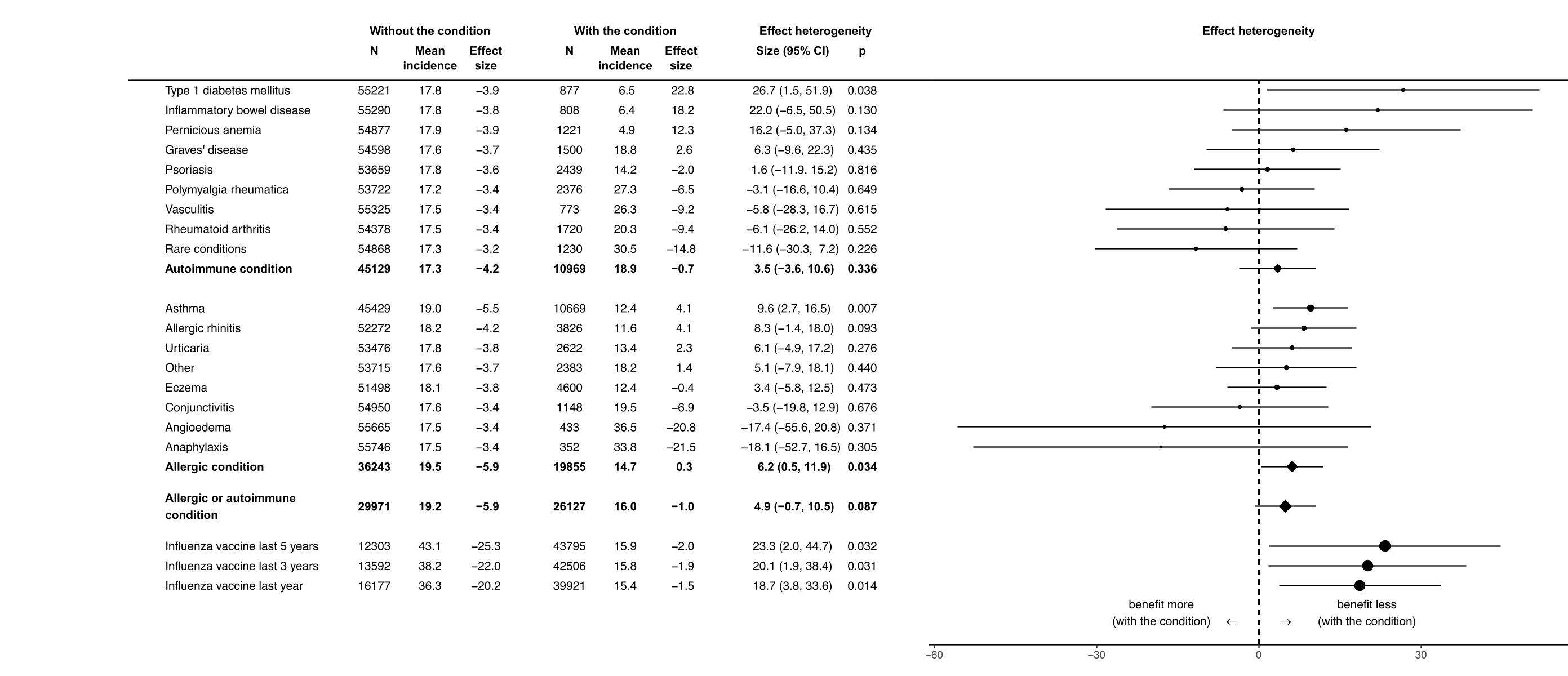


**Fig. 25**: Heterogeneity of effect estimates of receiving the vaccine between those with versus without an autoimmune or allergic condition and by receipt of prior influenza vaccination.^1,2,3^

^1^ The data source for this analysis was the SAIL database for Wales.

^2^ The size of the dots and diamonds is in proportion to the number of individuals with the condition.

^3^ Error bars depict 95% confidence intervals around the point estimates of the effect heterogeneities (two-sided t-tests).


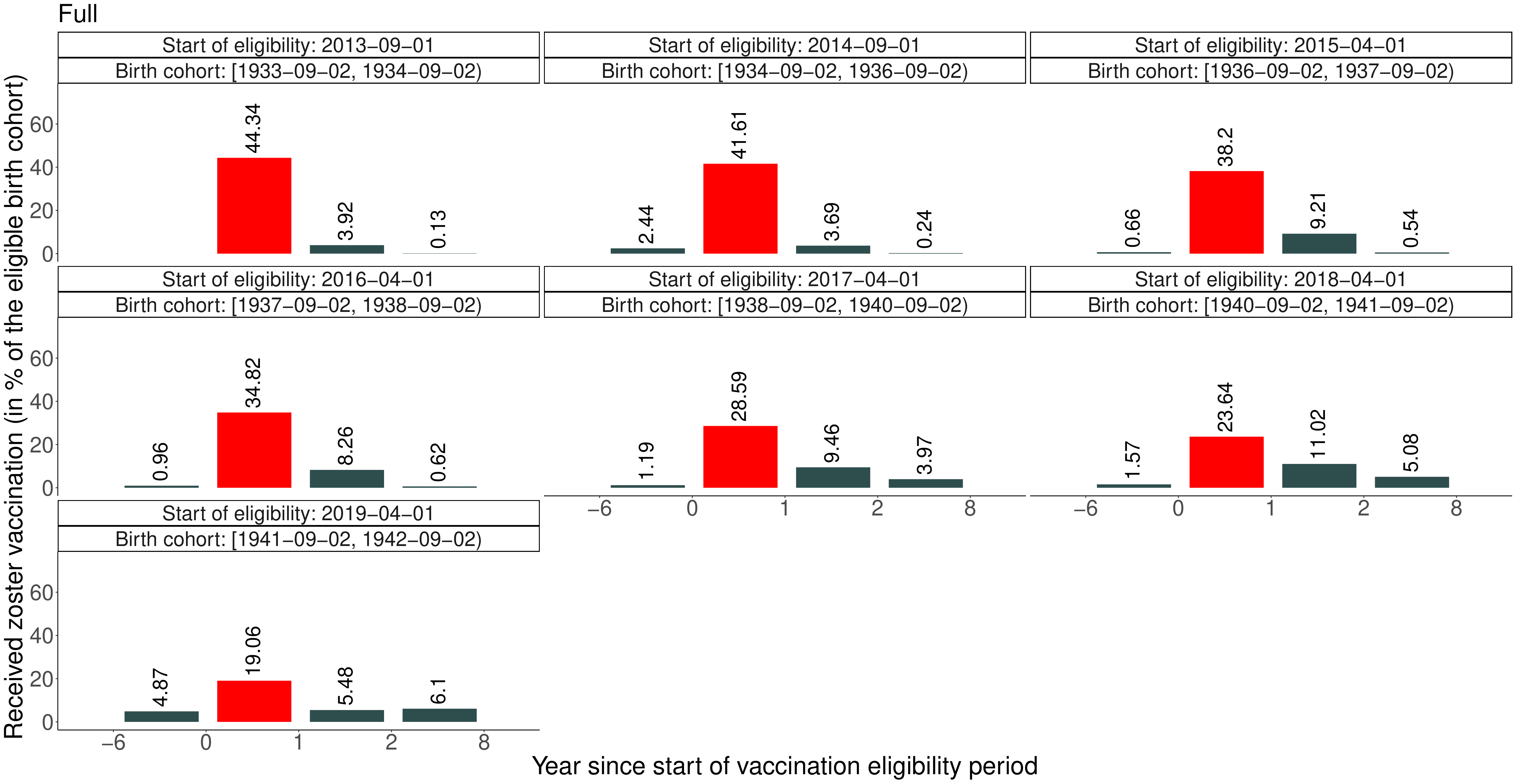


**Fig. 26:** Uptake of herpes zoster vaccination in Wales by cohort and year of eligibility.^1,2,3^

^1^ The data source for this analysis was the SAIL database for Wales.

^2^ The red bar corresponds to the respective first year of eligibility for a particular cohort.

^3^ Eligibility years 3 to 8 had to be aggregated to comply with SAIL data publication standards.

**
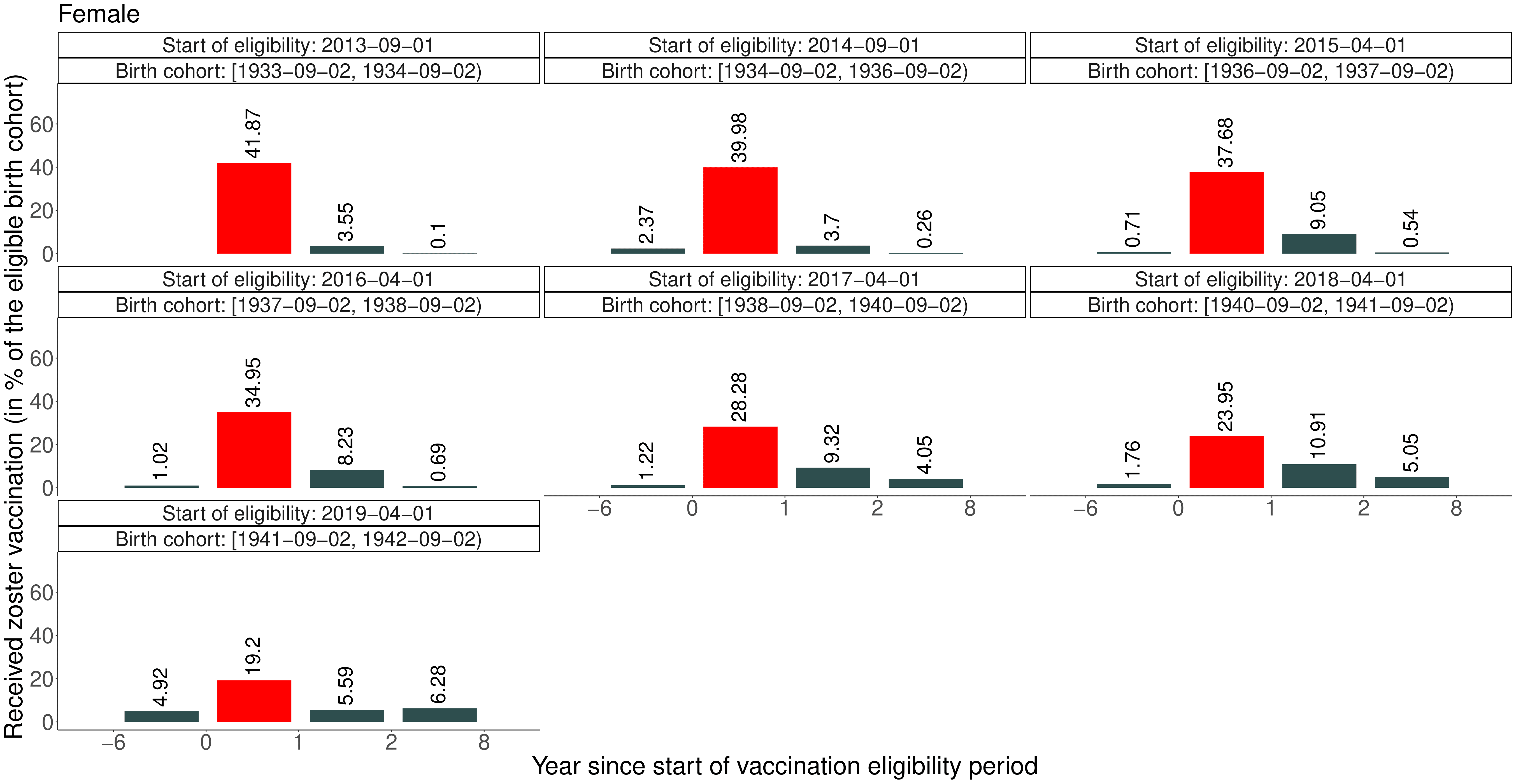
**

**Fig. 27:** Uptake of herpes zoster vaccination in Wales by cohort and year of eligibility among female patients.^1,2,3^

^1^ The data source for this analysis was the SAIL database for Wales.

^2^ The red bar corresponds to the respective first year of eligibility for a particular cohort.

^3^ Eligibility years 3 to 8 had to be aggregated to comply with SAIL data publication standards.


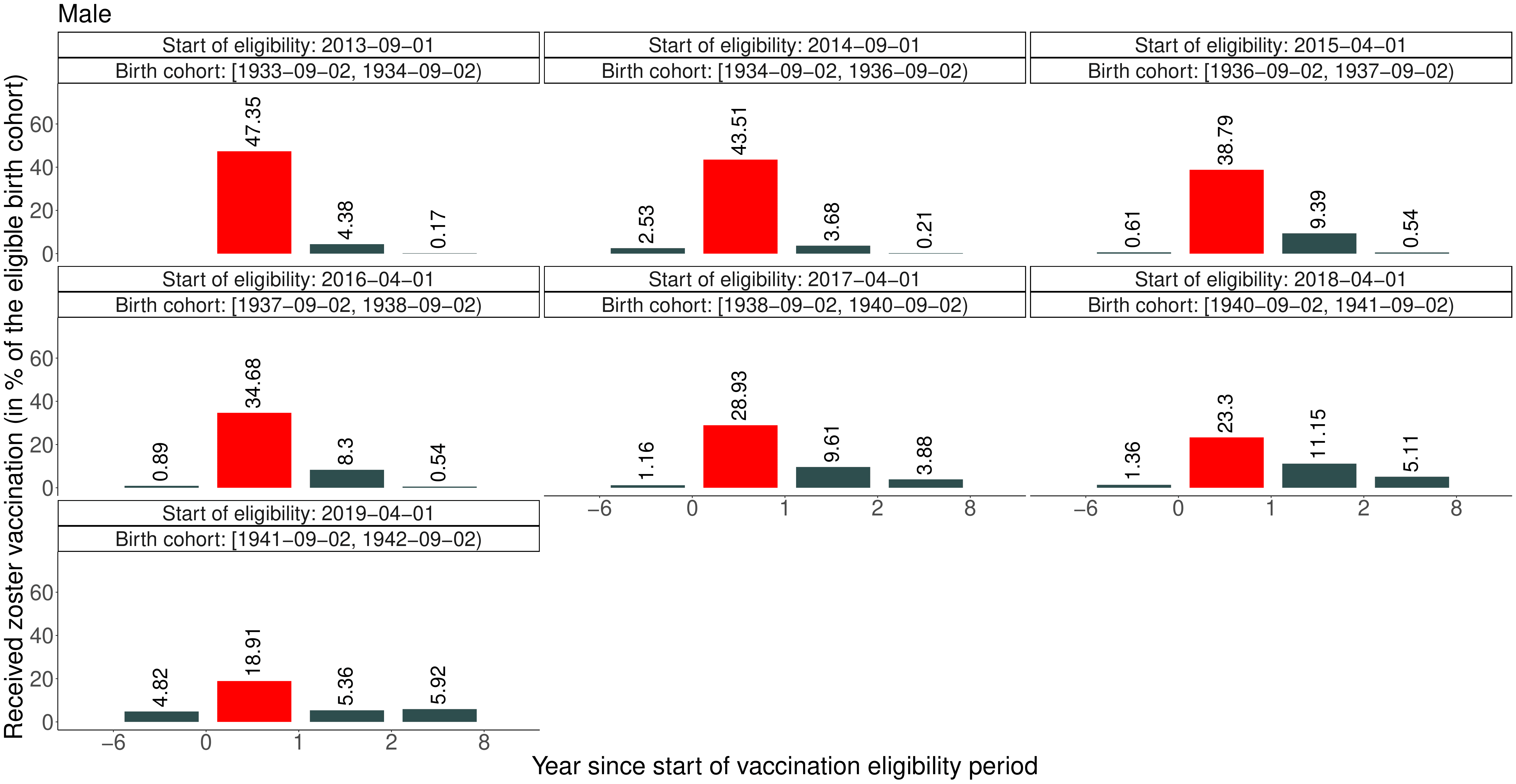


**Fig. 28:** Uptake of herpes zoster vaccination in Wales by cohort and year of eligibility among male patients.^1,2,3^

^1^ The data source for this analysis was the SAIL database for Wales.

^2^ The red bar corresponds to the respective first year of eligibility for a particular cohort.

^3^ Eligibility years 3 to 8 had to be aggregated to comply with SAIL data publication standards.


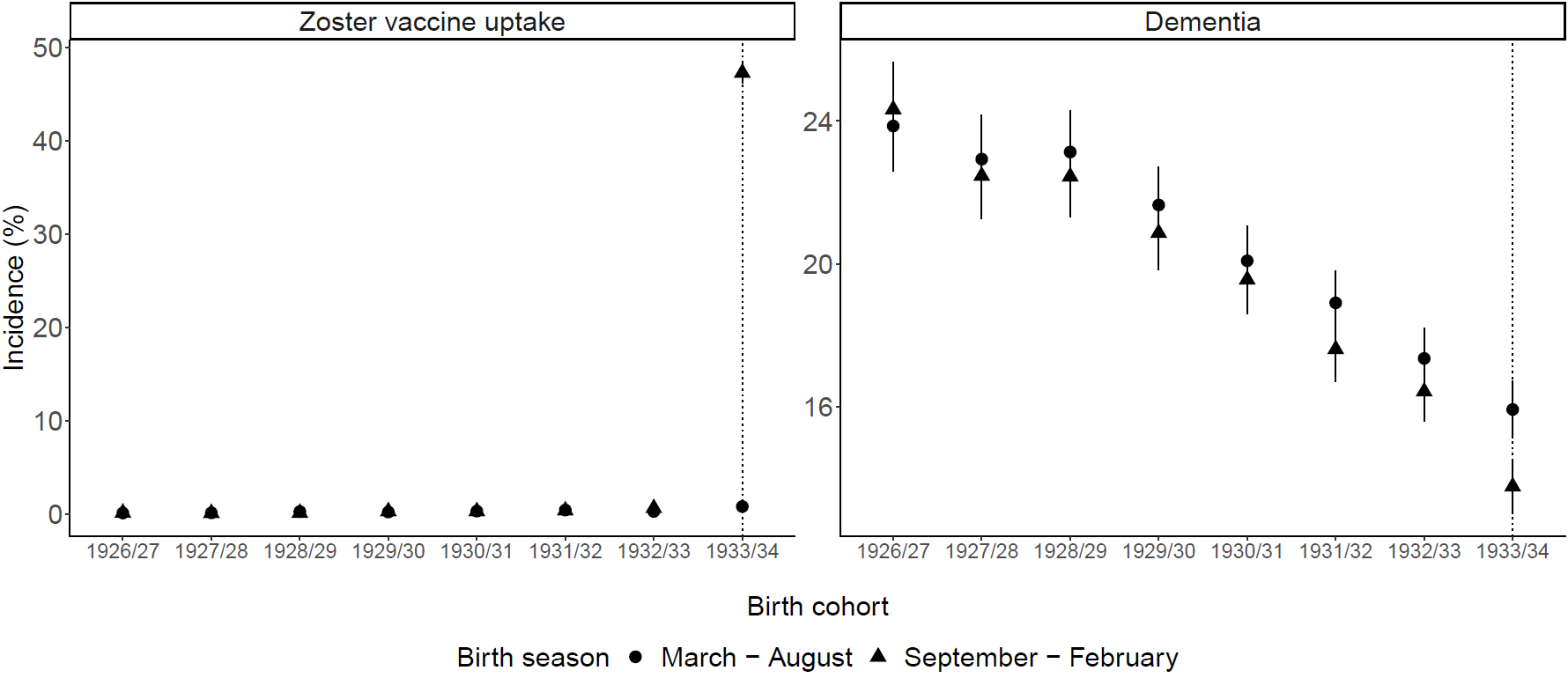


**Fig. 29:** Between-birth-season differences in vaccine uptake and dementia incidence diverge only in the 1933/1934 birth cohort.^1^

^1^ The data source for this analysis was the SAIL database for Wales.**
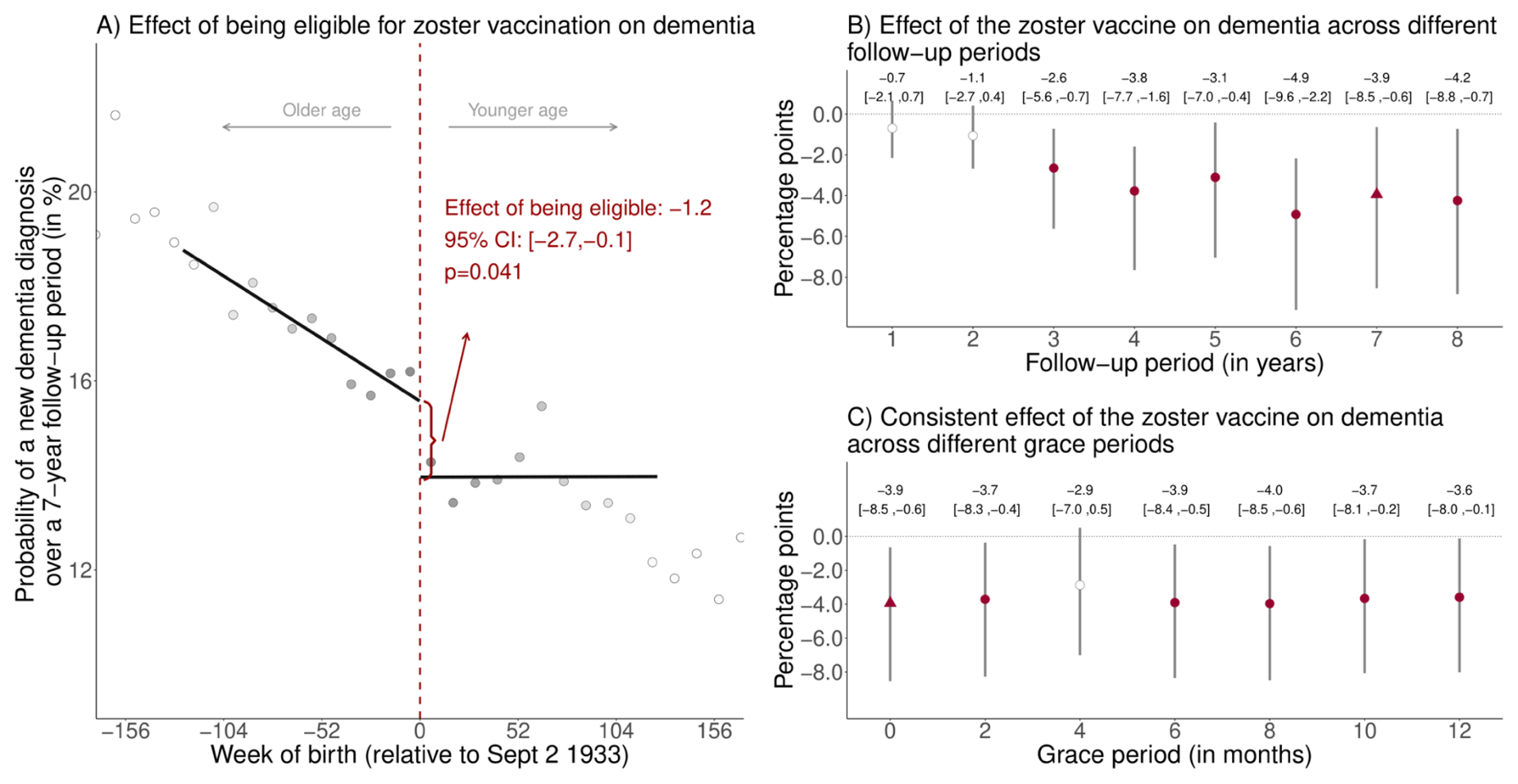
**

**Fig. 30:** Effect estimates of being eligible (A) and having received the zoster vaccine (B and C) on new diagnoses of dementia, taking into account the staggered roll-out and controlling for cohort fixed effects.^1,2,3,4,5,6,7,8^

^1^ The data source for this analysis was the SAIL database for Wales.

^2^ Instead of starting the follow-up period for all individuals on September 1 2013, we adjusted the follow-up period to account for the staggered rollout of the program by beginning the follow-up period for each individual on the date on which they first became eligible for the zoster vaccine (see Methods for details). We added cohort fixed effects to these analyses to control for between-cohort differences of the date at which the follow-up window started. That is, we defined one cohort fixed effect for ineligible individuals and the first catch-up cohort and included additional cohort fixed effects for each group of patients who became eligible at the same time.

^3^ Triangles (rather than points) depict our primary specification.

^4^ Red (as opposed to white) fillings denote statistical significance (p<0.05).
^5^ With “grace periods” we refer to time periods since the index date after which follow-up time is considered to begin to allow for the time needed for a full immune response to develop after vaccine administration.
^6^ Grey vertical bars depict 95% confidence intervals around the point estimates of the coefficients (two-sided t-tests).

^7^ Grey dots in Panel A show the mean value for each 10-week increment in week of birth.

^8^ The grey shading of the dots is in proportion to the weight that observations from this 10-week increment received in the analysis.

^9^ For panel A, the mean squared error-optimal bandwidth is 125.8 weeks (76,475 adults) and the p-value is calculated based on a two-sided t-test. For panel B and C, in our primary specification the mean squared error-optimal bandwidth is 78.6 weeks (48,078 adults).

Abbreviations: Coef=coefficient; CI=95% confidence interval; Sept=September.


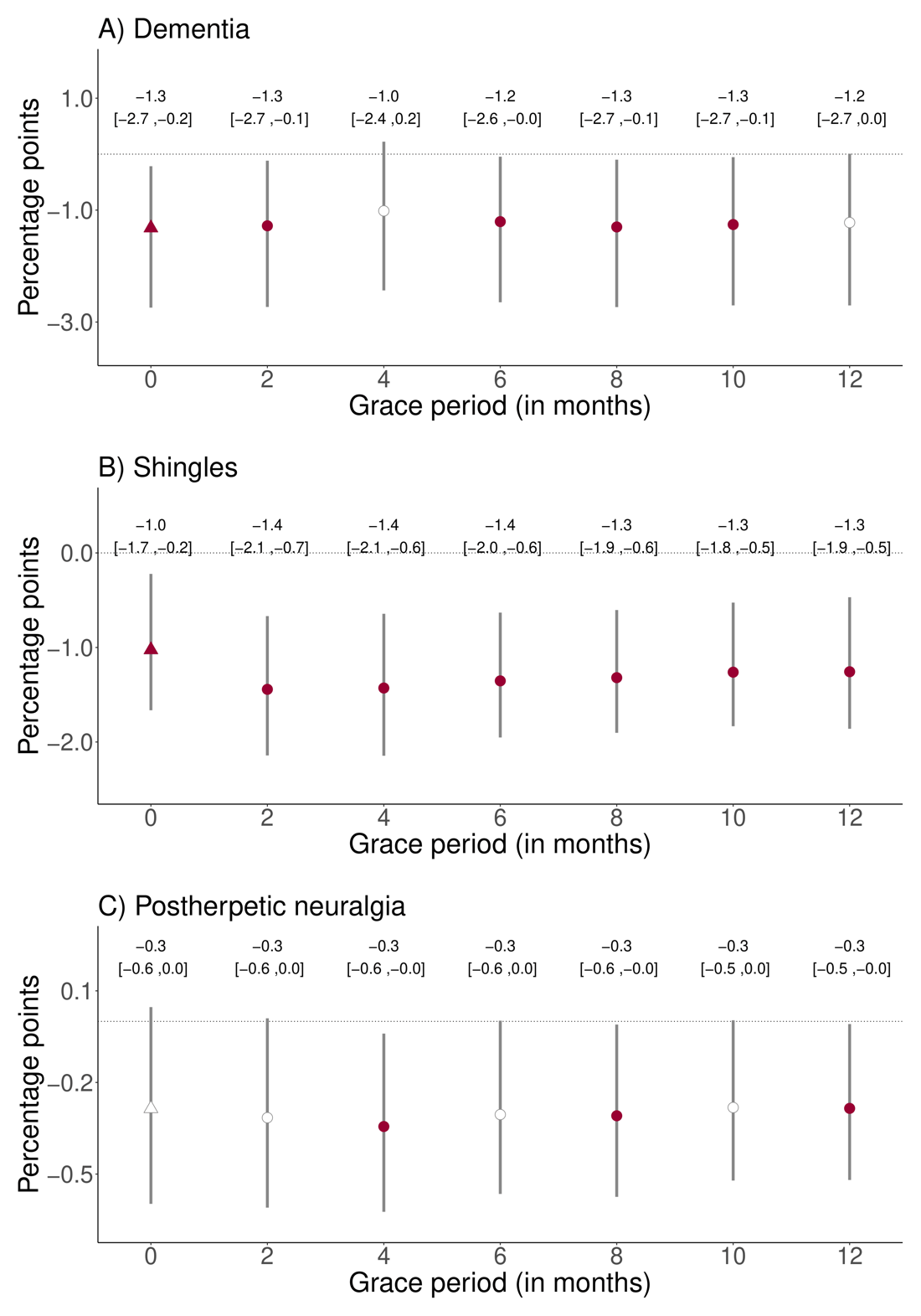


**Fig. 31:** Effect estimates of being eligible for the zoster vaccine on the probability of a new dementia diagnosis, having at least one shingles diagnosis, and having at least one diagnosis of postherpetic neuralgia across various grace periods.^1,2,3,4,5,6,7^

^1^ The data source for this analysis was the SAIL database for Wales.

^2^ With “grace periods” we refer to time periods since the index date after which follow-up time is considered to begin to allow for the time needed for a full immune response to develop after vaccine administration.

^3^ We show the same plots for the effect estimates of receipt of the zoster vaccine (as opposed to eligibility for the vaccine) in the main manuscript.

^4^ Grey vertical bars depict 95% confidence intervals around the point estimates of the coefficients (two-sided t-tests).

^5^ White points depict statistically insignificant point estimates (p>0.05).

^6^ Triangles (rather than points) depict our primary specification.

^7^ The samples consists of n=282,541 adults in panel A), and n=296,324 adults in panels B) and C).


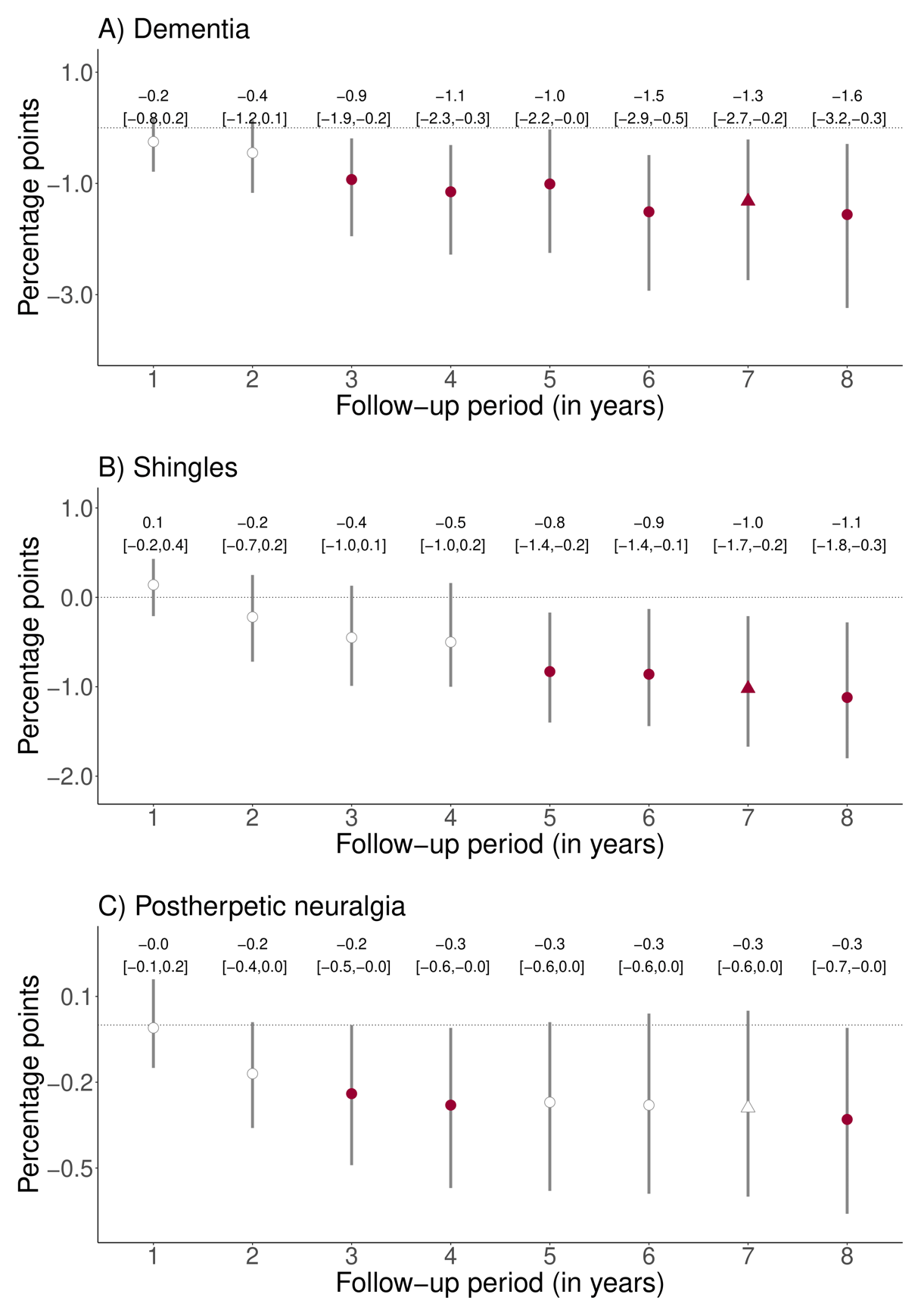


**Fig. 32:** Effect estimates of being eligible for the zoster vaccine on the probability of a new dementia diagnosis, having at least one shingles diagnosis, and having at least one diagnosis of postherpetic neuralgia across various follow-up periods.^1,2,3,4,5,6^

^1^ The data source for this analysis was the SAIL database for Wales.

^2^ We show the same plots for the effect estimates of receipt of the zoster vaccine (as opposed to eligibility for the vaccine) in the main manuscript.

^3^ Grey vertical bars depict 95% confidence intervals around the point estimates of the coefficients (two-sided t-tests).

^4^ White points depict statistically insignificant point estimates (p>0.05).

^5^ Triangles (rather than points) depict our primary specification.

^6^ The samples consists of n=282,541 adults in panel A), and n=296,324 adults in panels B) and C).

**Tables 1 to 3**

|  | |  | Full sample | |  | Female sample | |  | Male sample | | |
| --- | --- | --- | --- | --- | --- | --- | --- | --- | --- | --- | --- |
| Variable | | | % | N |  | % | N |  | % | N | |
| Vaccine eligibility | | |  |  |  |  |  |  |  |  |  |
|  | Yes | | 67.2 | 190,004 |  | 65.2 | 100,542 |  | 69.7 | 89,461 |  |
|  | No | | 32.8 | 92,537 |  | 34.8 | 53,676 |  | 30.3 | 38,861 |  |
| Vaccine receipt | | |  |  |  |  |  |  |  |  |  |
|  | Yes | | 29.8 | 84,071 |  | 28.5 | 43,931 |  | 31.3 | 40,140 |  |
|  | No | | 70.2 | 198,470 |  | 71.5 | 110,288 |  | 68.7 | 88,182 |  |
| Decile of Welsh Index of Multiple Deprivation, mean (SD) | | | 5.8 (2.8) |  |  | 5.7 (2.8) |  |  | 5.8 (2.8) |  |  |
|  | 1 (most deprived) | | 7.9 | 22,242 |  | 7.9 | 12,185 |  | 7.8 | 10,056 |  |
|  | 2 | | 9.0 | 25,420 |  | 9.1 | 14,086 |  | 8.8 | 11,334 |  |
|  | 3 | | 9.2 | 26,079 |  | 9.4 | 14,468 |  | 9.0 | 11,611 |  |
|  | 4 | | 9.8 | 27,741 |  | 9.8 | 15,158 |  | 9.8 | 12,583 |  |
|  | 5 | | 10.7 | 30,356 |  | 10.8 | 16,590 |  | 10.7 | 13,766 |  |
|  | 6 | | 10.8 | 30,555 |  | 10.8 | 16,674 |  | 10.8 | 13,881 |  |
|  | 7 | | 10.0 | 28,205 |  | 9.8 | 15,129 |  | 10.2 | 13,076 |  |
|  | 8 | | 10.5 | 29,697 |  | 10.4 | 16,081 |  | 10.6 | 13,616 |  |
|  | 9 | | 10.6 | 29,875 |  | 10.5 | 16,120 |  | 10.7 | 13,755 |  |
|  | 10 (least deprived) | | 11.4 | 32,347 |  | 11.5 | 17,714 |  | 11.4 | 14,633 |  |
| Gender | | |  |  |  |  |  |  |  |  |  |
|  | Male | | 45.4 | 128,322 |  | NA | NA |  | 100.0 | 128,322 |  |
|  | Female | | 54.6 | 154,218 |  | 100.0 | 154,218 |  | NA | NA |  |
| Clinical diagnoses | | |  |  |  |  |  |  |  |  |  |
|  | Past shingles | | 12.2 | 34,540 |  | 13.3 | 20,586 |  | 10.9 | 13,954 |  |
|  | Ischemic heart disease | | 16.3 | 46,016 |  | 11.8 | 18,167 |  | 21.7 | 27,849 |  |
|  | Chronic obstructive pulmonary disease | | 11.9 | 33,505 |  | 10.2 | 15,712 |  | 13.9 | 17,793 |  |
|  | Past stroke | | 7.6 | 21,449 |  | 6.7 | 10,284 |  | 8.7 | 11,165 |  |
|  | Past lower respiratory tract infection | | 51.8 | 146,388 |  | 52.4 | 80,735 |  | 51.2 | 65,653 |  |
|  | History of lung cancer | | 0.5 | 1,402 |  | 0.4 | 646 |  | 0.6 | 756 |  |
|  | Past fall(s) | | 19.0 | 53,581 |  | 23.7 | 36,574 |  | 13.3 | 17,007 |  |
|  | History of colorectal cancer | | 2.2 | 6,318 |  | 1.8 | 2,746 |  | 2.8 | 3,572 |  |
|  | History of lower back pain | | 45.1 | 127,518 |  | 47.3 | 72,963 |  | 42.5 | 54,555 |  |
|  | Diabetes mellitus | | 19.7 | 55,664 |  | 17.1 | 26,430 |  | 22.8 | 29,234 |  |
| Uptake of preventive health measures | | |  |  |  |  |  |  |  |  |  |
|  | Pneumococcal vaccine (PPV-23) | | 70.7 | 199,841 |  | 69.6 | 107,326 |  | 72.1 | 92,515 |  |
|  | Recent statin use | | 43.6 | 123,026 |  | 40.2 | 61,922 |  | 47.6 | 61,104 |  |
|  | Recent antihypertensive use | | 59.1 | 166,972 |  | 58.5 | 90,174 |  | 59.8 | 76,798 |  |
|  |  | | |  |  |  |  |  |  |  |  |

**Table 1: Baseline characteristics of the cohort of individuals in our primary analyses for dementia** (n=282,541 adults).^1,2,3,4,5,6^

^1^ The data source for this analysis was the SAIL database for Wales.

^2^ The baseline date was September 1 2013.

^3^ Recent use of statins and antihypertensive drugs was defined as having received a prescription (whether new or repeat) of these medications within three months prior to September 1 2013.

^4^ The clinical codes for all diagnoses are shown in the Materials section.

^5^ Deciles of the Welsh Index of Multiple Deprivation (WIMD) were calculated based on the 2011 WIMD survey.

^6^ 24 and 1 individuals had missing information for the WIMD and gender, respectively.

| A) Full sample | |  | |  | |  | |  | |  | |  |
| --- | --- | --- | --- | --- | --- | --- | --- | --- | --- | --- | --- | --- |
|  | Main | | Dementia defined by prescription | | Restricted to frequent GP visitors | | Controlled for health service utilization | | Accounting for staggered roll-out | | Quadratic fit | Controlled for dementia risk factors |
|  | (1) | | (2) | | (3) | | (4) | | (5) | | (6) | (7) |
| CACE | -3.5 | | -1.9 | | -3.0 | | -3.4 | | -3.9 | | -3.8 | -3.4 |
| 95% CI | [-7.1, -0.6] | | [-4.2, -0.1] | | [-6.7, -0.2] | | [-7.0, -0.6] | | [-8.5, -0.6] | | [-7.5, -0.8] | (-7.0, -0.5) |
| p | 0.019 | | 0.044 | | 0.036 | | 0.021 | | 0.023 | | 0.015 | 0.023 |
| bandwidth | 90.6 | | 89.5 | | 89.8 | | 90.4 | | 78.6 | | 170.1 | 88.0 |
| N | 56,098 | | 55,502 | | 47,517 | | 56,098 | | 48,078 | | 105,232 | 54,520 |
|  |  | |  | |  | |  | |  | |  |  |
| ITT | -1.3 | | -0.8 | | -1.2 | | -1.3 | | -1.2 | | -1.8 | -1.2 |
| 95% CI | [-2.7, -0.2] | | [-1.7, -0.1] | | [-2.8, 0.1] | | [-2.7, -0.2] | | [-2.7, -0.1] | | [-3.6, -0.3] | (-2.6, -0.2) |
| p | 0.022 | | 0.02 | | 0.059 | | 0.025 | | 0.040 | | 0.017 | 0.025 |
| bandwidth | 134.4 | | 139.9 | | 124.6 | | 139.8 | | 125.8 | | 159.4 | 144.2 |
| N | 83,167 | | 86,676 | | 65,478 | | 86,676 | | 76,475 | | 98,939 | 89,219 |
|  |  | |  | |  | |  | |  | |  |  |
|  |  | |  | |  | |  | |  | |  |  |
| B) Women | | |  | |  | |  | |  | |  |  |
|  | (1) | | (2) | | (3) | | (4) | | (5) | | (6) | (7) |
| CACE | -5.6 | | -3.8 | | -5.8 | | -5.5 | | -5.9 | | -6.8 | -5.5 |
| 95% CI | [-10.0, -2.5] | | [-7.6, -2.1] | | [-10.8, -2.8] | | [-9.8, -2.4] | | [-11.8, -2.4] | | [-12.1, -2.7] | (-9.8, -2.3) |
| p | 0.001 | | 0.001 | | 0.001 | | 0.001 | | 0.003 | | 0.002 | 0.001 |
| bandwidth | 149.1 | | 125.3 | | 129.4 | | 147.4 | | 116.3 | | 204.4 | 145.7 |
| N | 50,816 | | 42,649 | | 37,794 | | 50,361 | | 39,142 | | 70,051 | 49,674 |
|  |  | |  | |  | |  | |  | |  |  |
| ITT | -2.9 | | -1.9 | | -3.4 | | -2.9 | | -2.6 | | -3.4 | -2.7 |
| 95% CI | [-5.3, -1.3] | | [-3.4, -0.9] | | [-6.1, -1.6] | | [-5.2, -1.2] | | [-4.8, -0.9] | | [-6.1, -1.4] | (-5.0, -1.1) |
| p | 0.001 | | 0.001 | | 0.001 | | 0.001 | | 0.004 | | 0.002 | 0.002 |
| bandwidth | 95.5 | | 96.7 | | 90.1 | | 96.6 | | 107.8 | | 147.9 | 99.8 |
| N | 32,601 | | 33,094 | | 26,400 | | 32,946 | | 36,422 | | 50,491 | 34,080 |
|  |  | |  | |  | |  | |  | |  |  |
|  |  | |  | |  | |  | |  | |  |  |
| C) Men |  | |  | |  | |  | |  | |  |  |
|  | (1) | | (2) | | (3) | | (4) | | (5) | | (6) | (7) |
| CACE | -0.1 | | 0.7 | | 1.4 | | -0.1 | | -0.3 | | -0.2 | 0.0 |
| 95% CI | [-4.4, 4.1] | | [-1.7, 3.5] | | [-1.7, 5.2] | | [-4.4, 4.1] | | [-5.1, 4.3] | | [-5.5, 4.7] | (-4.3, 4.2) |
| p | 0.948 | | 0.491 | | 0.314 | | 0.953 | | 0.862 | | 0.874 | 0.982 |
| bandwidth | 91.8 | | 87.4 | | 147 | | 92.2 | | 89.6 | | 136.4 | 92.5 |
| N | 25,563 | | 24,353 | | 34,529 | | 25,563 | | 24,606 | | 37,849 | 25,737 |
| ITT | 0.1 | | 0.1 | | 0.9 | | 0.1 | | 0 | | 0.1 | 0.1 |
| 95% CI | [-1.9, 2.1] | | [-0.9, 1.2] | | [-1.0, 3.1] | | [-1.9, 2.1] | | [-2.1, 2.0] | | [-2.3, 2.2] | (-1.8, 2.1) |
| p | 0.935 | | 0.754 | | 0.335 | | 0.941 | | 0.956 | | 0.96 | 0.897 |
| bandwidth | 121.3 | | 146.9 | | 131.7 | | 120.7 | | 118.3 | | 189.3 | 121.7 |
| N | 33,725 | | 40,972 | | 30,706 | | 33,452 | | 32,173 | | 52,906 | 33,829 |

**Table 2: Additional robustness checks.** The CACE (complier average causal effect) refers to the estimated effect of actually receiving the zoster vaccine rather than merely being eligible for the vaccine. The ITT (intent-to-treat) effect refers to the estimated effect of being eligible for the zoster vaccine^1^

^1^ The data source for this analysis was the SAIL database for Wales

In (1), we show the results from our primary analysis for comparison (i.e., the identical results as shown in Fig. 3 in the main manuscript). In each of the other columns, we implemented the identical analysis as for our primary analysis, except for the following differences.

In (2), we defined dementia solely as a new prescription of donepezil hydrochloride, galantamine, rivastigmine, memantine hydrochloride, or idebenone.

In (3), we restricted the analysis cohort to only those individuals who had visited their primary care provider at least once in each of the five years preceding the start date of the zoster vaccine program.

In (4), we adjusted our regressions for the following indicators of health service utilization during the follow-up period: the probability of receiving at least one influenza vaccination and the number of i) primary care visits, ii) outpatient visits, and iii) hospital admissions.

In (5), instead of using September 1 2013 as the index date, we set the index date as the date when each date-of-birth cohort first became eligible for the zoster vaccine (see Methods for details) and adjusted our regressions for cohort fixed effects. These results are also shown in Extended Data Fig. 38.

In (6), we used local squared regression instead of local linear regression. These results are also shown in Extended Data Fig. 8.

In (7), adjusted our regressions for all input variables to the Dementia Risk Score.

Abbreviations: CACE=complier average causal effect (i.e., the estimated effect of actually receiving the zoster vaccine rather than merely being eligible for the vaccine); CI=robust bias-corrected confidence interval; p=p value; ITT=intent-to-treat effect (i.e., the estimated effect of being eligible for the zoster vaccine); N=sample size; GP=general practitioner

|  | Dementia | Shingles | Postherpetic neuralgia |
| --- | --- | --- | --- |
|  |  |  |  |
|  | (1) | (2) | (3) |
| Difference in CACE by gender | 6.8 | 0.7 | -0.4 |
| 95% CI | (1.2, 12.4) | (-2.2, 3.7) | (-1.6, 0.9) |
| p | 0.018 | 0.626 | 0.563 |
|  |  |  |  |
| Bandwidth (in weeks) | 90.6 | 116.9 | 96.6 |
| N | 56,098 | 76,316 | 63,039 |

**Table 3: Difference (in percentage points) between women and men in the effect of receipt of the zoster vaccine on new diagnoses of dementia, having at least one shingles diagnosis, and having at least one diagnosis of postherpetic neuralgia.** The CACE (complier average causal effect) refers to the estimated effect of actually receiving the zoster vaccine rather than merely being eligible for the vaccine. The reference group is women when calculating the difference in CACE. Observations refer to the number of observations within the optimal bandwidth.^1^

^1^ The data source for this analysis was the SAIL database for Wales.

Abbreviations: CACE=complier average causal effect (i.e., the estimated effect of actually receiving the zoster vaccine rather than merely being eligible for the vaccine); CI=confidence interval; p=p-value.
