# Supplementary material for: A natural experiment on the effect of herpes zoster vaccination on dementia
Source: Nature. 2025 Apr 2;641(8062):438–46. doi: 10.1038/s41586-025-08800-x (PMC12058522; doi:10.1038/s41586-025-08800-x)
Supplement: Supplementary file 2 — Reporting Summary [file 41586_2025_8800_MOESM2_ESM.pdf]

nature portfolio

Corresponding author(s): Pascal Geldsetzer

Last updated by author(s): Jan 22, 2025

Reporting Summary

Nature Portfolio wishes to improve the reproducibility of the work that we publish. This form provides structure for consistency and transparency in reporting. For further information on Nature Portfolio policies, see our Editorial Policies and the Editorial Policy Checklist.

Statistics

For all statistical analyses, confirm that the following items are present in the figure legend, table legend, main text, or Methods section.

n/a	Confirmed	
☐	☒	The exact sample size (*n*) for each experimental group/condition, given as a discrete number and unit of measurement
☒	☐	A statement on whether measurements were taken from distinct samples or whether the same sample was measured repeatedly
☐	☒	The statistical test(s) used AND whether they are one- or two-sided<br>*Only common tests should be described solely by name; describe more complex techniques in the Methods section.*
☐	☒	A description of all covariates tested
☐	☒	A description of any assumptions or corrections, such as tests of normality and adjustment for multiple comparisons
☐	☒	A full description of the statistical parameters including central tendency (e.g. means) or other basic estimates (e.g. regression coefficient) AND variation (e.g. standard deviation) or associated estimates of uncertainty (e.g. confidence intervals)
☐	☒	For null hypothesis testing, the test statistic (e.g. *F*, *t*, *r*) with confidence intervals, effect sizes, degrees of freedom and *P* value noted<br>*Give P values as exact values whenever suitable.*
☒	☐	For Bayesian analysis, information on the choice of priors and Markov chain Monte Carlo settings
☒	☐	For hierarchical and complex designs, identification of the appropriate level for tests and full reporting of outcomes
☐	☒	Estimates of effect sizes (e.g. Cohen's *d*, Pearson's *r*), indicating how they were calculated

Our web collection on statistics for biologists contains articles on many of the points above.

Software and code

Policy information about availability of computer code

Data collection	The manuscript did not use software for data collection.
Data analysis	RStudio with R version 4.1.3. All medical codes are available in the Supplementary information. All packages including version numbers for version control, algorithms to define variables and R analysis code are provided in an OSF repository under https://osf.io/cfnr6/?view_only=d3774e4fda2649e2b2031431b1234874

For manuscripts utilizing custom algorithms or software that are central to the research but not yet described in published literature, software must be made available to editors and reviewers. We strongly encourage code deposition in a community repository (e.g. GitHub). See the Nature Portfolio guidelines for submitting code & software for further information.

Data

Policy information about availability of data

All manuscripts must include a data availability statement. This statement should provide the following information, where applicable:

- Accession codes, unique identifiers, or web links for publicly available datasets
- A description of any restrictions on data availability
- For clinical datasets or third party data, please ensure that the statement adheres to our policy

The data that supports the findings are electronic health record data routinely collected in the UK care system via the Secure Anonymised Information Linkage

Databank (SAIL Databank). Researchers must request access to the data directly from SAIL. The authors have no permission to share the data.

Research involving human participants, their data, or biological material

Policy information about studies with human participants or human data. See also policy information about sex, gender (identity/presentation), and sexual orientation and race, ethnicity and racism.

Reporting on sex and gender	The analyses to explore effect mechanisms in our study are based on the gender variable as recorded in the SAIL database. While acknowledging that these data may not fully reflect the identity of patients, we consider gender to be the appropriate term for our study.
Reporting on race, ethnicity, or other socially relevant groupings	The population of interest consists of all adults born between September 1 1925 and August 31 1942 who were registered with a primary care provider at the start of the program (September 1, 2013). We describe the characteristics of the individuals in the data set in Extended Data Table 1, in terms of gender, and socioeconomic status. Socioeconomic status is based on the 2011 Welsh Index of Multiple Deprivation score, resulting in deciles ranging from 1 (= "most deprived”) to 10 (= “least deprived”).
Population characteristics	See above
Recruitment	Does not apply (full electronic health records)
Ethics oversight	Ethics approval was granted by the Information Governance Review Panel (IGRP, application number: 1306). Composed of government, regulatory and professional agencies, the IGRP oversees and approves applications to use the SAIL databank. All analyses were approved and considered minimal risk by the Stanford University Institutional Review Board on June 9 2023 (protocol number: 70277).

Note that full information on the approval of the study protocol must also be provided in the manuscript.

Field-specific reporting

Please select the one below that is the best fit for your research. If you are not sure, read the appropriate sections before making your selection.

☐ Life sciences ☒ Behavioural & social sciences ☐ Ecological, evolutionary & environmental sciences

For a reference copy of the document with all sections, see nature.com/documents/nr-reporting-summary-flat.pdf

Behavioural & social sciences study design

All studies must disclose on these points even when the disclosure is negative.

Study description	Quantitative quasi-experimental
Research sample	For our main analysis, our study population consisted of 282,541 individuals (154,218 women and 128,322 men) born between September 1 1925 and September 1 1942 who were registered with a primary care provider in Wales on the start date of the zoster vaccine program rollout (September 1 2013) and had no dementia diagnosis prior to the program rollout. The rationale for using a sample that is centered, in terms of birth dates, around the eligibility cutoff, is to maximize the sample size in each treatment group. The mean age at program start (September 1 2013) was 77.88 years old and the sample is representative for the Welsh population born between September 1 1925 and September 1 1942.
Sampling strategy	Due to the quasi-experimental/retrospective analysis strategy of routine patient data, "sampling procedure" is not an applicable category. There was no sampling, as the SAIL databank covers all registered individuals meeting our inclusion criteria and hence our data are representative for the described population.<br><br>Since there was no sampling and key parameters (e.g. compliance with the threshold, distribution of dementia measures at the threshold) were unknown a priori and the prospective sample size was unlikely to significantly limit the precision of the analysis, we refrained from doing a formal sample size calculation.
Data collection	The manuscript did not rely on primary data collection. The researchers were not blinded to the experimental condition and/or the study hypothesis.
Timing	Our retrospective analysis relies on data recorded by the NHS between 2000 and 2023.
Data exclusions	We pre-defined our retrospective research sample as adult patients, born between September 1 1925 and September 1 1942 who were registered with a primary care provider in Wales on the start date of the zoster vaccine program rollout. For the analysis of the effect on the first diagnosis of dementia, we excluded patients who already were diagnosed with dementia by September 2013, as these patients, by design, cannot experience a first diagnosis of dementia anymore. This led to the exclusion of 13,783 patients. Additionally we excluded 279 people born in the week starting on August 28 1933. Since we observe birth dates only at a weekly level, we cannot say whether these 279 people were eligible to receive the vaccine or not. We did not exclude any patients because

of other criteria or metrics.

Non-participation

Due to the quasi-experimental/retrospective analysis strategy of routine patient data, "non-participation" is not an applicable category.

Randomization

There is no active randomization in the quasi-experimental analysis that we used for this manuscript (regression discontinuity analysis). Confounding is discussed and studied extensively throughout the manuscript. The arbitrary nature of the eligibility rule implies that there cannot be any self-selection or intentional sorting and thus that patients just below and above the threshold are close to identical in their underlying and unobserved characteristics. We demonstrated that there is no distinct discontinuity in any relevant baseline measures for measures of health, frequency of GP visits, and dementia diagnosis themselves, signifying that these variables do not confound our analysis. We demonstrate this further using balance tests, empirically showing that a comprehensive set of potential confounding variables, including demographic characteristics, physiological variables, and markers of healthcare utilization are all continuously distributed across the threshold. Taken together, our analyses provide strong evidence that the necessary assumptions for a valid regression discontinuity analysis were met, meaning that patients below and above the threshold were comparable in observable as well as unobservable characteristics.

Reporting for specific materials, systems and methods

We require information from authors about some types of materials, experimental systems and methods used in many studies. Here, indicate whether each material, system or method listed is relevant to your study. If you are not sure if a list item applies to your research, read the appropriate section before selecting a response.

Materials & experimental systems

n/a	Involved in the study	
☒	☐	Antibodies
☒	☐	Eukaryotic cell lines
☒	☐	Palaeontology and archaeology
☒	☐	Animals and other organisms
☒	☐	Clinical data
☒	☐	Dual use research of concern
☒	☐	Plants

Methods

n/a	Involved in the study	
☒	☐	ChIP-seq
☒	☐	Flow cytometry
☒	☐	MRI-based neuroimaging

Plants

Seed stocks

Not applicable, this section showed up despite selecting "n/a" for "Plants" above.

Novel plant genotypes

Not applicable

Authentication

Not applicable
